# Synthesis and Evaluation of the 4-Substituted 2-Hydroxy-5-Iodochalcones and Their 7-Substituted 6-Iodoflavonol Derivatives for Inhibitory Effect on Cholinesterases and β-Secretase

**DOI:** 10.3390/ijms19124112

**Published:** 2018-12-18

**Authors:** Malose J. Mphahlele, Emmanuel N. Agbo, Samantha Gildenhuys

**Affiliations:** 1 Department of Chemistry, College of Science, Engineering and Technology, University of South Africa, Private Bag X06, Florida 1710, South Africa; Tagboen@unisa.ac.za; 2 Department of Life & Consumer Sciences, College of Agriculture and Environmental Sciences, University of South Africa, Private Bag X06, Florida 1710, South Africa; gildes@unisa.ac.za

**Keywords:** 4-substituted 2-hydroxy-5-iodochalcones, 7-substituted 6-iodoflavonols, X-ray, cholinesterases, BACE-1, molecular docking

## Abstract

A series of 2-aryl-3-hydroxy-6-iodo-4*H*-chromen-4-ones substituted at the 7-position with a halogen atom (*X* = F, Cl and Br) or methoxy group and their corresponding 4-substituted 2-hydroxy-5-iodochalcone precursors were evaluated in vitro for inhibitory effect against acetylcholinesterase (AChE), butyrylcholinesterase (BChE) and β-secretase (BACE1) activities. Although moderate inhibitory effect was observed for the chalcones against AChE, derivatives **2h**, **2j** and **2n** exhibited significant inhibitory effect against BChE and BACE-1. The 2-aryl-7-fluoro-8-iodoflavonols **3b** and **3c**, on the other hand, exhibited increased activity and selectivity against AChE and reduced effect on BACE-1. The flavonols **3h**, **3i**, **3k**, **3l** and **3p** exhibited moderate inhibitory effect against AChE, but significant inhibition against BChE. Compounds **2j** and **3l** exhibited non-competitive mode of inhibition against BACE-1. Molecular docking predicted strong interactions with the protein residues in the active site of BACE-1 implying these compounds bind with the substrate. Similarly docking studies predicted interaction of the most active compounds with both CAS and PAS of either AChE or BChE with mixed type of enzyme inhibition confirmed by kinetic studies.

## 1. Introduction

The chalcones [1] and the corresponding chromone-based derivatives [2] are common structural components in various compounds with anti-Alzheimer properties. Alzheimer’s disease (AD) is one of the most severe forms of dementia characterized by progressive loss of memory, cognitive impairment or inability to learn and abnormal behavior, which tends to affect the elderly people throughout the world [3]. The World Health Organization has predicted that neurodegenerative diseases will overtake cancer to become the second most prevalent cause of death after cardiovascular diseases [4,5]. Strategies currently pursued for the treatment of AD include inhibiting cholinesterases, targeting amyloid-β (Aβ) peptides and metal-Aβ complexes [6]. The use of acetylcholinesterase (AChE) inhibitors is still one of the most efficient approaches for the treatment of the symptoms of Alzheimer’s disease. The main physiological function of AChE is the splitting of acetylcholine (ACh), a mediator of cholinergic synapses during the transduction of nerve impulses [7]. Four of the six drugs currently approved by the U.S. Food and Drug Administration (FDA) for treating AD, namely, tacrine, donepezil, rivastigmine, and galantamine increase the amount of acetylcholine neurotransmitter in the brain by inhibiting the action of AChE [8,9]. However, their efficiency as inhibitors at alleviating AD symptoms is modest and these drugs have also been found to lead to side effects such as toxicity, short half-life, and non-selective inhibition of acetylcholinesterase [8,9]. Another example of a cholinesterase is butyrylcholinesterase (BChE), which is synthesized in the liver and its main function is to hydrolyze ester-containing drugs and to scavenge cholinesterase inhibitors including potent organophosphorus nerve agents before they reach their synaptic targets [10]. BChE inhibition has been found to result in improved cognitive potential with elevated levels of ACh in the brain [11]. Inhibition of BChE becomes more relevant as the disease progresses, since the activity of AChE gradually decreases. AChE and BChE are thus important as therapeutic targets in early stages and advanced stages of the AD. High levels of AChE or BChE, on the other hand, have been found to have a role in amyloid beta(Aβ)-peptide aggregation during the early stages of senile plaque formation as well as in other pathological characteristics of AD [12,13]. Studies confirmed that β-site amyloid precursor protein cleaving enzyme 1 (BACE1) plays a significant role in the cleavage of amyloid precursor protein (APP), which leads to the production of α,β-peptide [14]. Overproduction of Aβ by BACE1 results in toxic fibrils causing neurodegeneration, one of the major causes of histological hallmarks of Alzheimer’s disease [15] and this makes this enzyme another target for AD treatment.

Synthetic chalcones and chromone- or flavone-based compounds have demonstrated beneficial effects against Alzheimer’s disease in a variety of cell culture and animal models [16]. We became interested in the design and synthesis of halogenated chalcones and flavonol derivatives for further studies of biological activity as potential anti-Alzheimer agents. This interest was prompted by the fact that the presence of halogen atom/s in a molecule hold the promise of effective drug design. They increase the membrane permeability and hence improve oral absorption to fill hydrophobic cavities in the protein binding site, they facilitate the blood–brain barrier crossing, and also prolong the lifetime of the drug [17]. Moreover, the strong electron-withdrawing effect of the halogen atoms help in forming hydrogen and/or halogen bonds that help to stabilize the interactions of drug molecules with their protein targets [18,19,20,21]. Based on these considerations, we designed and synthesized series of the 2-hydroxychalcones substituted with iodine at position-5 and another halogen atom (*X* = F, Cl, Br) or methoxy group at position 4 of ring-A and transform them into the corresponding 7-halogeno/methoxy substituted 6-iodo-3-flavonol derivatives. The main aim was to evaluate both groups of compounds for their ability to inhibit AChE and/or BChE activities as well as β-secretase (BACE-1) activity through enzymatic assays. The experimental results are complemented with molecular docking studies into the active sites of these enzymes to predict the hypothetical protein–ligand binding modes.

## 2. Results and Discussion

### 2.1. Chemistry

**Scheme 1:**
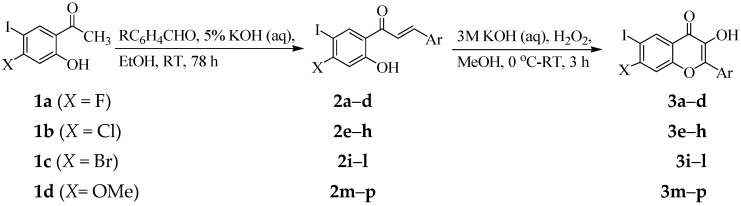
Synthesis of the 2-hydroxy-5-iodochalcones **2a**–**p** and their flavonol derivatives **3a**–**p**.

**Figure 1:**
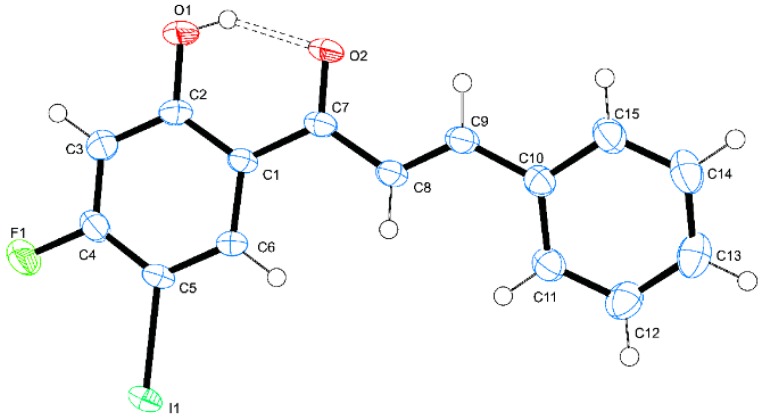
Oak Ridge Thermal Ellipsoid Plot (ORTEP) diagram (50% probability level) of **2a**. For clarity, hydrogen atoms are not labeled.

**Figure 2:**
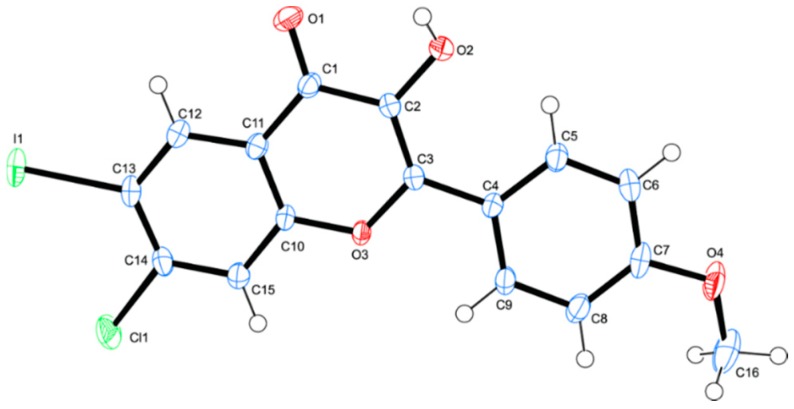
ORTEP diagram (50% probability level) of **3h**.

The synthesis of the chalcones of our interest and their conversion into the corresponding 3-flavonol derivatives have been achieved as shown in Scheme 1 and the substitution patterns on the A and B rings of these compounds are represented in Table 1. The 4-substituted 2-hydroxy-5-iodoacetophenones **1** substituted at the 4-position with different halogen atoms (*X* = F, **1a**; Cl, **1b**; Br, **1c**) or methoxy group (**1d**) were prepared via iodination of the corresponding 4-substituted 2-hydroxyacetophenones with *N*-iodosuccinimide in the presence of an equivalent amount of *p*-toluenesulfonic acid in acetonitrile at room temperature (RT) for 14 h. The 4-substituted 2-hydroxy-5-iodoacetophenones **1a**–**d** were easily distinguished from their corresponding precursors by the presence of a set of singlets in the aromatic region corresponding to H-3 and H-6, respectively. These compounds were, in turn, subjected to the base-mediated Claisen-Schmidt aldol condensation with benzaldehyde derivatives to afford the corresponding 4-halogeno/methoxy substituted 2-hydroxy-5-iodochalcones **2a**–**p**. Their ^1^H-NMR spectra revealed the presence of group of signals in the aromatic region and a singlet significantly downfield beyond δ 13.00 ppm for the intramolecularly hydrogen bonded hydroxyl proton. The chalcone nature of compounds **2a**–**p** was confirmed by the presence of a set of doublets around δ = 7.40 ppm and 7.90 ppm with coupling constant value (*J*_trans_) of 15.5 Hz, which correspond to the α-H and β-H, respectively. The *E*-geometry of the α,β-unsaturated framework was further confirmed by the single crystal X-ray diffraction (XRD) structure of **2a** (Figure 1) [22]. The XRD structure of this compound revealed the presence of strong intramolecular hydrogen bonding between the carbonyl and hydroxyl groups (O(1)-H(1)^...^O(2) = 1.77 Å). We decided to transform the chalcone *ortho*-hydroxy-*trans*-α,β-unsaturated carbonyl framework into a geometrically constraint flavonol scaffold. Consequently, we subjected the chalcone derivatives **2a**–**p** to hydrogen peroxide in the presence of 3 M aqueous potassium hydroxide in methanol at 0 °C to room temperature (RT) for 3 h to afford the corresponding 3-flavonol derivatives **3a**–**p**. The ^1^H-NMR spectra of the latter compounds lack the set of doublets corresponding to the olefinic protons observed in the spectra of the corresponding substrates. Moreover, the singlet for the 3-OH resonates significantly upfield in the region δ 9.00–10.20 ppm compared to that of the corresponding substrates. The structure of these compounds was distinctly confirmed by single crystal X-ray diffraction (XRD) analysis of compound **3h** (Figure 2) [23]. The molecule crystalized in the orthorhombic space group Pbca and there are two molecules in the unit cell. We observed the intramolecular hydrogen bond intuitively between the carbonyl oxygen and hydroxyl groups of the two molecules (O(2)-H(2)^...^O(1) = 2.39 and 2.03 Å, respectively.

With the chalcones **2a**–**p** and their geometrically constraint flavonol derivatives **3a**–**p** in hand, we proceeded to evaluate them for potential inhibitory effects against the human acetylcholinesterase (AChE) and butyrylcholinesterase (BChE) activities as well as inhibitory effect against β-secretase (BACE-1) activity.

### 2.2. Biology

Compounds **2a**–**p** and **3a**–**p** were grouped into four series each, namely, **a**–**d** (*X* = F), **e**–**h** (*X* = Cl), **i**–**l** (*X* = Br) and **m**–**p** (*X* = OCH_3_) with the substituent Ar variable and representing C_6_H_5_-, 4-FC_6_H_4,_ 4-ClC_6_H_4_- and 4-MeOC_6_H_4_-, respectively, as shown in Table 2 and Table 3. The structure activity relationship (SAR) of compounds **2a**–**p** and **3a**–**p** has been rationalized with respect to the nature of substituent on the aryl substituent (Ar) and the 4-position of the relatively flexible enone framework (chalcone) or 7-position of the rigid chromenone moiety (flavonol), respectively. The compounds were first evaluated for inhibitory effect against the AChE and BChE activities in cell-free assays using the anti-Alzheimer drug, donepezil, as a reference standard. The inhibitory effects are expressed as means of IC_50_ values (the concentration that inhibits ChE by 50%) in µM from triplicate runs.

#### 2.2.1. Inhibitory Effect of Chalcones **2a**–**p** against AChE and BChE Activities

With the exception of the less active chalcones **2a** (IC_50_ = 38.78 µM) and **2p** (IC_50_ = 58.14 µM), all of the other derivatives exhibited moderate **2b** (IC_50_ = 16.60 µM) to significant inhibitory effect against the AChE activity with the IC_50_ values ranging from 9.38–11.56 µM. These IC_50_ values are twice higher than that of donepezil (IC_50_ = 4.79 µM). By contrast, some of the chalcones in the series **2a**–**p** were found to be generally more inhibiting towards the BChE activity when compared to donepezil. Within the series **2a**–**d**, only compound **2d** substituted with fluorine atom at positon 4 of ring-A and a 4-(methoxyphenyl) group at the β-carbon was found to exhibit significant inhibitory effect against BChE activity (IC_50_ = 8.49 µM) when compared to donepezil (IC_50_ = 6.04 µM). A combination of the 4-chloro atom on ring-A and a strongly pi-electron donating 4-methoxyphenyl group at the β-carbon of **2h** resulted in increased inhibitory effect against the BChE activity (IC_50_ = 4.17 µM) when compared to the reference standard. The presence of a chlorine atom at position-4 of ring-A of compounds **2i**–**l** resulted in significant inhibitory effect against BChE activity in this series. A combination of the 4-bromo substituent on ring-A and a 4-fluorophenyl group at the β-position of **2j**, for example, resulted in increased inhibitory activity against BChE (IC_50_ = 5.73 µM). Increased inhibitory activity was also observed for compounds **2n** and **2p** within the series **2m**–**p** substituted with a 4-methoxy group at the 4-positon of ring-A and the observed trend in IC_50_ values is as follows: 4.89 (**2p**) > 5.91 (**2n**) > 9.52 (**2m**) > 11.63 µM (**2o**). The anti-cholinesterase activity which is in the order, OCH_3_ > F > H > Cl, is consistent with the decreasing propensity of the 2-(4-substituted phenyl) ring for pi-electron delocalization into the α,β-unsaturated framework, respectively. A combination of the lipophilic 4-methoxyphenyl group and a fluorine (**2d**), chlorine (**2h**) or methoxy (**2p**) substituent at the 4-position of ring-A seem to be more favorable for inhibitory effect against the BChE activity. Likewise, a combination of 4-fluorophenyl group at the β-carbon and a bromine atom (**2j**) or methoxy group (**2n**) at position 4 of ring-A imparted increased inhibitory effect against BChE activity when compared to donepezil. The most active chalcone derivatives against the BChE tend to be moderately inhibiting against AChE. Their modest selectivity values in our view may make them potential dual inhibitors against both enzymes.

#### 2.2.2. Inhibitory Effect of Flavonols **3a**–**p** against AChE and BChE Activities

The 3-flavonol derivatives **3a**–**p** were also evaluated for inhibitory effect against the two enzymes and the results are represented in Table 3. From the results of the analysis of inhibitory effects of these chromenone derivatives against AChE activity (Table 3), it is evident that a combination of a fluorine atom at the 7-position of ring-A and a 2-(4-fluorophenyl) group in **3b** (IC_50_ = 3.23 µM) or 2-(4-chlorophenyl) ring in **3c** (IC_50_ = 3.35 µM) has a vital role in the inhibition of AChE when compared to donepezil (IC_50_ = 4.79 µM). These compounds tend to be selective towards AChE since they presented reduced anti-BChE activity. The strong electron-withdrawing substituents of fluorine and chlorine at the para position of the 2-aryl group of **3b** and **3c** are probably responsible for the observed increased AChE inhibition in comparison to **3a** and **3d**. This observation is in line with the literature precedent, which showed that the 4-halogenophenyl substituted compounds exhibited increased inhibitory effect against AChE activity when compared to the unsubstituted analogues [24]. Enhanced inhibitory effect due to halogen substituents has also been observed in various series of AChE inhibitors and is explained in terms of additional hydrogen bonding or dipole-dipole interactions, and/or increased van der Waals interactions [25]. The presence of chlorine atom at the 7-position of ring-A of **3e**–**h** resulted in significantly reduced inhibitory effect for these compounds against AChE activity. The presence of a bromine atom at the 7- position of ring-A of compounds **3i**–**l**, on the other hand, imparted moderate to significant inhibitory effect against the AChE activity and the observed trend in activity is in the order, **3i** (H) > **3j** (F) > **3l** (OCH_3_) > **3k** (Cl), with the following trend in IC_50_ values: 9.96 > 11.16 > 28.99 > 29.24 µM, respectively. It seems the presence of a bulky 2-aryl group on the 7-bromo-6-iodochromenone framework is not favorable for inhibitory effect against the AChE activity as observed for compounds **3k** and **3l**. The 7-methoxyflavonol derivative **3o** substituted with a 2-(4-chlorophenyl) group on the chromenone framework was found to be the least active (IC_50_ = 55.27 µM) within the series **3m**–**p**. The other derivatives within this series were found to be relatively more inhibiting and the observed trend in inhibitory activity is as follows: **3n** (6.15 µM) > **3p** (7.15 µM) > **3m** (9.25 µM).

The enzyme inhibitory assays of the flavonol derivatives **3a**–**p** against BChE activity revealed that the 7-fluoro substituted derivatives **3a**–**d** are generally less or not active against this enzyme’s activity. However, the presence of chlorine at position 7 of ring-A in compounds **3e**–**h** resulted in moderate to significant inhibitory effect against the BChE activity and the trend in IC_50_ values is as follows: **3h** (5.72 µM) > **3e** (6.64 µM) > **3g** (7.69 µM) > **3f** (18.17 µM). The inhibitory effect in the case of the derivatives within this series seems to be influenced by the electron-donating inductive effect of the substituent at the para position of the 2-aryl ring in the order, -OCH_3_ > H > Cl > F. The trend in inhibitory effect against the BChE for the 7-bromo-6-iodoflavonol derivatives **3i**–**l**, on the other hand, is as follows: **3l** (-OCH_3_, 4.88 µM) > **3k** (Cl, 5.25 µM) > **3i** (H, 5.76 µM) > **3j** (F, 27.01 µM). Larger substituents at the para position of the 2-aryl ring in this series have therefore exhibited better activities in comparison to those with the smaller size. The inhibitory effect of compounds **3m**–**p** against BChE activity appear to be influenced by the propensity of the 2-aryl ring for pi-electron delocalization into the chromenone framework (4-CH_3_OC_6_H_4_- > 4-FC_6_H_4_- > C_6_H_5_- > 4-ClC_6_H_4_-) and the trend in IC_50_ values and therefore inhibitory effect is as follows: **3p** (3.29 µM) > **3n** (7.93 µM) > **3m** (10.51 µM) > **3o** (13.71 µM). The presence of the strongly pi-electron donating 4-CH_3_OC_6_H_4_- group at the 2-position of the chromenone framework appears to be favorable for increased inhibitory effect against BChE activity. Though strongly inhibiting against this enzyme’s activity, compound **3p** has modest selectivity and therefore potential to also act as a dual inhibitor against both AChE and BChE activities. Dual inhibition of AChE and BChE has become an efficient strategy to alleviate AD’s symptoms with minimal or no side effects [26].

#### 2.2.3. Inhibitory Activities of Compounds **2h**, **2j**, **2n**, **2p**, **3b**, **3c**, **3l** and **3p** against BACE-1

β-Secretase (BACE1) is the key enzyme in Aβ peptide generation and anti-Aβ aggregation, and inhibition of its activity represents a promising approach for the treatment of Alzheimer’s disease. We decided to evaluate the most active chalcones (**2h**, **2j**, **2n** and **2p**) and flavonols (**3b**, **3c**, **3l** and **3p**) for inhibitory effect against β-site amyloid precursor protein cleaving enzyme 1 (BACE1) using quercetin as a reference standard (Table 4). Quercetin previously showed significant inhibitory effect against BACE-1 and reduced the levels of Aβ in neurons [27]. The strongly inhibiting chalcone derivatives **2h**, **2j** and **2n** against BChE activity exhibited moderate to significant inhibitory effect against BACE-1 when compared to quercetin (IC_50_ = 12.66 µM) with IC_50_ values of 13.82, 4.70 and 25.07, respectively. The presence of 4-bromo atom and 4-fluorophenyl group on the *ortho*-hydroxy-*trans-*α,β-unsaturated carbonyl framework of **2j** resulted in increased inhibitory effect against BACE-1 (IC_50_ = 4.70 µM) activity, more so than the geometrically constraint flavonol derivatives. Compound **2j** which also exhibited increased inhibitory effect against BChE activity has potential to serve as a dual inhibitor of these enzymes. Although chalcone **2p** exhibited increased inhibitory effect against BChE, this compound was found to be poorly inhibiting against BACE-1 activity with IC_50_ value of 50.79 µM. The flavonol derivatives **3b** and **3c**, which exhibited increased inhibitory effect and selectivity against AChE activity, on the other hand, were found to exhibit moderate inhibitory effect against BACE-1 with IC_50_ values of 32.19 and 19.69 µM, respectively. Significant inhibitory effect against BACE-1 was also observed for compound **3l** (IC_50_ = 15.74 µM), which also exhibited significant inhibitory effect against BChE activity. The most active flavonol **3p** against BChE was found to be less inhibiting against BACE-1 activity when compared to the reference standard with IC_50_ = 22.44 µM.

#### 2.2.4. Enzyme Kinetic Analysis of the Most Active Compounds for ChEs and BACE-1

**Figure 3:**
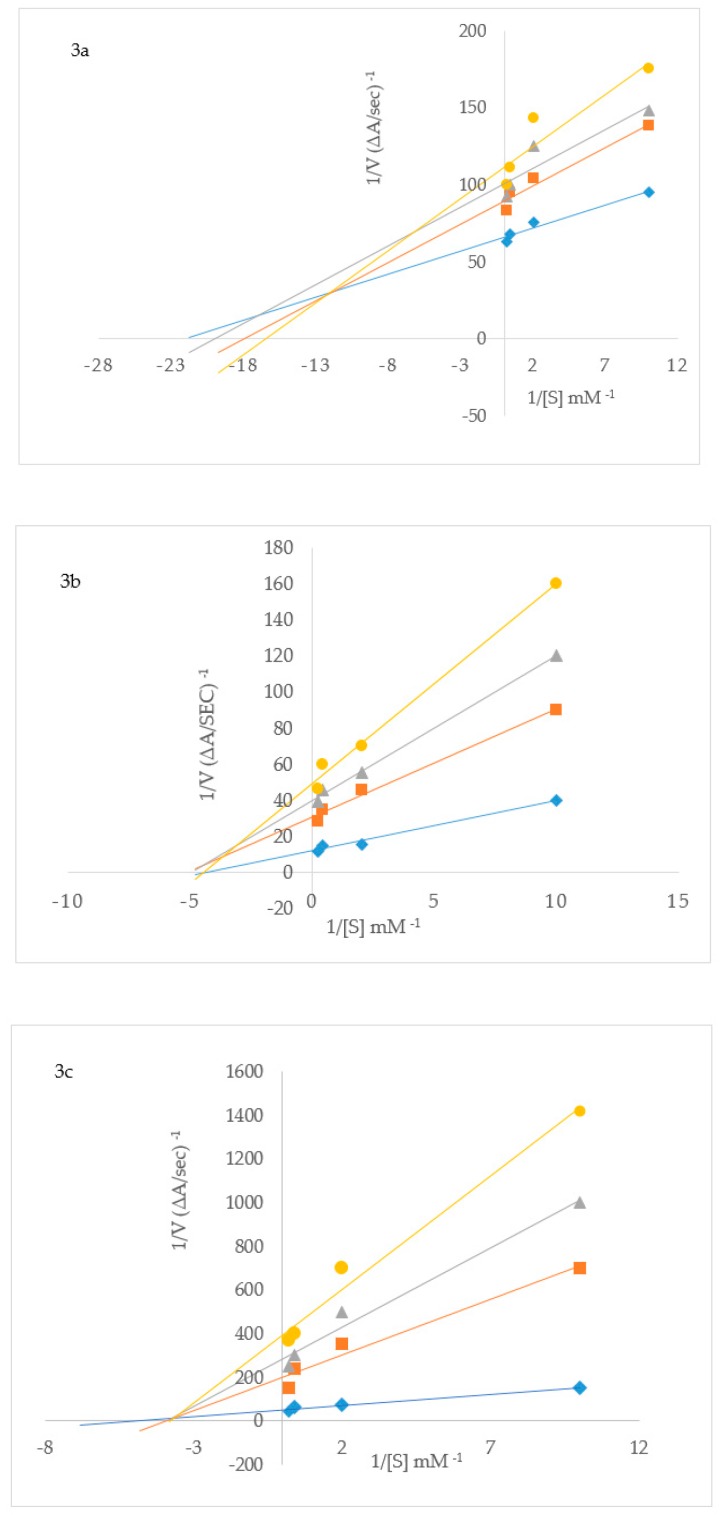
Lineweaver–Burk plots for inhibition of AChE by **3b** (**a**) and BChE by **2h** (**b**) and **3p** (**c**). Blue symbols and fitted straight lines represent enzyme activity in the absence of inhibitor, while orange (2.5 µM), grey (3.5 µM) and yellow (5 µM) represent various concentrations of inhibitor.

**Figure 4:**
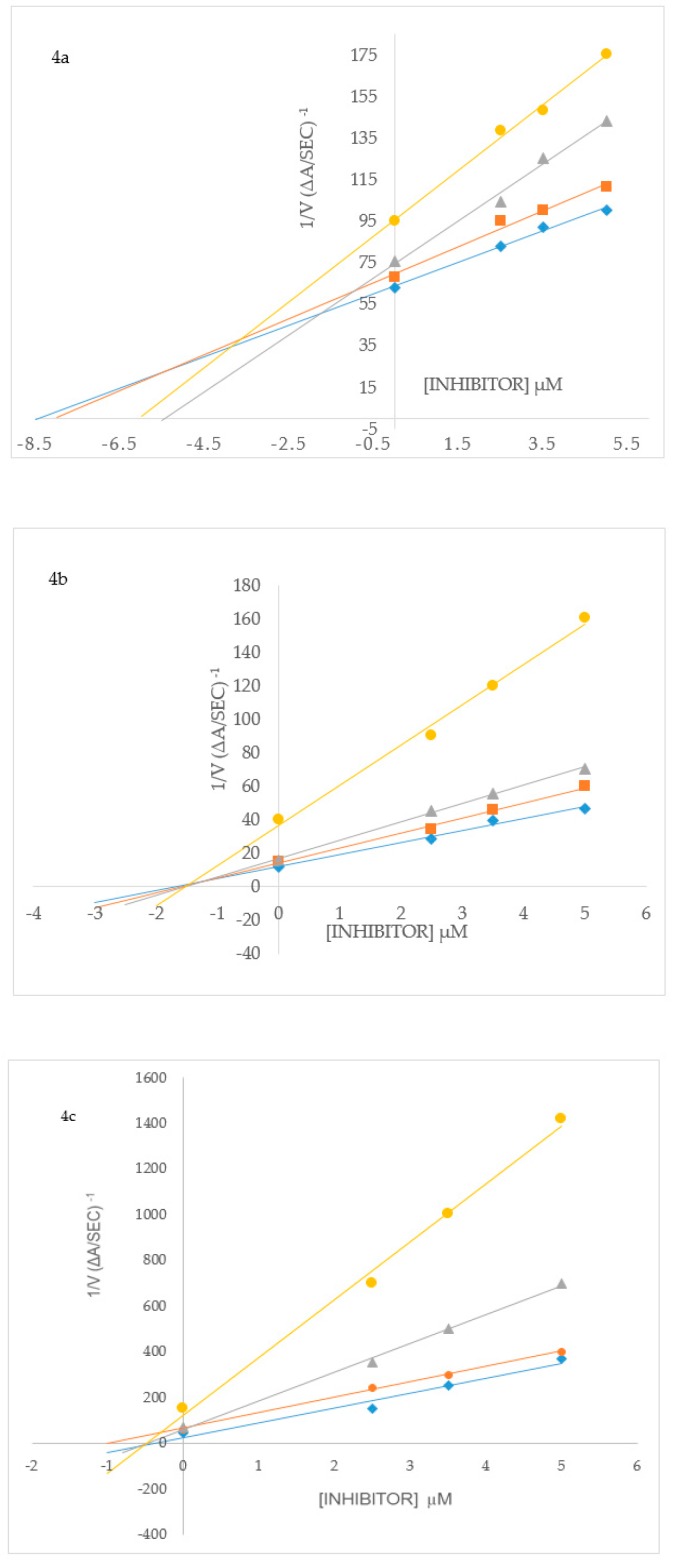
Dixon plots for inhibition of AChE by **3b** (**a**) and BChE by **2h** (**b**) and **3p** (**c**). Blue symbols and fitted straight lines represent enzyme activity with 5 mM substrate, while orange (2.5 mM), grey (0.5 mM) and yellow (0.1 mM) represent various concentrations of substrate.

**Figure 5:**
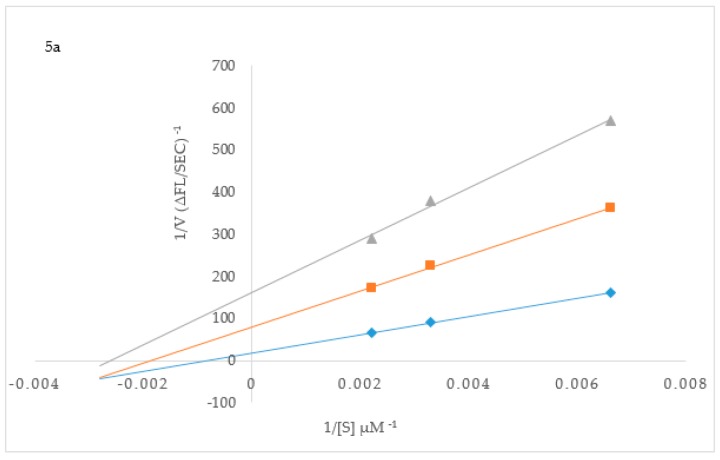

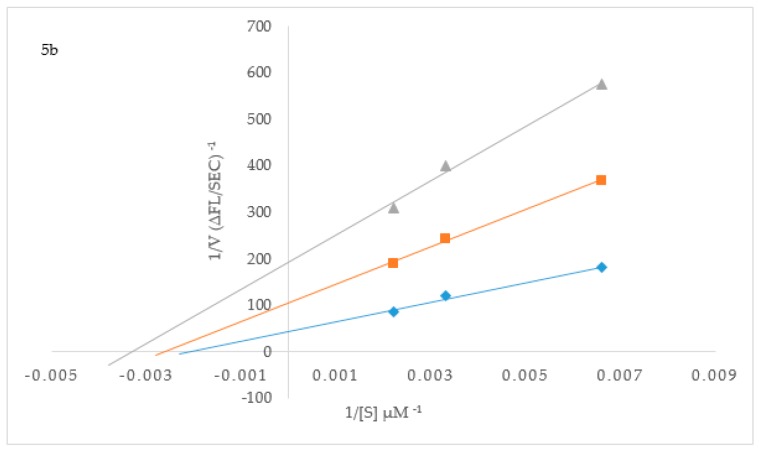
Lineweaver–Burk plots for inhibition of BACE-1 by **2j** (**a**) and **3l** (**b**), respectively. Blue symbols and fitted straight lines represent enzyme activity in the absence of inhibitor, while orange (4 µM) and grey (8 µM) represent various concentrations of inhibitor.

**Figure 6:**
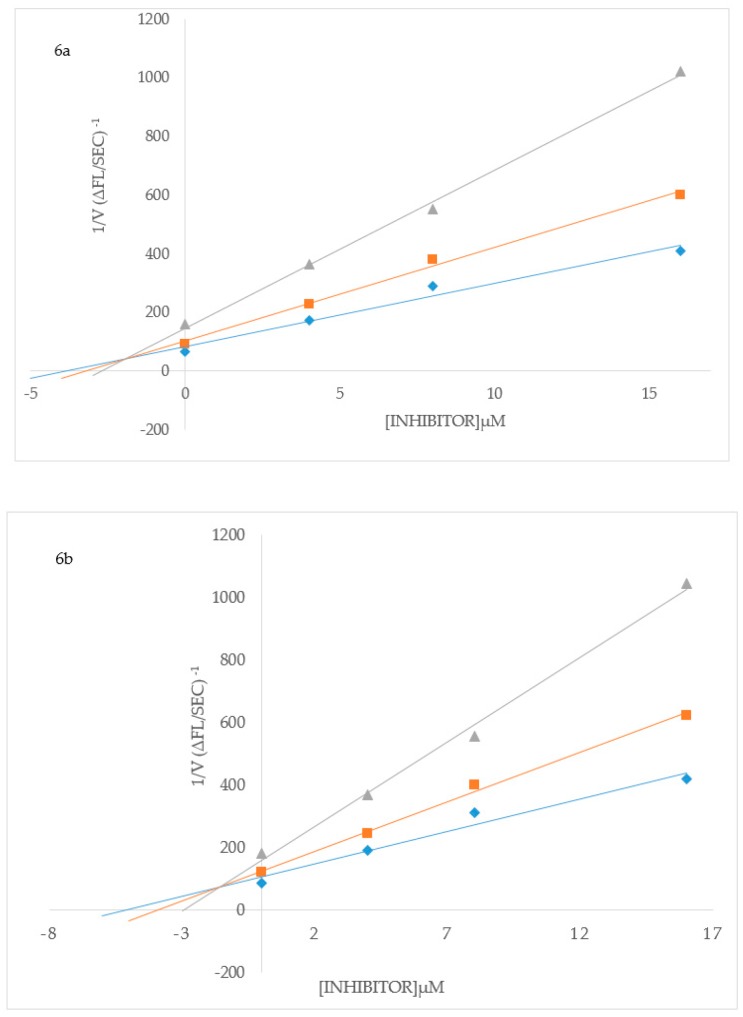
Dixon plots for inhibition of BACE-1 by **2j** (**a**) and **3l** (**b**), respectively. Blue symbols and fitted straight lines represent enzyme activity in the presence of 450 nM substrate and orange and grey, 300 nM and 150 nM substrate, respectively.

To elucidate the mechanisms of ChE and BACE-1 inhibition, kinetic studies of enzyme activity were performed. Compound **3b** which exhibited the highest inhibitory effect and selectivity against AChE activity and the highest active compounds **2h** and **3p** against BChE activity were selected for the kinetic study on increasing concentrations (0.1, 0.5, 2.5 and 5 µM) of the substrates, acetylthiocholine iodide (ATChI) and butyrylthiocholine iodide (BTChI), respectively. The linear Lineweaver–Burk equation of the Michaelis–Menten was applied to evaluate the inhibition profile and the plots of inverse of the velocity of the reaction versus inverse of the substrate concentration are represented in Figure 3. Molecules that bind only to the catalytic active site (CAS) or only to the peripheral anionic site (PAS) of the cholinesterase enzymes (AChE or BChE) are classified as competitive or non-competitive inhibitors, respectively [28]. If molecules can bind AChE or BChE at CAS or PAS sites and to additional sites on the enzymes, while still allowing the substrates AChI or BChI to bind then their mode of inhibition is considered to be mixed type [28]. Plot of inverse of the velocity of the reaction versus inverse of the substrate concentration (Figure 3) suggests that **3b** (Figure 3a) and **3p** (Figure 3c) decrease the V_max_ of AChE and BChE respectively, and increase the Michaelis constant (K_m_) of the enzymes. The K_m_ values for **3b** at 0–5.0 µM are 0.05 and 0.06, whereas for compound **3p** at the same concentration are 0.17 and 0.30, respectively. These compounds presumably also bind to other sites of the enzyme and also interact with the protein residues in the functional sites, CAS and PAS, of BChE. The Michaelis constant (K_m_) value for **2h** (K_m_ = 0.22; Figure 3b), on the other hand, remained relatively unchanged during inhibition of AChE while V_max_ decreases with increasing inhibitor concentration. This suggests non-competitive mode of inhibition against BChE activity where the compound binds BChE modulating the enzymes structure and therefore ability to catalyze the reaction while not affecting the substrates ability to bind the enzyme. Inhibition constants (*K_i_*) were determined by interpreting the Dixon plots (Figure 4). For **3b** the intercept of the straight lines or the *K_i_* value was determined to be 4.34 ± 1.1 µM (Figure 4a). An intercepting set of straight lines on the x-axis of the Dixon plot (Figure 4b) confirms that **2h** is a non-competitive inhibitor of BChE. Similarly the *K_i_* values for **2h** and **3p** are 1.36 ± 0.17 and 0.46 ± 0.16, respectively (Figure 4b,c). The lower the *K_i_* value the better the inhibitor, therefore **3p** is the best inhibitor of BChE among the two series of compounds. Based on the IC_50_ value of the chalcone **2j** and flavanol derivative **3l** against BACE-1 activity, we evaluated them for their inhibition mode toward this enzyme (Figure 5). Both compounds displayed a mixed mode of inhibition with K_m_ values affected in the presence of increasing inhibitor (4 and 8 µM) and absence of inhibitor as can be seen from the x-intercepts for both compounds in Figure 5. The V_max_ values decreased with increasing inhibitor present for **2j** and **3l**. Dixon plots (Figure 6) confirmed the mixed mode of inhibition as the intercept for all the lines occurred above the x axis. The intercept points represent the *K_i_* values to be 3.04 ± 2.31 and 1.21 ± 0.66 for **2j** and **3l** respectively, making **3l** the better inhibitor of BACE-1.

Molecular docking studies were performed on compounds **2a**–**p** and **3a**–**p** into the active sites of AChE, BChE and BACE-1 to predict their hypothetical protein–ligand binding mode.

### 2.3. Molecular Docking Studies into AChE and BChE Active Sites

**Figure 7:**
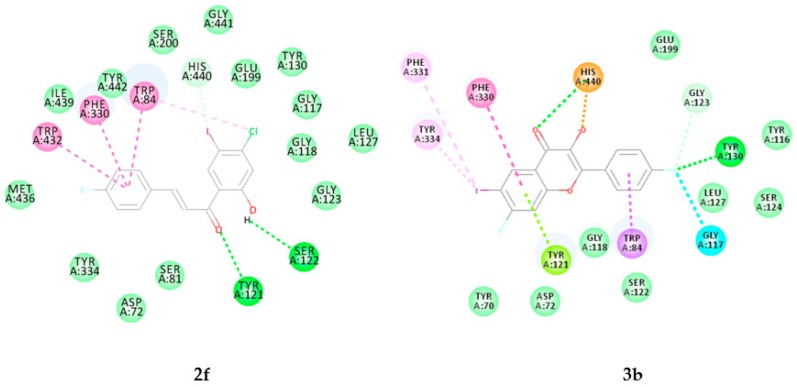
Docking poses of **2f** and **3b** into the active site of AChE. Interactions are color coded, bright green represents conventional hydrogen bonds, light green- van der Waals interactions, very light green- carbon hydrogen bonds, orange- salt bridges, blue- halogen bonds, dark pink- pi–pi interactions, light pink- pi-alkyl interactions, purple- pi-sigma interactions and green yellow- pi lone pair interactions.

**Figure 8:**
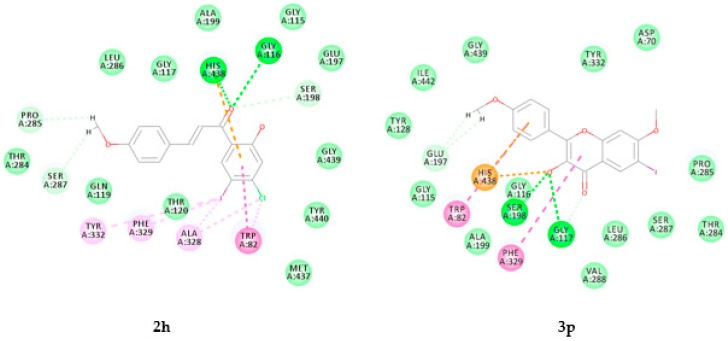
Docking poses of compounds **2h** and **3P** into the active site of BChE. Interactions are color coded, bright green represents conventional hydrogen bonds, light green- van der Waals interactions, very light green- carbon hydrogen bonds, orange- pi carbon and attractive charge interactions, dark pink- pi–pi interactions and light pink- pi-alkyl and alkyl interactions.

In order to figure out the plausible protein-ligand interactions at molecular level and to rationalize the structure activity relationship (SAR), we docked both the chalcones **2a**–**p** and flavonols **3a**–**p** into the active pockets of AChE and BChE against donepezil (refer to Figures S2 and S3 in the Supplementary Material for their docking poses). The two groups of compounds are represented herein by the docking poses of the most active compounds from each series, namely, compounds **2f** and **3b** against AChE (Figure 7) and **2h** and **3p** against BChE (Figure 8), respectively. The active site of AChE has been reported to consist of the catalytic triad (Ser200, His440, Glu327), oxyanion hole (OH, Gly118, Gly119, Ala201), anionic subsite (Trp84, Tyr121, Glu199, Gly449, Ile444), acyl binding pocket (Trp233, Phe288, Phe290, Phe292, Phe330, Phe331) and peripheral anionic subsite (Asp72, Tyr121, Ser122, Trp279, Phe331, Tyr334), which are known to be buried at the bottom of a 20 Å deep aromatic cleft [29]. The catalytic triad of BChE (PDB code: 1P0I), on the other hand, consists of serine (Ser198), histidine (His438) and glutamate (Glu325), which are interconnected by hydrogen bonds [30].

#### 2.3.1. Molecular Docking of Compounds **2f** and **3b** into AChE Active Site

**Figure 9:**
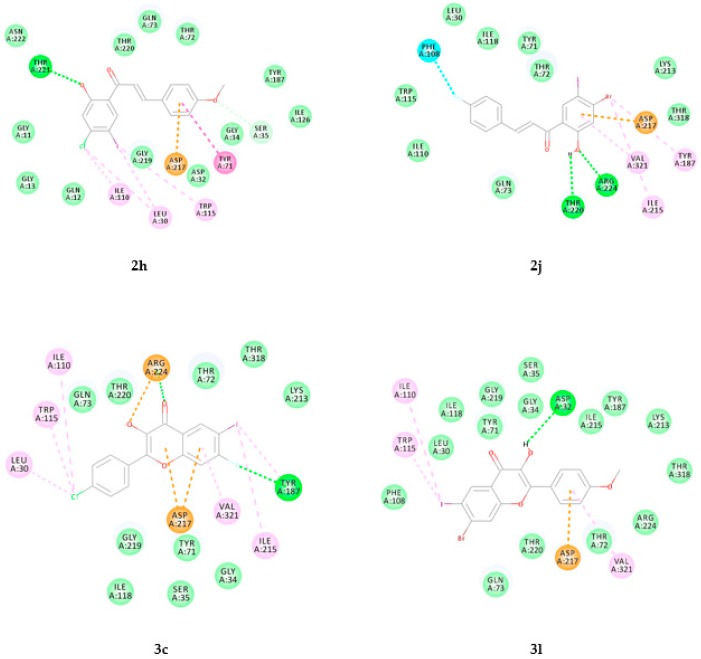
Docking poses of **2h**, **2j**, **3c** and **3l** into BACE-1. Interactions are color coded, bright green represents conventional hydrogen bonds, light green- van der Waals interactions, very light green- carbon hydrogen bonds, orange- pi-anion and attractive charge interactions, blue- halogen bonds, dark pink- pi–pi interactions and light pink- pi–alkyl and alkyl interactions.

Donepezil used as a reference standard in the experimental studies has previously been found to interact with both the catalytic active site (CAS) and the peripheral anionic site (PAS) tryptophans via ring-stacking interactions [31]. The docking pose of compound **2f** shown in Figure 9, revealed the presence of two relatively weak hydrogen bond (Hb) interactions detected between the carbonyl and hydroxyl group of **2f** with tyrosine-121 (Tyr121; Hb distance = 2.80 Å) and serine-122 (Ser122; Hb distance = 2.62 Å) on the peripheral anionic and anionic subsites, respectively. The molecule penetrates the aromatic cleft of the AChE through ring B and its entrance into the aromatic cleft was supported by π-π interaction with phenylalanine (Phe330) in the acyl binding pocket. These hydrophobic interactions are generated by the close contacts between the non-polar amino acid side chains of the enzyme and the lipophilic groups of **2f**. Although the chalcone derivatives **2a**–**p** interact with the protein residues in the peripheral anionic and acyl binding pocket of AChE, molecular docking predicted no interactions with any of the protein residues in the catalytic triad residues of AChE. The observed inhibitory effect against the AChE activity for compounds **2** is probably due to their binding to the enzyme’s peripheral anionic site.

The docking pose of **3b** with AChE (Figure 7) within the series **3a**–**p** reveals the involvement of the carbonyl group in hydrogen bonding with histidine-440 (His440, Hb distance = 2.73 Å) of the catalytic triad, which also forms a salt bridge with oxygen of the hydroxyl group. The fluorine atom on the 2-aryl ring is involved in hydrogen bonding with Tyr130 (Hb distance = 2.18 Å) and halogen bond interaction with Gly117. Pi-pi stacking and T-shaped interactions exist between the fused benzo ring and the protein residue, Phe330, in the acyl binding pocket. Ring-A is also involved in pi-lone pair with Tyr121 while its iodine atom is involved in pi-sigma interactions with the protein residues Phe331 and Tyr334. Noncovalent interactions (hydrogen bond, van der Waals, charge transfer, pi-stacking, etc.) play significant roles in the stability of the protein-ligand complexes. The interactions of compounds **3b** (Figure 7) and **3c** (see Figure S2.20 in the Supplementary Material) with these protein residues and with His440 of the catalytic triad probably account for their observed increased inhibitory effect and selectivity against the AChE activity. The predicted interaction of these compounds with protein residues in the CAS and PAS is consistent with the observed mixed inhibitory mode.

The predicted interaction of compounds **3b** and **3c** with some of the protein residues of the catalytic triad is consistent with their increased inhibitory effect and selectivity against this enzyme, while still potentially allowing substrate binding. The kinetic analysis results, suggest that these compounds exhibit a mixed type of inhibition consistent with their predicted interactions with both functional sites, CAS and PAS, as well as its acyl binding pocket.

#### 2.3.2. Molecular Docking of Compounds **2h** and **3p** into the Active Site of BChE

Figure 8 shows the most active compounds from both series, namely, **2h** and **3p** docked into BChE. The docking pose of compound **2h** predicts the presence of weak hydrophobic interactions (alkyl, pi-alkyl and pi-pi T shaped) with the protein residues Trp82 and Ala328 located at the choline binding site (or cation-π site) Hydrophobic interactions are also predicted between this compound and Tyr332 of the peripheral anionic site (PAS) as well as Phe329 in the acyl binding pocket of BChE. Intermolecular hydrogen bonding interaction is predicted between the carbonyl oxygen and Gly116 with hydrogen bond (Hb) distance of 2.98 Å. Ring-A and the carbonyl group of **2h** form pi-cation interaction and weak hydrogen bonding interaction (Hb distance = 2.54 Å) with the protein residue His438 in the catalytic triad. A weak carbon hydrogen bond interaction has also been predicted between the carbonyl group of this compound and Ser198 (Hb distance = 2.97 Å) in the catalytic triad. The observed increased activity and selectivity of compound **2h** for this enzyme probably result from the interaction of this compound with the protein residues His438 and Ser198 of the catalytic triad. Docking of the flavonol derivatives **3a**–**p** into the active site of BChE (refer to Figures S3.18–3.33 in the Supplementary Material for the docking poses) also revealed the involvement of His438. Their docking poses represented herein by that of the most active compound **3p** (Figure 8), revealed the presence of pi-cation interaction and attractive charge interaction (pi-anion) of His438 with the 2-aryl substituent and oxygen atom of the 3-hydroxyl group, respectively. The C-ring of this compound is involved in hydrophobic pi–pi T-shaped interactions with Phe329 and similar association exist between its aryl substituent and Trp82 inside the primary binding site. Its hydroxyl oxygen atom is involved in weak hydrogen bond interaction with Gly117 (Hb distance = 2.86 Å) and the protein residue, Ser198 (Hb distance = 3.03 Å) of the catalytic triad.

The predicted strong interactions of compounds **2h** and **3p** with protein residues in the acyl binding pocket or choline binding site as well as those of CAS and PAS of BChE are consistent with the observed mixed kinetic type of inhibition for these compounds. Such interaction with both protein residues of CAS and PAS accounts for the observed increased inhibitory effect and moderate selectivity against BChE activity (see Table 2).

#### 2.3.3. Docking of Chalcones (**2h**, **2j**, **2n** and **2p**) and Flavonols (**3b**, **3c**, **3l** and **3p**) into BACE-1

The docking poses of **2h**, **2j**, **3c** and **3l** into BACE-1 (PDB code: 4D8C) are represented in Figure 9 and those of compounds **2n**, **2p**, **3b** and **3p** have been included as Figure S4 in the Supplementary Information. These compounds are involved in electrostatic and hydrophobic interactions with the protein residues in the active site of BACE-1. The hydroxyl group of **2h** is also involved in hydrogen bonding interaction with Thr221 (Hb distance = 2.0 Å). Two hydrogen bond interactions are predicted between oxygen and hydrogen atoms of the hydroxyl group of **2j** with Arg224 (Hb distance = 2.12 Å) and Thr220 (Hb distance = 2.62 Å), respectively. There is also a halogen bond interaction between the fluorine atom of ring-B and the protein residue, Phe108. Ring-A is involved in pi-anion interaction with Asp217 and Asp457. These interactions may account for the observed increased inhibitory effect of **2j** against this enzyme’s activity. Two hydrogen bond interactions are predicted between the carbonyl oxygen of **3c** with Arg224 (Hb distance = 2.04 Å) and between fluorine atom on the fused benzo ring and Tyr187 (Hb distance = 2.90 Å). The protein residue, Arg224, also forms a salt bridge with the hydroxyl oxygen while the fused benzo and heterocyclic rings form a pi–cation interaction with Asp217. The hydroxyl group of the chalcone derivative **3l** is involved in hydrogen bond interaction with Asp32 (Hb distance = 2.22 Å) of the catalytic aspartic acid dyad.

## 3. Materials and Methods

### 3.1. General

The melting point values were recorded on a Thermocouple digital melting point apparatus (Mettler Toledo LLC, Columbus, OH, USA) and are uncorrected. The IR spectra, on the other hand, were recorded as powders using a Bruker VERTEX 70 FT-IR Spectrometer (Bruker Optics, Billerica, MA, USA) with a diamond ATR (attenuated total reflectance) accessory by using the thin-film method. For column chromatography, we employed Merck kieselgel 60 (0.063–0.200 mm) as stationary phase (Merck KGaA, Frankfurt, Germany). NMR spectra were obtained as DMSO-*d*_6_ solutions on a Varian Mercury 300 MHz NMR spectrometer (Varian Inc., Palo Alto, CA, USA) or Agilent 500 MHz NMR spectrometer (Agilent Technologies, Oxford, UK) and the chemical shifts are quoted relative to TMS peak. Low- and high-resolution mass spectra were recorded at an ionization potential of 70 eV using Micromass Autospec-TOF (double focusing high resolution) instrument (Waters Corp., Milford, MA, USA).

### 3.2. Typical Procedure for the Synthesis of the 4-Substituted 2-Hydroxy-5-Iodoacetophenones ***1a***–***d***

A stirred mixture of a 4-substituted 2-hydroxyacetophenone derivative (1 equiv.) and *p*-toluenesulfonic acid (1.2 equiv.) in acetonitrile (25 mL/mmol of ketone) at 0 °C was treated with *N*-iodosuccinimide (1 equiv.) over 5 min. The mixture was then allowed to stir at room temperature (RT) for 14 h and then quenched with an ice-cold saturated aqueous solution of sodium thiosulphate. The precipitate was filtered and recrystallized to afford pure 4-substituted 2-hydroxy-5-iodoacetophenone derivative as a solid. The following acetophenone derivatives were prepared in this fashion.

#### 1-(4-F.luoro-2-hydroxy-5-iodophenyl)ethanone (**1a**)

Solid (8.81 g, 90 %), mp. 87–88 °C; ATR γ_max_ (cm^−1^) 450, 544, 781, 1146, 1202, 1468, 1631; ^1^H-NMR (DMSO-*d*_6_) δ (ppm) 2.53 (3H, s, -CH_3_), 6.62 (1H, d, *J* = 9.3 Hz, H-3), 8.01 (1H, d, *J* = 7 Hz, H-6), 12.40 (1H, s, -OH); ^13^C-NMR (DMSO-*d*_6_) δ (ppm) 28.7, 70.2 (d, ^2^*J*_CF_ = 27.5 Hz), 105.2 (d, ^2^*J*_CF_ = 26.3 Hz), 120.8 (d, ^4^*J*_CF_ = 2.4 Hz), 142.3 (d, ^3^*J*_CF_ = 5.77 Hz), 163.0 (d, ^3^*J*_CF_ = 12.6 Hz), 163.4 (d, ^1^*J*_CF_ = 249.6 Hz), 202.3; HRMS (ES): MH^+^ found 340.8518. C_8_H_7_O_2_FI^+^ requires 340.8535.

#### 1-(4-C.hloro-2-hydroxy-5-iodophenyl)ethanone (**1b**)

Solid (7.39 g, 85%), mp. 143–145 °C; ATR γ_max_ (cm^−1^) 440, 519, 794, 968, 1204, 1309,1452, 1634; ^1^H-NMR (DMSO-*d*_6_) δ (ppm) 2.58 (3H. s, -CH_3_), 7.17 (1H, s, H-3), 8.18 (1H, s, H-6), 11.72 (1H, s, -OH); ^13^C-NMR (DMSO-*d*_6_) δ (ppm) 29.2, 86.0, 118.7, 123.0, 141.9, 143.8, 160.6, 201.6; HRMS (ES): MH^+^ found 294.9037. C_8_H_5_IO_2_^35^ClI^+^ requires 294.9023.

#### 1-(4-B.romo-2-hydroxy-5-iodophenyl)ethanone (**1c**)

Solid (6.90 g, 88%), mp. 157–158 °C; ATR γ_max_ (cm^−1^) 448, 620, 780, 1203, 1280, 1446, 1630; ^1^H-NMR (DMSO-*d*_6_) δ (ppm) 2.56 (3H, s, -CH_3_), 7.33 (1H, s, H-3), 8.15 (1H, s, H-6), 11.70 (1H, s, -OH); ^13^C-NMR (DMSO-*d*_6_) δ (ppm) 29.2, 83.2, 99.9, 116.0, 141.1, 163.8, 165.3, 201.8; HRMS (ES): MH^+^ found 338.8518. C_8_H_5_IO_2_^79^BrI^+^ requires 338.8535.

#### 1-(2-H.ydroxy-5-iodo-4-methoxyphenyl)ethanone (**1d**)

Solid (7.04 g, 80%), mp.156–157 °C (Lit. [32]^.^ 161 °C), ATR γ_max_ (cm^−1^) 438, 563, 751, 1175, 1220, 1442, 1619 cm^−1^; ^1^H-NMR (DMSO-*d*_6_) δ (ppm) 2.53 (3H, s, -CH_3_), 3.88 (3H, s, -OCH_3_), 6.38 (1H, s, H-3), 8.05 (1H, s, H-6), 12.67 (1H, s, -OH); cm^−1^; ^13^C-NMR (DMSO-*d*_6_) δ (ppm) 26.3, 56.7, 73.2, 100.0, 115.9, 141.1, 163.8, 165.3, 201.8; HRMS (ES): MH^+^ found 292.9518. C_9_H_10_O_3_I^+^ requires 292.9521.

### 3.3. Typical Procedure for the Synthesis of the 2-hydroxy-5-iodo-4-methoxychalcones ***2a***–***p***

A mixture of **1** (1 equiv.) and benzaldehyde derivative (1.2 equiv.) in the presence of 5% KOH (15 mL/mmol of **1**) in ethanol (30 mL/mmol of **1**) was stirred at room temperature (RT) for 78 h. The mixture was acidified dropwise with concentrated hydrochloric acid and the resultant precipitate was filtered off, washed with cold water and then recrystallized from ethanol to afford compound **2a**–**p**. The following compounds were prepared in this fashion.

#### (*E*)-1-(4-Fluoro-2-hydroxy-5-iodophenyl)-3-phenylpropenone (**2a**)

Solid (5.32 g, 81%), mp.138–139 °C; ATR γ_max_ (cm^−1^) 492, 579, 645, 730, 831, 973, 1042, 1156, 1200, 1278, 1342, 1477, 1560, 1635, 2361, 3027, 3062; ^1^H-NMR (DMSO-*d*_6_) δ (ppm) 6.72 (1H, d, *J* = 9.3 Hz, H-3), 7.43-7.44 (3H, m, H-4′ and H-2′,6′), 7.46 (1H, d, *J*_trans_ = 16.0 Hz, H-8), 7.65 (2H, t, *J* = 3.6 Hz, H-3′,5′), 7.90 (1H, d, *J*_trans_ = 16.0 Hz, H-7), 8.20 (1H, d, *J* = 7.2 Hz, H-6), 13.10 (1H, s, -OH); ^13^C-NMR (DMSO-*d*_6_) δ (ppm) 67.9 (d, ^2^*J*_CF_ = 26.3 Hz), 105.4 (d, ^2^*J*_CF_ = 25.2 Hz), 118.8, 128.6 (d, ^3^*J*_CF_ = 17.2 Hz), 128.8 (2 × C), 131.0, 133.8, 140.0 (d, ^4^*J*_CF_ = 4.5 Hz), 146.3, 165.3 (d, ^1^*J*_CF_ = 254.1 Hz), 165.6 (d, ^3^*J*_CF_ = 5.7 Hz), 191.2; HRMS (ES): MH^+^ found 368.9767. C_15_H_11_O_2_FI^+^ requires 368.9774.

#### (*E*)-1-(4-Fluoro-2-hydroxy-5-iodophenyl)-3-(4-fluorophenyl)propenone (**2b**)

Solid (5.30 g, 77%), mp. 125–126 °C; ATR γ_max_ (cm^−1^) 485, 509, 731, 766, 806, 949, 1014, 1158, 1196, 1286, 1428, 1596, 1636, 2912, 3000; ^1^H-NMR (DMSO-*d*_6_) δ (ppm) 6.75 (1H, d, *J* = 9.3 Hz, H-3), 7.15 (2H, t, *J* = 8.4 Hz, H-3′,5′), 7.42 (1H, d, *J*_trans_ = 15.3 Hz, H-8), 7.65 (2H, dd, *J* = 5.4 Hz and 9 Hz, H-2′,6′), 7.93 (1H, d, *J*_trans_ = 15.6 Hz, H-7), 8.24 (1H, d, *J* = 6.9 Hz, H-6), 13.12 (1H, s, -OH); ^13^C-NMR (DMSO-*d*_6_) δ (ppm) 68.3 (d, ^2^*J*_CF_ = 26.3 Hz), 105.4 (d, ^2^*J*_CF_ = 26.3 Hz), 114.7, 115.0, 116.4 (d, ^2^*J*_CF_ = 21.8 Hz), 118.9, 130.5 (d, ^4^*J*_CF_ = 3.45 Hz), 131.0 (d, ^3^*J*_CF_ = 8.0 Hz), 140.3 (d, ^4^*J*_CF_ = 4.6 Hz), 145.4, 164.5 (d, ^1^*J*_CF_ = 252.0 Hz), 166.1 (d, ^3^*J*_CF_ = 17.2 Hz), 191.4; HRMS (ES): MH^+^ found 386.9879. C_15_H_10_O_2_F_2_I^+^requires 386.9918.

#### (*E*)-3-(4-chlorophenyl)-1-(4-fluoro-2-hydroxy-5-iodophenyl)prop-2-en-1-one (**2c**)

Solid (6.04 g, 84%), mp.146–147 °C; ATR γ_max_ (cm^−1^) 472, 548, 760, 852, 1092, 1283, 1321, 1424, 1592, 1670, 2559, 2838; ^1^H-NMR (DMSO-*d*_6_) δ (ppm) 6.75 (1H, d, *J* = 8.7 Hz, H-3), 7.42 (2H, d, *J* = 8.0 Hz, H-3′,5′), 7.46 (1H, d, *J*_trans_ = 15.5 Hz, H-8), 7.61 (2H, d, *J* = 8.0 Hz, H-2′,6′), 7.82 (1H, d, *J*_trans_ = 15.5 Hz, H-7), 8.22 (1H, d, *J* = 6.6 Hz, H-6), 13.08 (1H, s, -OH); ^13^C-NMR (DMSO-*d*_6_) δ (ppm) 68.4 (d, ^2^*J*_CF_ = 26.3 Hz), 106.0 (d, ^2^*J*_CF_ = 26.3 Hz), 119.6, 129.3 (d, ^3^*J*_CF_ = 16.0 Hz), 130.0, 131.6, 132.6, 137.4, 140.3 (d, ^4^*J*_CF_ = 4.5 Hz), 145.2, 165.1 (d, ^1^*J*_CF_ = 254.2 Hz), 166.0 (d, ^3^*J*_CF_ = 13.7 Hz), 191.3; HRMS (ES): MH^+^ found 402.9385. C_15_H_10_O_2_F^35^ClI^+^ requires 402.9398.

#### (*E*)-1-(4-Fluoro-2-hydroxy-5-iodophenyl)-3-(4-methoxyphenyl)propenone (**2d**)

Solid (5.20 g, 73%), mp. 154–155 °C; ATR γ_max_ (cm^−1^) 448, 513, 534, 579, 664, 800, 1042, 1168, 1194, 1256, 1509, 1553, 1634, 2840, 2936; ^1^H-NMR (DMSO-*d*_6_) δ (ppm) 3.87 (3H, s, -OCH_3_), 6.75 (1H, d, *J* = 9.6 Hz, H-3), 6.95 (2H, d, *J* = 8.7 Hz, H-3′,5′), 7.36 (1H, d, *J*_trans_ = 15.0 Hz, H-8), 7.64 (2H, d, *J* = 8.7 Hz, H-2′,6′), 7.90 (1H, d, *J*_trans_ = 15.3 Hz, H-7), 8.23 (1H, d, *J* = 7.2 Hz, H-6), 13.30 (1H, s, -OH); ^13^C-NMR (DMSO-*d*_6_) δ (ppm) 55.5, 68.2 (d, ^2^*J*_CF_ = 27.5 Hz), 105.8 (d, ^2^*J*_CF_ = 25.2 Hz), 114.6, 116.6, 119.3, 126.9, 130.9, 140.1 (d, ^4^*J*_CF_ = 5.7 Hz), 146.6, 162.4, 165.6 (d, ^1^*J*_CF_ = 252.1 Hz), 166.0 (d, ^3^*J*_CF_ = 13.7 Hz), 191.5; HRMS (ES): MH^+^ found 398.9895. C_16_H_13_O_3_FI^+^ requires 398.9893.

#### (*E*)-1-(4-Chloro-2-hydroxy-5-iodophenyl)-3-phenylpropenone (**2e**)

Solid (5.12 g, 79%), mp. 150–151 °C; ATR γ_max_ (cm^−1^) 476, 523, 684, 970, 1188, 1556, 1633, 3064; ^1^H-NMR (DMSO-*d*_6_) δ (ppm) 7.12 (1H, s, H-3) 7.44 (2H, d, *J* = 4 Hz, H-3′,5′), 7.50 (1H, d, *J*_trans_ = 15 Hz, H-8), 7.68 (2H, dd, *J* = 3.6 and 7.0 Hz, H-2′,6′), 7.94 (1H, d, *J*_trans_ = 15 Hz, H-7), 8.30 (1H, s, H-6), 12.82 (1H, s, -OH); ^13^C-NMR (DMSO-*d*_6_) δ (ppm) 88.2, 119.1, 119.6, 120.6, 128.9, 129.1, 131.4, 134.1, 140.3, 145.8, 146.9, 163.7, 191.9; HRMS (ES): MH^+^ found 380.9484. C_15_H_11_O_2_^35^ClI^+^ requires 384.9492.

#### (*E*)-1-(4-Chloro-2-hydroxy-5-iodophenyl)-3-(4-fluorophenyl)propenone (**2f**)

Solid (5.90 g, 87%), mp. 144–145 °C; ATR γ_max_ (cm^−1^) 469, 507, 825, 1029, 1157, 1187, 1507, 1562, 1639, 3075; ^1^H-NMR (DMSO-*d*_6_) δ (ppm) 7.14 (2H, t, *J* = 8.5 Hz, H-3′,5′), 7.12 (1H, s, H-3) 7.41 (1H, d, *J*_trans_ = 15.5 Hz, H-8), 7.67 (2H, t, *J* = 7 Hz, H-2′,6′), 7.90 (1H, d, *J*_trans_ = 15.5 Hz, H-7), 8.27 (1H, s, H-6), 12.76 (1H, s, -OH); ^13^C-NMR (DMSO-*d*_6_) δ (ppm) 84.2, 116.3 (d, ^2^*J*_CF_ = 21.75 Hz), 118.8, 118.9, 119.7, 120.6, 130.5 (d, ^4^*J*_CF_ = 2.75 Hz), 130.9 (d, ^3^*J*_CF_ = 8.6 Hz), 140.2, 145.6, 145.9, 163.8, 164.6 (d, ^1^*J*_CF_ = 251.2 Hz), 191.7; HRMS (ES): MH^+^ found 402.9390. C_15_H_10_O_2_^35^ClIF^+^ requires 402.9398.

#### (*E*)-1-(4-Chloro-2-hydroxy-5-iodophenyl)-3-(4-chlorophenyl)propenone (**2g**)

Solid (5.65 g, 80%), mp. 190–191 °C; ATR γ_max_ (cm^−1^) 490, 795, 813, 824, 1025, 1171, 1551, 1635, 3075; ^1^H-NMR (DMSO-*d*_6_) δ (ppm) 7.20 (1H, s, H-3) 7.43 (2H, d, *J* = 7.5 Hz, H-3′,5′), 7.47 (1H, d, *J*_trans_ = 16.0 Hz, H-8), 7.62 (2H, d, *J* = 8.0 Hz, H-2′,6′), 7.90 (1H, d, *J*_trans_ = 15.5 Hz, H-7), 8.30 (1H, s, H-6), 12.72 (1H, s, -OH); ^13^C-NMR (DMSO-*d*_6_) δ (ppm) 84.3, 119.6, 119.7, 120.6, 129.5, 130.1, 132.6, 137.4, 140.2, 145.4, 146.0, 163.8, 191.6; HRMS (ES): MH^+^ found 420.0472. C_15_H_10_O_2_^35^Cl_2_I^+^requires 420.0484.

#### (*E*)-1-(4-Chloro-2-hydroxy-5-iodophenyl)-3-(4-methoxyphenyl)propenone (**2h**)

Solid (5.10 g, 73%), mp. 152–153 °C; ATR γ_max_ (cm^−1^) 517, 633, 824, 990, 1170, 1244, 1548, 1602, 1623, 3074; ^1^H-NMR (DMSO-*d*_6_) δ (ppm) 3.87 (3H. s, -OCH_3_), 7.00 (2H, d, *J* = 8.5 Hz, H-3′,5′), 7.16 (1H, s, H-3), 7.36 (1H, d, *J*_trans_ = 15.5 Hz, H-8), 7.64 (2H, d, *J* = 9.0 and 8.7 Hz, H-2′,6′ ), 7.88 (1H, d, *J*_trans_ = 15.5 Hz, H-7), 8.30 (1H, s, H-6), 12.97 (1H, s, -OH); ^13^C-NMR (DMSO-*d*_6_) δ (ppm) 55.5, 84.1, 114.6, 116.5, 119.6, 120.8, 127.0, 131.0, 140.2, 145.5, 146.9, 162.5, 163.8, 191.8; HRMS (ES): MH^+^ found 414.9586. C_16_H_13_O_3_^35^ClI^+^ requires 414.9598.

#### (*E*)-1-(4-Bromo-2-hydroxy-5-iodophenyl)-3-phenylpropenone (**2i**)

Solid (4.92 g, 78%), mp. 145–146 °C; ATR γ_max_ (cm^−1^) 484, 624, 733, 819, 975, 1190, 1323, 1555, 1638, 3025; ^1^H-NMR (DMSO-*d*_6_) δ (ppm) 7.36 (1H, s, H-3), 7.45 (3H, m, Ar), 7.50 (1H, d, *J*_trans_ = 15.0 Hz, H-8), 7.65 (2H, d, *J* = 7.0 Hz, H-2′,6′), 7.93 (1H, d, *J*_trans_ = 15.0 Hz, H-7), 8.28 (1H, s, H-6), 12.74 (1H, s, -OH); ^13^C-NMR (DMSO-*d*_6_) δ (ppm) 87.2, 119.1, 121.0, 123.2, 129.0, 129.1, 131.4, 134.2, 137.5, 140.0, 147.0, 163.3, 192.1; HRMS (ES): MH^+^ found 426.8840. C_15_H_11_O_2_^79^BrI^+^ requires 426.8831.

#### (*E*)-1-(4-Bromo-2-hydroxy-5-iodophenyl)-3-(4-fluorophenyl)propenone **(2j)**

Solid (5.45 g, 83%), mp. 169–170 °C; ATR γ_max_ (cm^−1^) 462, 489, 505, 824, 1185, 1198, 1508, 1556, 1638, 3075; ^1^H-NMR (DMSO-*d*_6_) δ (ppm) 7.14 (2H, t, *J* = 7.8 Hz, H-3′,5′), 7.37 (1H, s, H-3), 7.41 (1H, d, *J*_trans_ = 15.0 Hz, H-8), 7.68 (2H, d, *J* = 7.8 Hz, H-2′,6′), 7.90 (1H, d, *J*_trans_ = 15.0 Hz, H-7), 8.26 (1H, s, H-6), 12.70 (1H, s, -OH); ^13^C-NMR (DMSO-*d*_6_) δ (ppm) 87.2, 116.3 (d, ^2^*J_CF_* = 21.8 Hz), 118.8 (d, ^4^*J*_CF_ = 2.75 Hz), 120.9, 123.2, 130.5, 130.9 (d, ^3^*J*_CF_ = 9.5 Hz), 137.6, 140.0, 145.6, 163.3, 164.6 (d, ^1^*J*_CF_ = 252 Hz), 191.9; HRMS (ES): MH^+^ found 444.8745. C_15_H_10_O_2_F^79^BrI^+^ requires 444.8736.

#### (*E*)-1-(4-Bromo-2-hydroxy-5-iodophenyl)-3-(4-chlorophenyl)propenone (**2k**)

Solid (5.41 g, 80%), mp. 185–187 °C; ATR γ_max_ (cm^−1^) 416, 501, 794, 817, 1024, 1340, 1489, 1552, 1636, 3074; ^1^H-NMR (DMSO-*d*_6_) δ (ppm) 7.37 (1H, s, H-3) 7.42 (2H, d, *J* = 7.5 Hz, H-3′,5′), 7.53 (1H, d, *J*_trans_ = 15.5 Hz, H-8), 7.61 (2H, d, *J* = 8.0 Hz, H-2′,6′ ), 7.88 (1H, d, *J*_trans_ = 15.5 Hz, H-7), 8.26 (1H, s, H-6), 12.70 (1H, s, -OH); ^13^C-NMR (DMSO-*d*_6_) δ (ppm) 87.3, 119.5, 120.9, 123.2, 129.4, 130.0, 132.6, 137.5, 137.7, 140.0, 145.4, 163.3, 191.8; HRMS (ES): MH^+^ found 460.8444. C_15_H_11_O_2_^35^Cl^79^BrI^+^ requires 460.8441.

#### (*E*)-1-(4-Bromo-2-hydroxy-5-iodophenyl)-3-(4-methoxyphenyl)propenone (**2l**)

Solid (5.06 g, 75%), mp. 150–151 °C; ATR γ_max_ (cm^−1^) 489, 559, 624, 825, 1021, 1169, 1507, 1546, 1623, 2919; ^1^H-NMR (DMSO-*d*_6_) δ (ppm) 3.87 (3H. s, -OCH_3_), 6.95 (2H, d, *J* = 8.5 Hz, H-3′,5′), 7.35 (1H, s, H-3), 7.36 (1H, d, *J*_trans_ = 15.5 Hz, H-8), 7.63 (2H, d, *J* = 8.0 Hz, H-2′,6′), 7.91 (1H, d, *J*_trans_ = 15.5 Hz, H-7), 8.26 (1H, s, H-6), 12.90 (1H, s, -OH); ^13^C-NMR (DMSO-*d*_6_) δ (ppm) 55.5, 87.1, 114.6, 116.5, 121.1, 123.1, 127.0, 131.0, 137.1, 140.0, 146.9, 162.5, 163.3, 191.9; HRMS (ES): MH^+^ found 458.9099. C_16_H_13_O_3_^79^BrI^+^ requires 458.9093.

#### (*E*)-1-(2-Hydroxy-5-iodo-4-methoxyphenyl)-3-phenylprop-2-en-1-one (**2m**)

Solid (4.68 g, 72%), mp. 141–143 °C; ATR γ_max_ (cm^−1^) 559, 664,836, 1176, 1214, 1287, 1558, 1603, 1626; ^1^H-NMR (DMSO-*d*_6_) δ (ppm) 3.89 (3H. s, -OCH_3_), 6.42 (1H, s, H-3), 7.43 (2H, d, *J* = 7.5 Hz, H-3′,5′), 7.50 (1H, d, *J*_trans_ = 15.5 Hz, H-8), 7.65 (2H, d, *J* = 7.5 Hz, H-2′,6′ ), 7.88 (1H, d, *J*_trans_ = 15.5 Hz, H-7), 8.22 (1H, s, H-6), 13.42 (1H, s, -OH); ^13^C-NMR (DMSO-*d*_6_) δ (ppm) 56.7, 73.4, 100.2, 116.1, 119.6, 120.0, 128.8, 129.0, 131.0, 134.5, 140.0, 145.3, 163.8, 166.7, 191.0; HRMS (ES): MH^+^ found 380.9984. C_16_H_13_O_3_I^+^ requires 380.9988.

#### (*E*)-3-(4-Fluorophenyl)-1-(2-hydroxy-5-iodo-4-methoxyphenyl)prop-2-en-1-one (**2n**)

Solid (5.31 g, 78%), mp. 197–199 °C; ATR γ_max_ (cm^−1^) 507, 534, 831, 1159, 1208, 1356, 1597, 1632; ^1^H-NMR (DMSO-*d*_6_) δ (ppm) 3.93 (3H. s, -OCH_3_), 6.46 (1H, s, H-3), 7.14 (2H, t, *J* = 8.7 Hz, H-3′,5′), 7.43 (1H, d, *J*_trans_ = 15.5 Hz, H-8), 7.67 (2H, d, *J* = 8.7 Hz, H-2′,6′), 7.86 (1H, d, *J*_trans_ = 15.5 Hz, H-7), 8.23 (1H, s, H-6), 12.37 (1H, s, -OH); ^13^C-NMR (DMSO-*d*_6_) δ (ppm) 56.8, 73.4, 100.3, 116.3 (d, ^2^*J*_CF_ = 21.8 Hz), 119.3 (d, ^4^*J*_CF_ = 2.3 Hz), 130.7 (d, ^3^*J*_CF_ = 8.0 Hz), 140.0, 144.0, 163.9, 164.3 (d, ^1^*J*_CF_ = 252.0 Hz), 166.8, 190.8; HRMS (ES): MH^+^ found 398.9878. C_16_H_12_O_3_FI^+^ requires 398.9893.

#### (*E*)-3-(4-Chlorophenyl)-1-(2-hydroxy-5-iodo-4-methoxyphenyl)prop-2-en-1-one (**2o**)

Solid (5.68 g, 80%), mp. 200–201 °C; ATR γ_max_ (cm^−1^) 430, 565, 798, 829, 1044, 1213, 1560, 1632; ^1^H-NMR (DMSO-*d*_6_) δ (ppm) 3.93 (3H. s, -OCH_3_), 6.46 (1H, s, H-3), 7.42 (2H, d, *J* = 8.1 Hz, H-3′,5′), 7.48 (1H, d, *J*_trans_ = 15.5 Hz, H-8), 7.61 (2H, d, *J* = 8.7 Hz, H-2′,6′ ), 7.85 (1H, d, *J*_trans_ = 15.5 Hz, H-7), 8.23 (1H, s, H-6), 12.34 (1H, s, -OH); ^13^C-NMR (DMSO-*d*_6_) δ (ppm) 56.8, 73.4, 100.3, 116.1, 120.1, 129.3, 130.0, 133.0, 136.9, 140.0, 143.9, 163.9, 166.8, 190.7; HRMS (ES): MH^+^ found 414.9582. C_16_H_12_O_3_^35^ClI^+^ requires 414.9598.

#### (*E*)-1-(2-Hydroxy-5-iodo-4-methoxyphenyl)-3-(4-methoxyphenyl)prop-2-en-1-one (**2p**)

Solid (4.91 g, 70%), mp. 185–187 °C (Lit. [33] 182–184 °C); ATR γ_max_ (cm^−1^) 559, 664, 821, 1176, 1214, 1287, 1510, 1603, 1626; ^1^H-NMR (DMSO-*d*_6_) δ (ppm) 3.87 (3H. s, -OCH_3_), 3.93 (3H. s, -OCH_3_), 6.46 (1H, s, H-5), 6.96 (2H, d, *J* = 8.4 Hz, H-3′,5′), 7.36 (1H, d, *J*_trans_ = 15.3 Hz, H-8), 7.64 (2H, d, *J* = 8.7 Hz, H-2′,6′), 7.88 (1H, d, *J*_trans_ = 15.6 Hz, H-7), 8.25 (1H, s, H-6), 13.53 (1H, s, -OH); ^13^C-NMR (DMSO-*d*_6_) δ (ppm) 55.5, 56.7, 73.2, 100.3, 114.5, 116.3, 117.2, 127.3, 130.6, 139.9, 145.3, 162.1, 163.7, 166.7, 191.0; HRMS (ES): MH^+^ found 411.0098. C_17_H_15_O_4_I^+^ requires 411.0095.

### 3.4. Typical Procedure for the Synthesis of the 7-substituted 2-aryl-3-hydroxy-6-iodochromen-4-ones ***3a–p***

A mixture of **2a** (0.50 g 1.17 mmol) and 3 M KOH (15 mL) in ethanol (20 mL) was stirred at RT for 15 min and then placed in an ice-bath. Hydrogen peroxide (5 mL) was added slowly to the reaction mixture and stirring was continued for 30 min. The ice-bath was removed and the reaction mixture was stirred at RT for 3 h and then poured into a mixture of crushed ice and conc. HCl. The organic phase was extracted with chloroform and the combined organic layers were dried over anhydrous MgSO_4_. The salt was filtered off and the solvent was evaporated on a rotary evaporator under reduced pressure. The residue was purified by column chromatography on silica gel to afford **3** as a yellow solid. Compounds **3a**–**p** were prepared in this fashion.

#### 7-Flu.oro-3-hydroxy-6-iodo-2-phenyl-4*H*-chromen-4-one (**3a**)

Solid (0.42 g, 80%), mp. 275–276 °C; ATR γ_max_ (cm^−1^) 499, 577, 654, 782, 841, 1105, 1204, 1369, 1442, 1590, 1609, 3075; ^1^H-NMR (DMSO-*d*_6_) δ (ppm) 7.52 (3H, m, H-4′ and H-3′,5′), 7.78 (1H, d, *J* = 8.7 Hz, H-8), 8.16 (2H, d, *J* = 6.3 Hz, H-2′,6′), 8.44 (1H, d, *J* = 7.2 Hz, H-5), 9.83 (1H, s, -OH); ^13^C-NMR (DMSO-*d*_6_) δ (ppm) 79.6 (d, ^2^*J*_CF_ = 27.5 Hz), 106.0 (d, ^2^*J*_CF_ = 28.5 Hz), 120.7, 128.0, 129.0, 130.5 (d, ^4^*J*_CF_ = 2.3 Hz), 131.3, 136.0 (d, ^3^*J*_CF_ = 4.5 Hz), 139.6, 146.4, 155.7 (d, ^3^*J*_CF_ = 13.7 Hz), 163.7 (d, ^1^*J*_CF_ = 247.3 Hz), 171.7; HRMS (ES): MH^+^ found 382.9575. C_15_H_9_O_3_FI^+^ requires 382.9580.

#### 7-Flu.oro-2-(4-fluorophenyl)-3-hydroxy-6-iodo-4*H*-chromen-4-one (**3b**)

Solid (0.38 g, 73%), mp. 330–331 °C; ATR γ_max_ (cm^−1^) 502, 553, 650, 836, 1158, 1214, 1392, 1496, 1557, 1585, 2361, 2986, 3073; ^1^H-NMR (DMSO-*d*_6_) δ (ppm) 7.10 (2H, d, *J* = 8.7 Hz, H-3′,5′), 7.80 (1H, d, *J* = 8.1 Hz, H-8), 8.14 (2H, d, *J* = 9.0 Hz, H-2′,6′), 8.44 (1H, d, *J* = 7.0 Hz, H-5), 9.70 (1H, s, -OH); ^13^C-NMR (DMSO-*d*_6_) δ (ppm) 79.4, 105.5 (d, ^2^*J*_CF_ = 26.2 Hz), 115.3 (d, ^2^*J*_CF_ = 24.0 Hz), 116.4 (d, ^2^*J*_CF_ = 21.75 Hz), 122.3, 129.5 (d, ^3^*J*_CF_ = 8.5 Hz), 131.6 (d, ^4^*J*_CF_ = 2.9 Hz), 132.2 (d, ^3^*J*_CF_ = 8.5 Hz), 141.5 (d, ^4^*J*_CF_ = 2.8 Hz), 144.5, 164.2 (d, ^1^*J*_CF_ = 248.4 Hz), 164.6, 165.3 (d, ^1^*J*_CF_ = 249.4 Hz); HRMS (ES): MH^+^ found 401.1101. C_15_H_8_O_3_F_2_I^+^ requires 401.1223.

#### 2-(4-C.hlorophenyl)-7-fluoro-3-hydroxy-6-iodo-4*H*-chromen-4-one (**3c**)

Solid (0.41 g, 79%), mp. 225–226 °C; ATR γ_max_ (cm^−1^) 469, 634, 654, 831, 1011, 1089, 1218, 1391, 1450, 1551, 1586, 2361, 3072, 3095; ^1^H-NMR (DMSO-*d*_6_) δ (ppm) 7.12 (2H, d, *J* = 7.5 Hz, H-3′,5′), 7.72 (1H, d, *J*= 8.7 Hz, H-3), 7.94 (2H, d, *J* = 8.1 Hz, H-2′,6′), 8.25 (1H, d, *J* = 8.0 Hz, H-5); ^13^C-NMR (DMSO-*d*_6_) δ (ppm) 79.5 (d, ^2^*J*_CF_ = 27.4 Hz), 106.0 (d, ^2^*J*_CF_ = 29.8 Hz), 114.5 (2×C), 120.8, 123.6, 129.8, 135.8, 138.7, 146.7, 155.2 (d, ^3^*J*_CF_ = 13.7 Hz), 161.0, 163.5 (d, ^1^*J*_CF_ = 247.4 Hz), 171.4; HRMS (ES): MH^+^ found 416.9173. C_15_H_8_O_3_F^35^ClI^+^ requires 416.9191.

#### 7-Flu.oro-3-hydroxy-6-iodo-2-(4-methoxyphenyl)-4*H*-chromen-4-one (**3d**)

Solid (0.43 g, 84 %), mp. 261–262 °C; ATR γ_max_ (cm^−1^) 492, 568, 659, 1034, 1177, 1205, 1255, 1388, 1450, 1558, 1595, 2360, 2837, 2935, 3092; ^1^H-NMR (DMSO-*d*_6_) δ (ppm) 3.76 (3H, s, -OCH_3_), 7.02 (2H, d, *J* = 8.1 Hz, H-3′,5′), 7.71 (1H, d, *J* = 8.1 Hz, H-8), 8.12 (2H, d, *J* = 8.7 Hz, H-2′,6′), 8.35 (1H, d, *J* = 7.2 Hz, H-5), 9.61 (1H, s, -OH); ^13^C-NMR (DMSO-*d*_6_) δ (ppm) 55.8, 79.3 (d, ^2^*J*_CF_ = 27.5 Hz), 106.0 (d, ^2^*J*_CF_ = 28.6 Hz), 114.4, 120.7, 123.6 (d, ^3^*J*_CF_ = 13.7 Hz), 128.0, 129.7, 135.8, 138.5 (d, ^3^*J*_CF_ = 11.4 Hz), 146.7, 155.5 (d, ^3^*J*_CF_ = 13.7 Hz), 160.9, 163.4 (d, ^1^*J*_CF_ = 247.3 Hz), 171.3; HRMS (ES): MH^+^ found 412.9690. C_16_H_11_O_4_FI^+^ requires 412.9686.

#### 7-Chl.oro-3-hydroxy-6-iodo-2-phenyl-4*H*-chromen-4-one (**3e**)

Solid (0.39 g, 75%), mp. 303–304 °C; ATR γ_max_ (cm^−1^) 468, 486, 505, 686, 765, 948, 1012, 1207, 1432, 1549, 1592, 1605, 2323, 2912, 3000, 3054; ^1^H-NMR (DMSO-*d*_6_) δ (ppm) 7.23 (3H, d, *J* = 6.9 Hz, H-4′ and H-3′,5′), 7.93 (2H, d, *J* = 5.4 Hz, H-2′,6′), 8.02 (1H, s, H-8), 8.21 (1H, s, H-5), 9.64 (1H, s, -OH); ^13^C-NMR (DMSO-*d*_6_) δ (ppm) 91.3, 110.0, 119.2, 112.5, 125.7, 126.9, 128.2, 135.0, 136.6, 139.3, 144.6, 153.7, 177.4; HRMS (ES): MH^+^ found 398.9275, C_15_H_9_O_3_^35^ClI^+^ requires 398.9285.

#### 7-Chl.oro-2-(4-fluorophenyl)-3-hydroxy-6-iodo-4*H*-chromen-4-one (**3f**)

Solid (0.36 g, 70%), mp. 330–331 °C; ATR γ_max_ (cm^−1^) 459, 501, 540, 666, 736, 836, 948, 1014, 1098, 1158, 1222, 1402, 1433, 1560, 1578, 1634, 2911, 3004, 3058; ^1^H-NMR (DMSO-*d*_6_) δ (ppm) 7.42 (2H, t, *J* = 9.0 Hz, H-3′,5′), 8.18 (1H, s, H-8), 8.23 (2H, t, *J* = 6.0 Hz, H-2′,6′), 8.57 (1H, s, H-5), 10.02 (1H, s, -OH); ^13^C-NMR (DMSO-*d*_6_) δ (ppm) 94.1, 116.1 (d, ^2^*J*_CF_ = 21.8 Hz), 119.9, 122.2,, 127.9 (d, ^4^*J*_CF_ = 2.75 Hz), 130.7 (d, ^3^*J*_CF_ = 8.5 Hz), 136.2, 140.0, 142.3, 145.5, 154.7, 163.2 (d, ^1^*J*_CF_ = 248.3 Hz), 171.7; HRMS (ES): MH^+^ found 416.9181. C_15_H_8_O_3_F^35^ClI^+^ requires 416.9191.

#### 7-Chl.oro-2-(4-chlorophenyl)-3-hydroxy-6-iodo-4*H*-chromen-4-one (**3g**)

Solid (0.35 g, 67%), mp. 247–248 °C; ATR γ_max_ (cm^−1^) 484, 509, 636, 806, 949, 1013, 1207, 1434, 1449, 1550, 1593, 1635, 2911, 3025, 3060; ^1^H-NMR (DMSO-*d*_6_) δ (ppm) 6.67 (2H, d, *J* = 8.1 Hz, H-3′,5′), 7.20 (1H, s, H-8), 7.34 (2H, d, *J* = 7.5 Hz, H-2′,6′ ), 7.55 (1H, s, H-5), 10.09; ^13^C-NMR (DMSO-*d*_6_) δ (ppm) 94.6, 120.0, 122.2, 129.1, 129.8, 130.4, 135.2, 136.2, 140.2, 142.4, 145.0, 154.7, 171.8; HRMS (ES): MH^+^ found 432.8890. C_15_H_8_O_3_^35^Cl_2_I^+^ requires 432.8895.

#### 7-Chl.oro-3-hydroxy-6-iodo-2-(4-methoxyphenyl)-4*H*-chromen-4-one (**3h**)

Solid (0.32 g, 62%), mp. 252–253 °C; ATR γ_max_ (cm^−1^) 506, 635, 732, 767, 806, 949, 1013, 1114, 1259, 1450, 1594, 1610, 2339, 2913, 3005, 3027, 3059; ^1^H-NMR (DMSO-*d*_6_) δ (ppm) 3.32 (1H, s, -OCH_3_), 6.57 (2H, d, *J* = 6.0 Hz, H-3′,5′), 7.60 (1H, s, H-8), 7.67 (2H, d, *J* = 6.3 Hz, H-2′,6′ ), 8.00 (1H, s, 5-H); ^13^C-NMR (DMSO-*d*_6_) δ (ppm) 55.6, 94.2, 114.4, 119.7, 122.2, 123.7, 130.0, 131.3, 136.0, 141.8, 146.5, 154.4, 160.9, 171.5; HRMS (ES): MH^+^ found 428.9379. C_16_H_11_O_4_^35^ClI^+^ requires 428.9391.

#### 7-Bro.mo-3-hydroxy-6-iodo-2-phenyl-4*H*-chromen-4-one (**3i**)

Solid (0.40 g, 77%), mp. 190–191 °C; ATR γ_max_ (cm^−1^) 468, 685, 765, 1126, 1205, 1372, 1429, 1549, 1607, 1682, 2149, 3079, 3278; ^1^H-NMR (DMSO-*d*_6_) δ (ppm) 7.23 (4H, d, *J* = 7.8 Hz, H-8, H-4′ and H-3′,5′), 7.91 (2H, d, *J* = 9.0 Hz, H-2′,6′), 8.18 (1H, s, H-5), 9.58 (1H, s, -OH); ^13^C-NMR (DMSO-*d*_6_) δ (ppm) 97.4, 122.4, 123.1, 128.1, 128.9, 130.5, 131.3, 134.2, 135.8, 139.9, 146.0, 154.3, 171.8; HRMS (ES): MH^+^ found 444.0754. C_15_H_9_O_3_^79^BrI^+^ requires 444.0777.

#### 7-Bro.mo-2-(4-fluorophenyl)-3-hydroxy-6-iodo-4*H*-chromen-4-one (**3j**)

Solid (0.37 g, 72%), mp. 180–181 °C; ATR γ_max_ (cm^−1^) 436, 500, 626, 697, 788, 861, 1097, 1201, 1240, 1431, 1594, 1666, 2927, 3076; ^1^H-NMR (DMSO-*d*_6_) δ (ppm) 7.42 (2H, t, *J* = 8.7 Hz, H-3′,5′), 8.26 (2H, d, *J* = 6.0 Hz, H-2′,6′), 8.32 (1H, s, H-8), 8.48 (1H, s, H-5), 10.02 (1H, s, -OH); ^13^C-NMR (DMSO-*d*_6_) δ (ppm) 94.5, 116.1 (d, ^2^*J*_CF_ = 21.75 Hz), 119.9, 122.2, 127.9 (d, ^4^*J*_CF_ = 2.3 Hz), 130.6 (d, ^3^*J*_CF_ = 9.15 Hz), 136.2, 139.7, 142.3, 145.5, 154.7, 163.2 (d, ^1^*J*_CF_ = 248.5 Hz), 171.8; HRMS (ES): MH^+^ found 460.8680. C_15_H_8_O_3_F^79^BrI^+^ requires 460.8696.

#### 7-Bro.mo-2-(4-chlorophenyl)-3-hydroxy-6-iodo-4*H*-chromen-4-one (**3k**)

Solid (0.42 g, 81%), mp. 282–283 °C; ATR γ_max_ (cm^−1^) 497, 590, 635, 834, 1093, 1110, 1206, 1430, 1449, 1548, 1590, 1601, 1653, 3082, 3250; ^1^H-NMR (DMSO-*d*_6_) δ (ppm) 7.62 (2H, d, *J* = 8.0 Hz, H-3′, 5′), 8.15 (1H, s, H-8), 8.20 (2H, d, *J* = 7.0 Hz, H-2′,6′), 8.49 (1H, s, H-5), 10.09 (1H, s, -OH); ^13^C-NMR (DMSO-*d*_6_) δ (ppm) 97.9, 122.7, 123.5, 129.4, 130.1, 130.5, 134.7, 135.5, 136.2, 140.5, 145.2, 154.6, 172.2; HRMS (ES): MH^+^ found 476.8386. C_15_H_8_O_3_^35^Cl^79^BrI^+^ requires 476.8390.

#### 7-Bro.mo-3-hydroxy-6-iodo-2-(4-methoxyphenyl)-4*H*-chromen-4-one (**3l**)

Solid (0.38 g, 74%), mp. 201–202 °C; ATR γ_max_ (cm^−1^) 435, 499, 625, 696, 787, 901, 1096, 1200, 1311, 1430, 1456, 1590, 1661, 2867, 2924, 3046; ^1^H-NMR (DMSO-*d*_6_) δ (ppm) 3.40 (3H, s, -OCH_3_), 6.64 (2H, d, *J* = 6.3 Hz, H-3′,5′), 6.91 (1H, s, H-8), 7.72 (2H, d, *J* = 7.5 Hz, H-2′,6′ ), 8.00 (1H, s, H-5), 9.28 (1H, s, -OH); ^13^C-NMR (DMSO-*d*_6_) δ (ppm) 55.8, 97.4, 114.5, 23.1, 130.0, 134.0, 135.8, 139.0, 140.9, 146.6, 154.3, 161.1, 161.3, 171.5; HRMS (ES): MH^+^ found 472.8863. C_16_H_11_O_4_^79^BrI^+^ requires 472.8885.

#### 3-Hyd.roxy-6-iodo-7-methoxy-2-phenyl-4*H*-chromen-4-one (**3m**)

Solid (0.39 g, 75%), mp. 233–234 °C; ATR γ_max_ (cm^−1^) 459, 659, 829, 1034, 1175, 1213, 1263, 1451, 1599, 2359, 2838, 3095; ^1^H-NMR (DMSO-*d*_6_) δ (ppm) 3.93 (3H, s, -OCH_3_), 7.20 (1H, s, H-5), 7.34 (3H, t, *J* = 7.5 Hz, H-3′,5′ and h-5′), 8.30 (1H, s, H-8), 8.53 (2H, d, *J* = 7.5 Hz, H-2′,6′); ^13^C-NMR (DMSO-*d*_6_) δ (ppm) 57.5, 81.7, 100.2, 117.9, 125.4, 126.5, 127.3, 128.2, 128.5, 135.2, 135.4, 143.7, 155.7, 160.0; HRMS (ES): MH^+^ found 394.9775. C_16_H_12_O_4_I^+^ requires 394.9780.

#### 2-(4-F.luorophenyl)-3-hydroxy-6-iodo-7-methoxy-4*H*-chromen-4-one (**3n**)

Solid (0.42 g, 82%), mp. > 350 °C; ATR γ_max_ (cm^−1^) 469, 632, 667, 825, 1009, 1038, 1087, 1207, 1263, 1443, 1477, 1548, 1588, 2360, 2936, 2966, 3095; ^1^H-NMR (DMSO-*d*_6_) δ (ppm) 3.92 (3H, s, -OCH_3_), 7.14 (1H, s, H-5), 7.31 (2H, d, *J* = 7.2 Hz, H-3′,5′), 8.30 (1H, s, H-8), 8.62 (2H, d, *J* = 7.5 Hz, H-2′,6′); ^13^C-NMR (DMSO-*d*_6_) δ (ppm) 55.8, 97.4, 114.5, 123.1, 130.0, 134.0, 135.8, 139.0, 140.9, 146.6, 154.3, 161.1, 161.3, 171.5; HRMS (ES): MH^+^ found 413.15766. C_16_H_11_O_4_FI^+^ requires 413.1574.

#### 2-(4-C.hlorophenyl)-3-hydroxy-6-iodo-7-methoxy-4*H*-chromen-4-one (**3o**)

Solid (0.38 g, 74%), mp 258–259 °C; ATR γ_max_ (cm^−1^) 484, 635, 662, 831, 1089, 1172, 1212, 1262, 1446, 1480, 1552, 1598, 2360, 3105, 3199; ^1^H-NMR (DMSO-*d*_6_) δ (ppm) 3.95 (3H, s, -OCH_3_), 7.28 (1H, s, H-8),7.52 (2H, d, *J* = 6.0 Hz, H-3′,5′), 8.28 (2H, d, *J* = 6.0 Hz, H-2′,6′), 8.33 (1H, s, H-5), 9.86 (1H, s, -OH); ^13^C-NMR (DMSO-*d*_6_) δ (ppm) 57.8, 83.8, 100.4, 114.3, 117.2, 127.8, 128.8, 129.0, 131.1, 134.0, 135.1, 143.7, 156.5, 161.5; MH^+^ found 428.9385, C_16_H_11_O_4_^35^ClI^+^. requires 428.9391.

#### 3-Hyd.roxy-6-iodo-7-methoxy-2-(4-methoxyphenyl)-4*H*-chromen-4-one (**3p**)

Solid (0.39 g, 76%), mp. 230–231 °C; ATR γ_max_ (cm^−1^) 498, 567, 661, 787, 1041, 1206, 1251, 1434, 1603, 1651, 2495, 2836, 2937, 3039; ^1^H-NMR (DMSO-*d*_6_) δ (ppm) 3.83 (3H, s, -OCH_3_), 3.97 (3H, s, -OCH_3_), 7.10 (2H, d, *J* = 9.0 Hz, H-3′,5′), 7.35 (1H, s, H-5), 8.16 (2H, d, *J* = 8.7 Hz, H-2′,6′), 8.34 (1H, s, H-8), 8.52 (1H, s, -OH); ^13^C-NMR (DMSO-*d*_6_) δ (ppm) 55.8, 57.8, 84.0, 100.5, 114.5, 117.4, 123.9, 129.6, 135.0, 138.4, 145.8, 156.6, 160.8, 161.6, 171.4; HRMS (ES): MH^+^ found 424.9887. C_17_H_14_O_5_I^+^, requires 424.9886.

### 3.5. In Vitro Cholinesterase (AChE and BChE) Inhibition Assays

Cholinesterase activities were assayed at 25 °C by the Ellman’s method [34], which involves following the hydrolysis of acetylthiocholine iodide (ACh) or butyrylthiocholine iodide (BCh) by monitoring formation of 5-thio-2-nitrobenzoate from 5-5′-dithiobis(2-nitrobenzoic acid) spectrophotometrically at 412 nm. The solution of the test compound **2** or **3** was prepared in DMSO (20 mL) and diluted in 50 mM tris buffer (pH 7.7) to obtain the final concentrations (10–100 µM) for assay. All the experiments were performed in triplicate in a 96-well plate reader at controlled temperature (37 °C). 70 µL of tris buffer (C_4_H_11_NO_3_, 50 mM, pH 7.7), 9.0 µL of the test compound solution and 1.0 µL of the respective enzyme were added sequentially to each well. The assay mixture was preincubated for 20 min at room temperature (RT), and then 10 µL of 5,5′-dithiobis-(2-nitrobenzoic acid) (DTNB, 3 mM in buffer) and 10 µL of ACh (5 mM in buffer) were added to each well. The absorbances were determined spectrometrically at 412 nm on a Varioskan flash spectrophotometer (Thermo Scientific, Waltham, MA, USA). The IC_50_ and standard deviation values were determined graphically using Graph Pad Prism.

### 3.6. In Vitro BACE-1 Inhibitory Assays

The inhibitory properties of chalcones (**2h**, **2j**, **2n** and **2p**) or flavonols (**3b**, **3c**, **3l** and **3p**) on BACE-1 were evaluated by a fluorescence resonance energy transfer (FRET) assay (Pan Vera) with a recombinant baculovirus-expressed BACE-1 and a specific substrate (Rh-EVNLDAEFK-Quencher) according to manufacturer instructions. A mixture of human recombinant BACE-1 (1.0 U/mL), the substrate (75 µM in 50 mM ammonium bicarbonate), and test compound dissolved in an assay buffer (50 mM sodium acetate, pH 4.5), was incubated for 60 min at 25 °C in a 96-well plate. The increase in fluorescence intensity produced by substrate hydrolysis was observed on a fluorescence microplate reader with an excitation wavelength of 545 and emission wavelength 590 nm. The inhibition ratio was calculated using the following equation:

Inhibition (%) = [1 − (S − S_0_)/(C − C_0_)] × 100 (1)

where C is the fluorescence of control (enzyme, assay buffer, and substrate) after 60 min of incubation, C_0_ is the fluorescence of control at time 0, S is the fluorescence of tested samples (enzyme, sample solution, and substrate) after 60 min of incubation, and S_0_ the fluorescence of the tested samples at time 0.

### 3.7. Kinetic Studies against ChEs and BACE-1

#### 3.7.1. Kinetic Evaluation of **2h**, **3b** and **3p** against ChEs

The experiment was performed using different inhibitor (**2h**, **3b** or **3p**) concentrations (0, 2.5, 3.5 and 5 µM) and substrate (acetylthiocholine iodide (ACh) or butyrylthiocholine iodide (BCh) concentrations (0.1, 0.5, 2.5 and 5 µM). The reaction was carried out in triplicate and the absorbences were measured at 412 nm after every 1 s. A linear graph of the absorbences against time were plotted to obtain the velocity. Finally, the lineweaver burk plot (reciprocal of the velocity versus reciprocal of the subtrate concentrations) was plotted. as well as the Dixon plot (reciprocal of the velocity versus inhibitor concentration). The inhibition constant *K_i_*, was determined by calculating the straight line equations and identifying the point at which the lines would intersect.

#### 3.7.2. Kinetic Evaluation of **2j** and **3l** against BACE-1

The kinetics assays were conducted according to the manufacturer’s instructions similarly to the inhibitory assay except that change in fluorescence over time was monitored at 590 nm without the addition of the stop solution. Each substrate (0.15, 0.30, 0.45 µM) and compound (0, 4, 8 and 16 µM) concentration pair were prepared in triplicate and analyzed. The Lineweaver Burk and Dixon plots were plotted as described in Section 3.7.1.

### 3.8. Molecular Docking

Discovery studio and specifically the CDOCKER module was used to investigate interactions of compounds with AChE, BChE and BACE-1. The protein structures exported from the Protein Data Bank (PDB) for AChE were, 1E66 and 1GQR, and for BChE was 1P0I. The PDB structure used for docking of compounds **2h**, **2p**, **3b** and **3p** to BACE-1 was 4D8C. The compounds were drawn in discovery studio and docked into AChE, BChE or BACE-1 binding sites. The binding sites used to dock compounds represented co-crystalized ligand or substrate location and all other parameters were maintained as defaults for the docking. Prior to docking both protein and compound structures were prepared using the prepare protein and prepare ligand protocols in discovery studio, respectively. Docking resulted in at least ten poses per compound docked to each protein structure, the best scoring pose without unfavorable interactions was selected and represented as 2D plots using discovery studio.

## 4. Conclusions

The chalcone derivatives **2h**, **2j**, **2n** and **2p** exhibited moderate inhibitory effect against AChE activity when compared to donepezil, but increased inhibitory effect and selectivity against BChE. These compounds were also found to exhibit moderate to significant inhibitory effect against BACE-1. Compounds **2h** and **2j** in particular have potential to act as dual inhibitors against BChE and BACE-1 activities. The flavonol derivatives **3b** and **3c** appear to be more selective against AChE activity and exhibit significantly reduced inhibitory effect against BChE and moderate effect against BACE-1 activity. The flavonol derivatives **3k**, **3l** and **3p**, on the other hand, tend to be more selective against BChE activity with modest inhibitory effect against AChE. Moderate and significant inhibitory effect were observed for the flavonols **3p** and **3l** against BACE-1 activity, respectively. Molecular docking studies into AChE, BChE and BACE-1 binding sites suggested electrostatic and hydrophobic interactions as the key factors that stabilize the enzyme-ligand complexes. The results of the kinetic studies of **2j** and **3l** on BACE-1 indicate a mixed mode of inhibition. The *ortho*-hydroxy-*trans-*α,β-unsaturated carbonyl framework makes the chalcones prepared in this investigation potential metal chelating pharmacophores that could widen their spectrum of biological activities. Selectivity against BChE has been suggested to be crucial with relation to inflammation, oxidative stress, and lipid metabolism [35]. Some of these chalcones and flavonol derivatives represent suitable candidates for further studies of biological activity as anti-inflammatory and/or antioxidant agents.

## Figures and Tables

**Table 1:** Substitution pattern for compounds **2a**–**p** and **3a**–**p**.

**Ar**	**Designation of *X* for 2a–p**			
C_6_H_5_-	F (**2a**)	Cl (**2e**)	Br (**2i**)	OCH_3_ (**2m**)
4-FC_6_H_4_-	F (**2b**)	Cl (**2f**)	Br (**2j**)	OCH_3_ (**2n**)
4-ClC_6_H_4_-	F (**2c**)	Cl (**2g**)	Br (**2k**)	OCH_3_ (**2o**)
4-MeOC_6_H_4_-	F (**2d**)	Cl (**2h**)	Br (**2l**)	OCH_3_ (**2p**)
**Ar**	**Designation of X for 3a–p**			
C_6_H_5_-	F (**3a**)	Cl (**3e**)	Br (**3i**)	OCH_3_ (**3m**)
4-FC_6_H_4_-	F (**3b**)	Cl (**3f**)	Br (**3j**)	OCH_3_ (**3n**)
4-ClC_6_H_4_-	F (**3c**)	Cl (**3g**)	Br (**3k**)	OCH_3_ (**3o**)
4-MeOC_6_H_4_-	F (**3d**)	Cl (**3h**)	Br (**3l**)	OCH_3_ (**3p**)

**Table 2:** AChE and BChE inhibitory activities of flavonols **2a**–**p**.

Compound	IC_50_ (µM)		SI	
	AChE	BChE	BChE/AChE	AChE/BChE
**2a**	38.78 ± 0.01	28.06 ± 0.10	0.72	1.38
**2b**	16.60 ± 0.03	19.73 ± 0.05	1.19	0.84
**2c**	10.50 ± 0.08	14.58 ± 0.14	1.39	0.72
**2d**	10.95 ± 0.30	8.49 ± 0.05	0.78	1.29
**2e**	10.64 ± 0.07	25.19 ± 0.30	2.37	0.46
**2f**	9.57 ± 0.20	17.86 ± 0.08	1.87	0.54
**2g**	11.00 ± 0.06	>100	-	-
**2h**	11.72 ± 0.07	4.17 ± 0.15	0.36	2.81
**2i**	58.14 ± 0.21	14.11 ± 0.11	0.24	4.12
**2j**	10.98 ± 0.03	5.73 ± 0.05	0.52	1.92
**2k**	11.56 ± 0.05	10.44 ± 0.09	0.90	1.11
**2l**	10.51 ± 0.03	15.25 ± 0.01	1.45	0.69
**2m**	10.01 ± 0.09	9.52 ± 0.03	0.95	1.05
**2n**	9.38 ± 0.07	5.91 ± 0.05	0.63	1.59
**2o**	11.07 ± 0.12	11.63 ± 0.17	1.05	0.95
**2p**	10.96 ± 0.03	4.89 ± 0.02	0.45	2.24
**Donepezil**	4.77 ± 0.06	6.04 ± 0.03	1.27	0.79

The values are given as mean ± standard deviation (SD) in three independent experiments. SI (Selectivity Index): the AChE selectivity index is defined as IC_50_ (BChE)/IC_50_ (AChE) and that of BChE as IC_50_ (AChE)/IC_50_ (BChE) affinity ratios.

**Table 3:** AChE and BChE inhibitory activities of flavonols **3a**–**p**.

Compound	IC_50_ (µM)		SI	
	AChE	BChE	BChE/AChE	AChE/BChE
**3a**	29.71 ± 0.01	27.01 ± 0.06	0.91	1.10
**3b**	3.23 ± 0.02	29.78 ± 0.04	9.22	0.11
**3c**	3.45 ± 0.07	37.51 ± 0.08	10.8	0.09
**3d**	58.18 ± 0.03	29.41 ± 0.04	0.50	1.98
**3e**	47.85 ± 0.04	6.64 ± 0.03	0.14	7.21
**3f**	38.39 ± 0.02	18.17 ± 0.10	0.47	2.11
**3g**	59.10 ± 0.05	7.69 ± 0.01	0.13	7.69
**3h**	14.33 ± 0.03	5.72 ± 0.01	0.40	2.51
**3i**	9.96 ± 0.01	5.76 ± 0.04	0.57	1.73
**3j**	11.16 ± 0.01	27.01 ± 0.07	2.42	0.41
**3k**	29.24 ± 0.03	5.25 ± 0.01	0.18	5.57
**3l**	28.99 ± 0.02	4.88 ± 0.01	0.17	5.94
**3m**	9.25 ± 0.09	10.50 ± 0.02	1.14	0.88
**3n**	6.15 ± 0.01	7.93 ± 0.06	1.29	0.76
**3o**	55.27 ± 0.04	13.71 ± 0.14	0.24	4.03
**3p**	7.19 ± 0.02	3.29 ± 0.03	0.45	2.18
**Donepezil**	4.79 ± 0.05	6.04 ± 0.03	1.26	0.79

The values are given as mean ± standard deviation (SD) in three independent experiments. SI (Selectivity Index): the AChE selectivity index is defined as IC_50_ (BChE)/IC_50_ (AChE) and that of BChE as IC_50_ (AChE)/IC_50_ (BChE) affinity ratios.

**Table 4:** IC_50_ values for the inhibitory effect of **2h**, **2j**, **2n**, **2p**, **3b**, **3c**, **3l**, **3p** against BACE-1 activity.

Compound	BACE-1
**2h**	13.82 ± 0.03
**2j**	4.703 ± 0.06
**2n**	25.07 ± 0.1
**2p**	70.79 ± 0.2
**3b**	32.18 ± 0.15
**3c**	19.69 ± 0.05
**3l**	15.74 ± 0.12
**3p**	22.44 ± 0.07
**Quercetin**	12.66 ± 0.02

## Supplementary Materials

Supplementary materials can be found at http://www.mdpi.com/1422-0067/19/12/4112/s1.
