# Supplementary material for: Synthesis and Evaluation of the 4-Substituted 2-Hydroxy-5-Iodochalcones and Their 7-Substituted 6-Iodoflavonol Derivatives for Inhibitory Effect on Cholinesterases and β-Secretase
Source: Int J Mol Sci. 2018 Dec 18;19(12):4112. doi: 10.3390/ijms19124112 (PMC6321475; doi:10.3390/ijms19124112)
Supplement: Supplementary file 1 [file ijms-19-04112-s001.pdf]

International Journal of
Molecular Sciences

MDPI

Supplementary Material

Synthesis and evaluation of the 4-substituted 2-hydroxy-5-iodochalcones and their 7-substituted 6-iodoflavonol derivatives for inhibitory effect on cholinesterases and β-secretase

M.J. Mphahlele; E.N. Agbo and S. Gildenhuys

---

^{1}H and ^{13}C NMR spectra of the prepared compounds (Figure S1), IC_{50} values of chalcones (**2h**, **2j**, **2n** & **2p**) and flavonols (**3b**, **3c**, **3l** & **3p**) against BACE-1 activity, docking poses of compounds **2a**–**p** and **3a**–**p** against the active site of AChE (Figure S2) and BChE (Figure S3), respectively.

Figure S1. ^{1}H- and ^{13}C-NMR spectra of compounds 1, 2a–p and 3a–p.

Table S1: IC_{50} values for BACE-1 inhibitory effect of 2h, 2j, 2n, 2p, 3b, 3c, 3l, 3p & quercetin

Figure S2: Docking poses of compounds 2a–p and 3a–p against the active site of AChE

Figures S3: Docking poses of compounds 2a–p and 3a–p against the active site of BChE

Figure S4: Docking poses of 2p, 3b and 3p into BACE-1

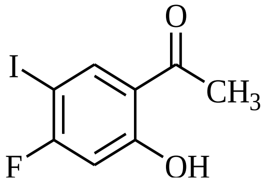

Figure S1. ^{1}H and ^{13}C NMR spectra of compounds **1a–d** , **2a–p** and **3a–p.**

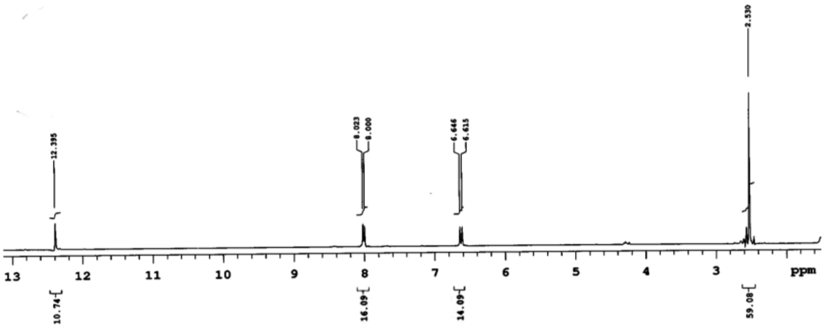

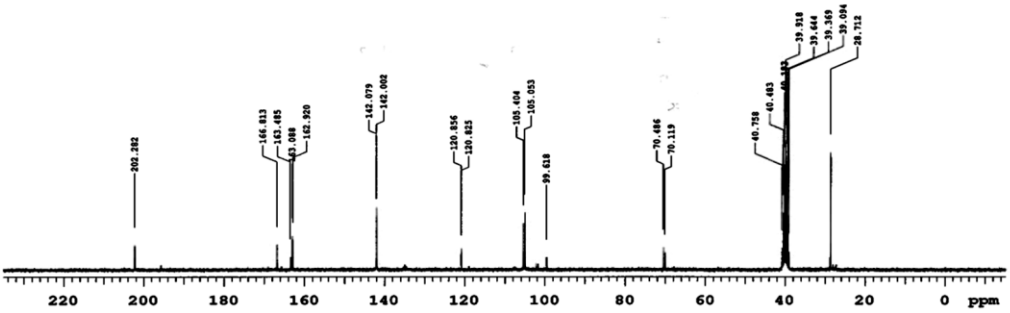

Figure S1.1. ^{1}H- and ^{13}C-NMR spectra of **1a** in $CDCl_3$ at 300 and 75 MHz, respectively.

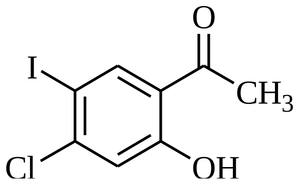

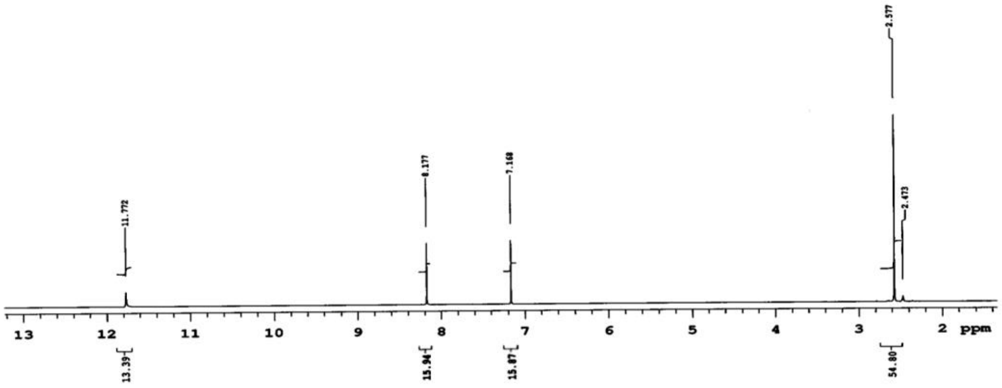

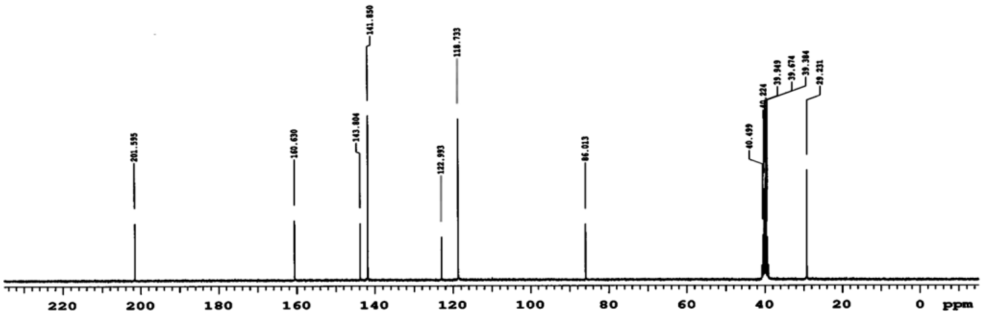

Figure S1.2. ^{1}H- and ^{13}C-NMR spectra of **1b** in $CDCl_3$ at 300 and 75 MHz, respectively.

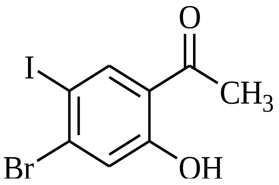

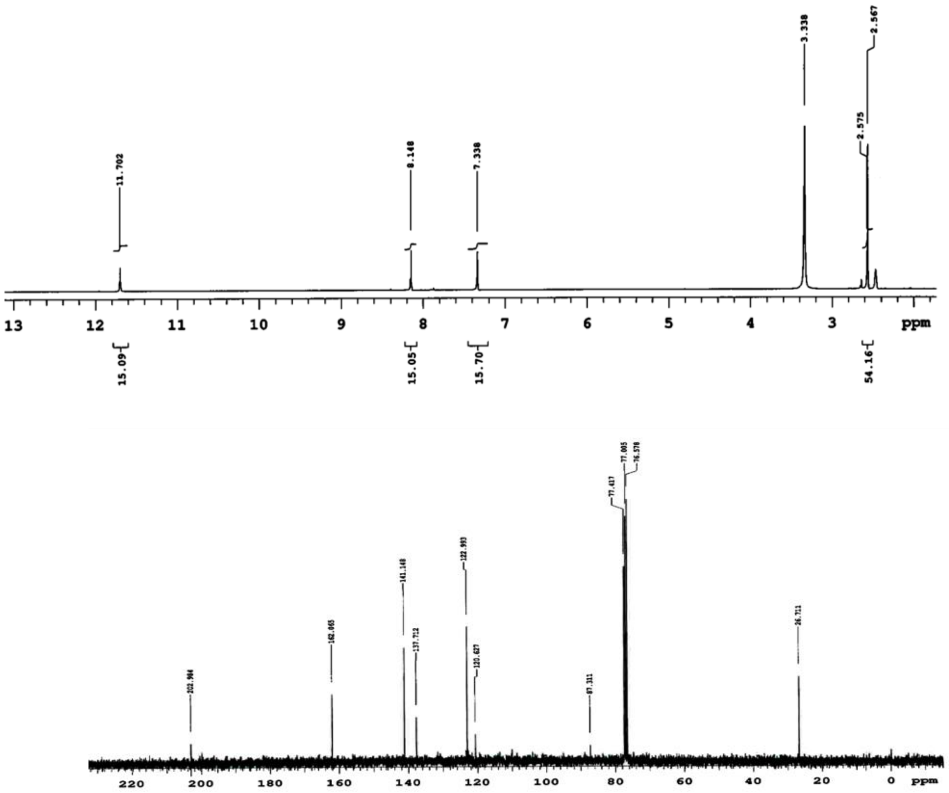

Figure S1.3. ^{1}H- and ^{13}C-NMR spectra of **1c** in $CDCl_3$ at 300 and 75 MHz, respectively.

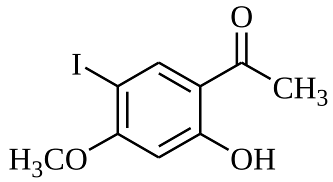

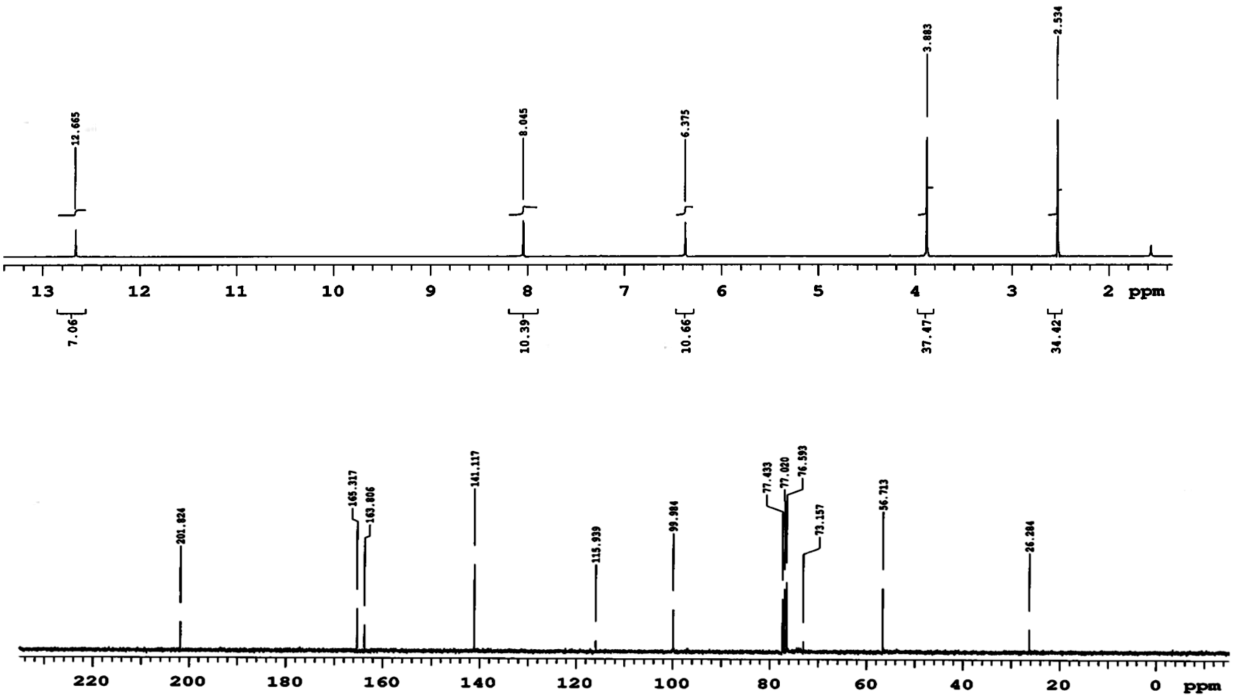

Figure S1.4. ^{1}H- and ^{13}C-NMR spectra of **1d** in $CDCl_3$ at 300 and 75 MHz, respectively.

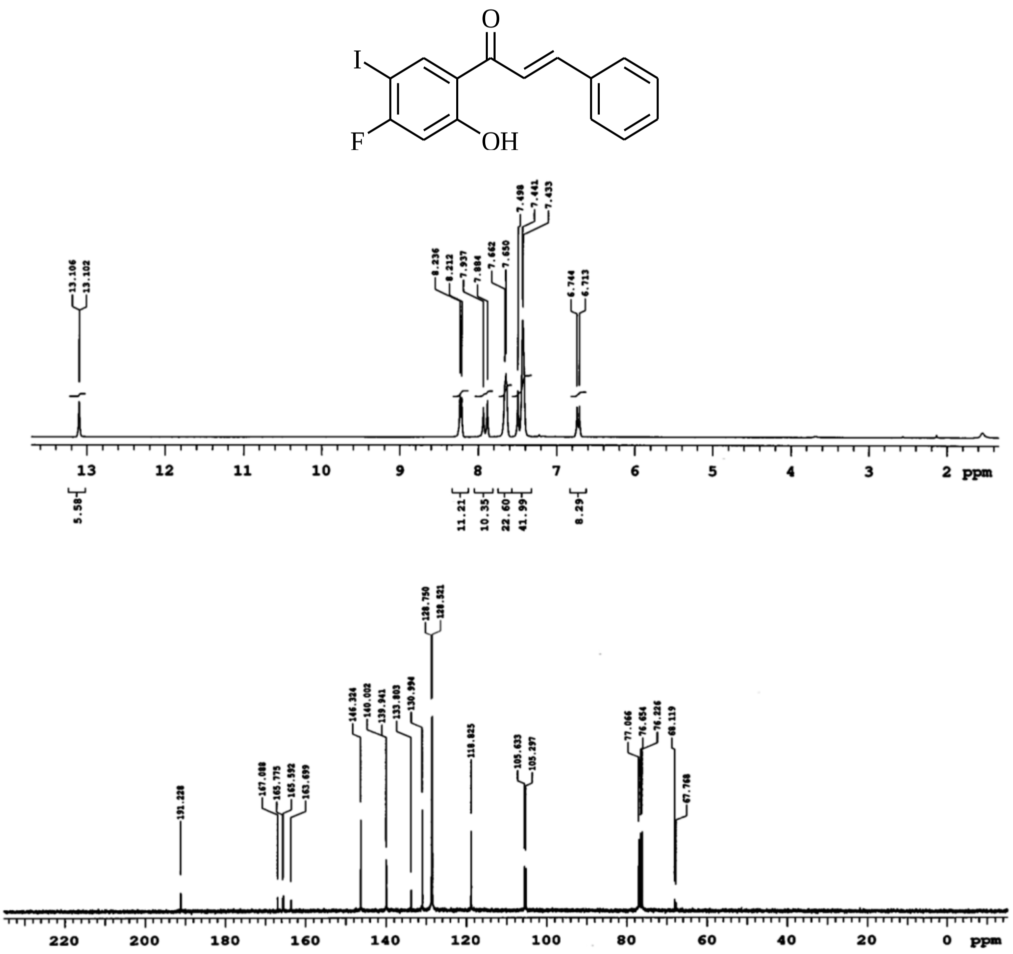

Figure S1.5. ^{1}H- and ^{13}C-NMR spectra of **2a** in DMSO-d_6 at 500 and 125 MHz, respectively.

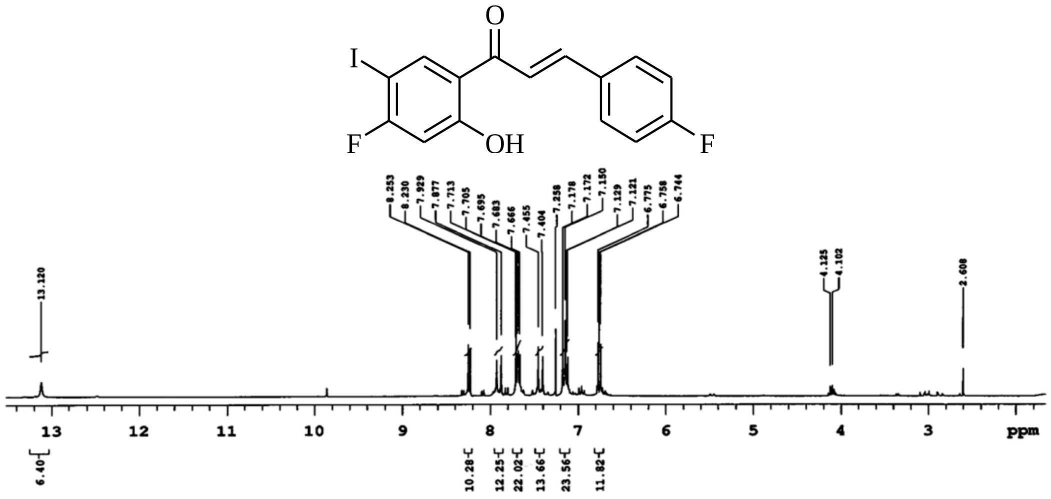

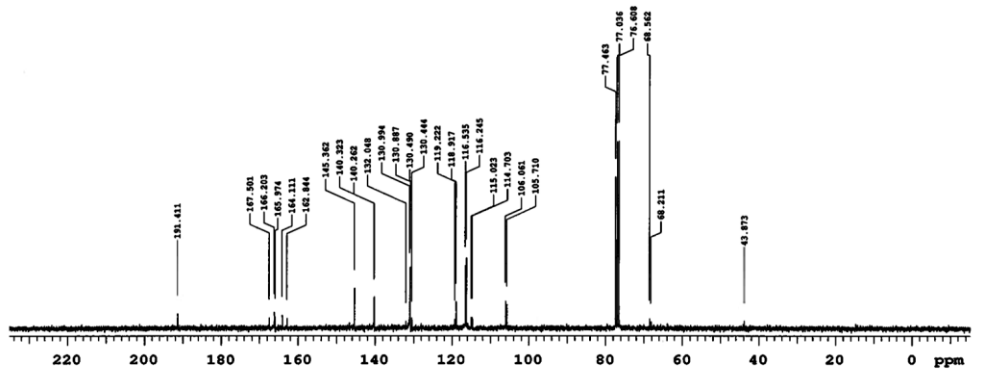

Figure S1.6. ^{1}H- and ^{13}C-NMR spectra of **2b** in DMSO-d_6 at 500 and 125 MHz, respectively.

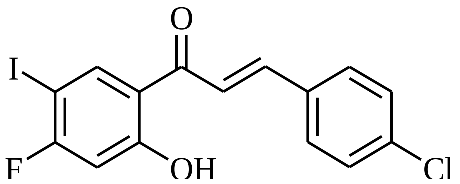

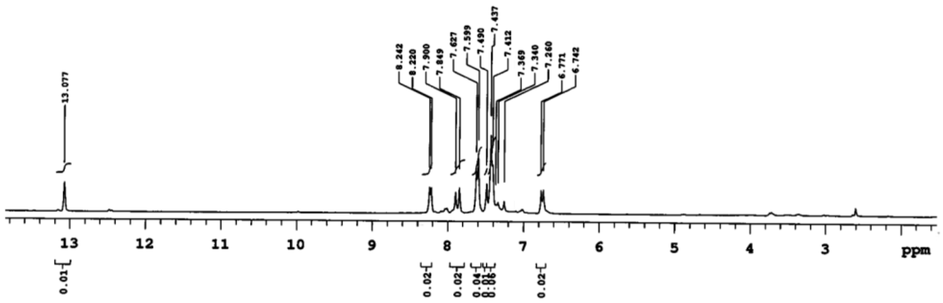

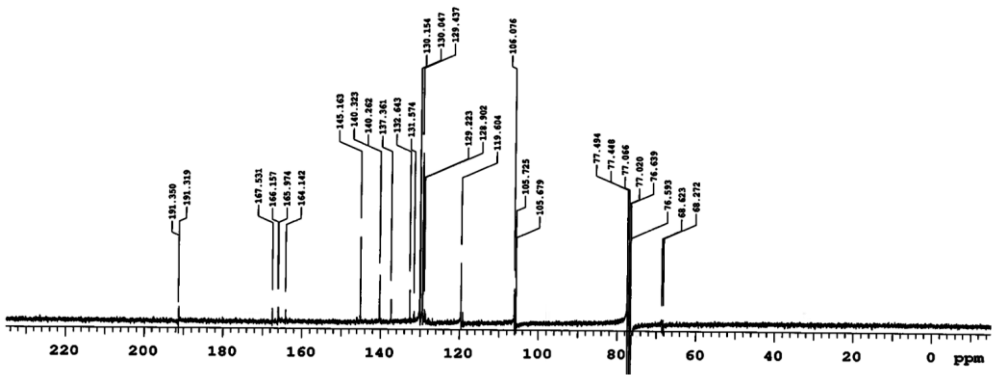

Figure S1.7. ^{1}H- and ^{13}C-NMR spectra of **2c** in DMSO-d_6 at 500 and 125 MHz, respectively.

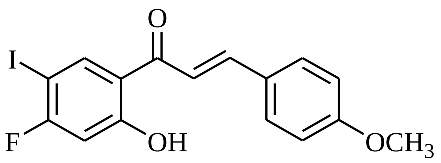

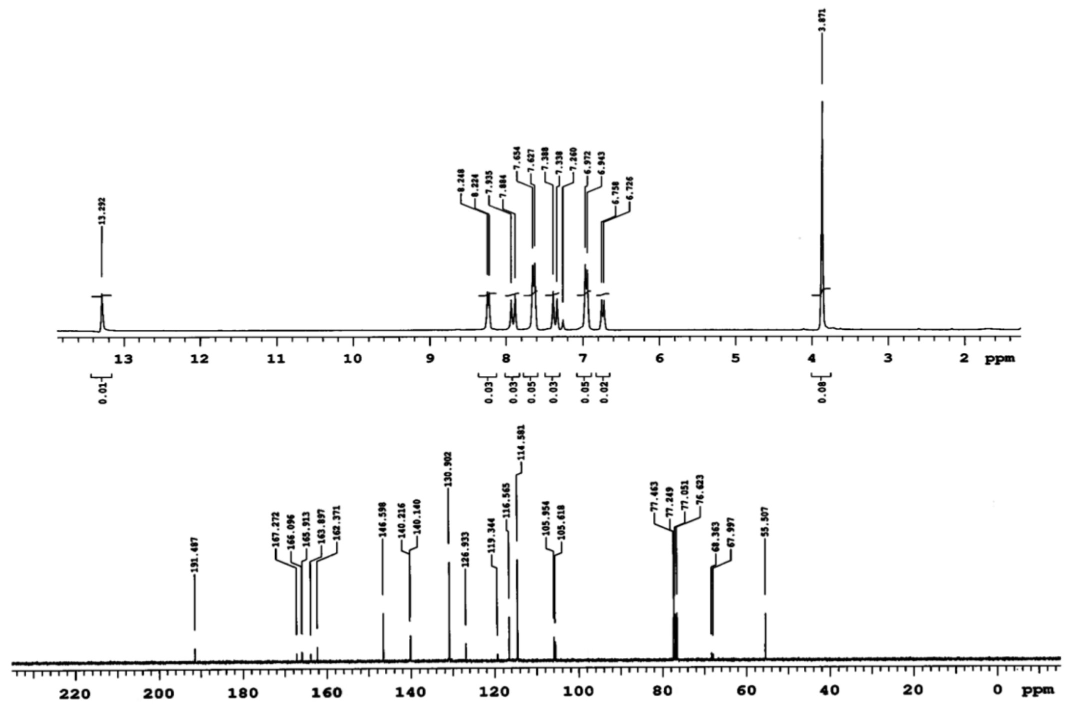

Figure S1.8. ^{1}H- and ^{13}C-NMR spectra of **2d** in DMSO-d_6 at 500 and 125 MHz, respectively.

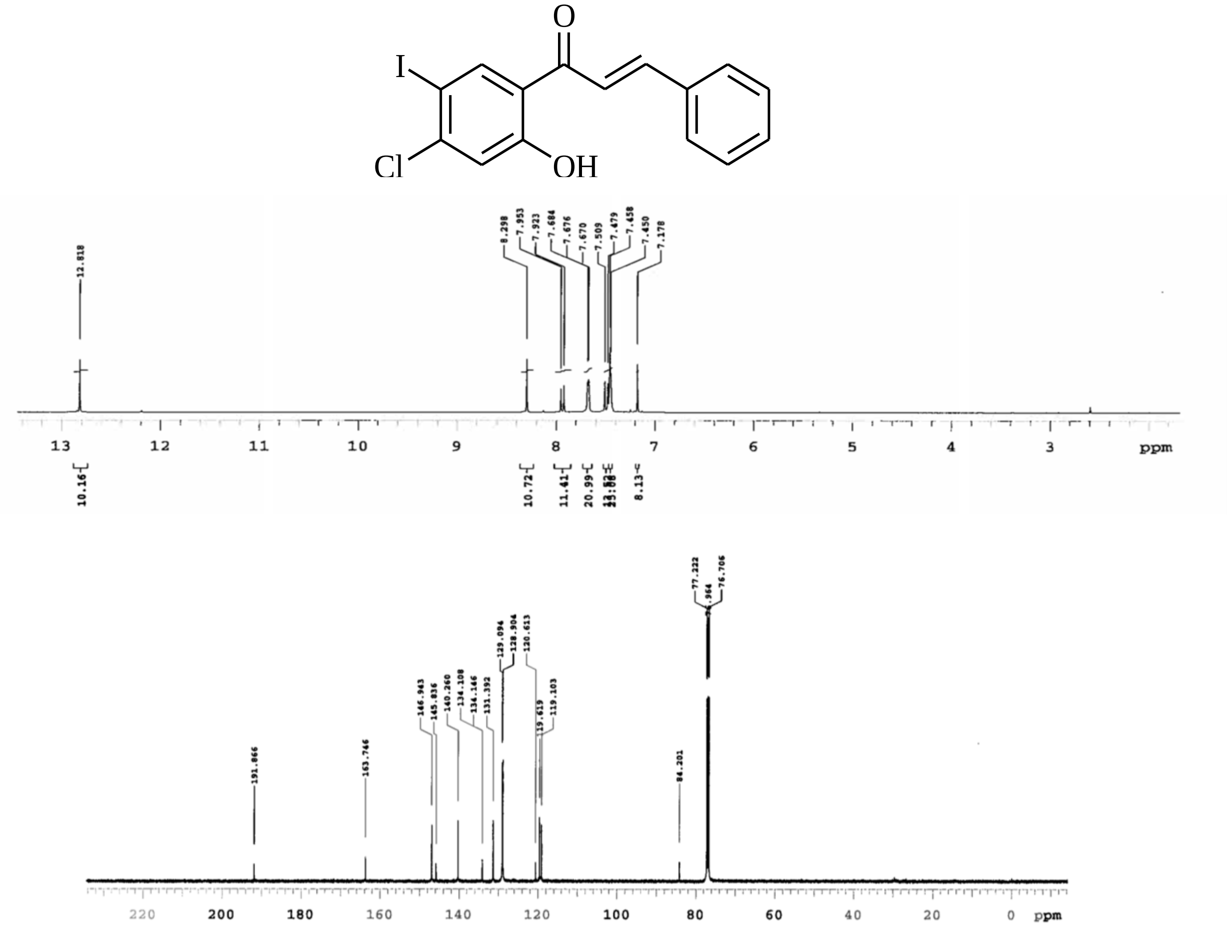

Figure S1.9. ^{1}H- and ^{13}C-NMR spectra of **2e** in DMSO-d_6 at 500 and 125 MHz, respectively.

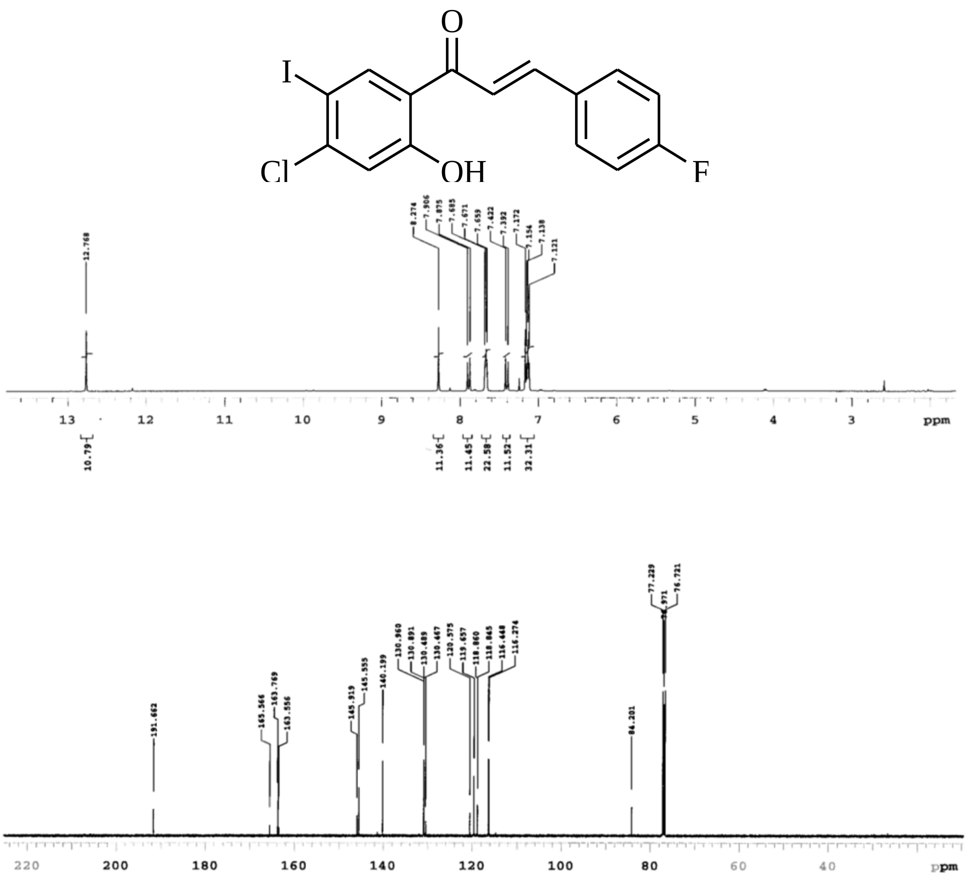

Figure S1.10: ^{1}H- and ^{13}C-NMR spectra of **2f** in DMSO-d_6 at 500 and 125 MHz, respectively.

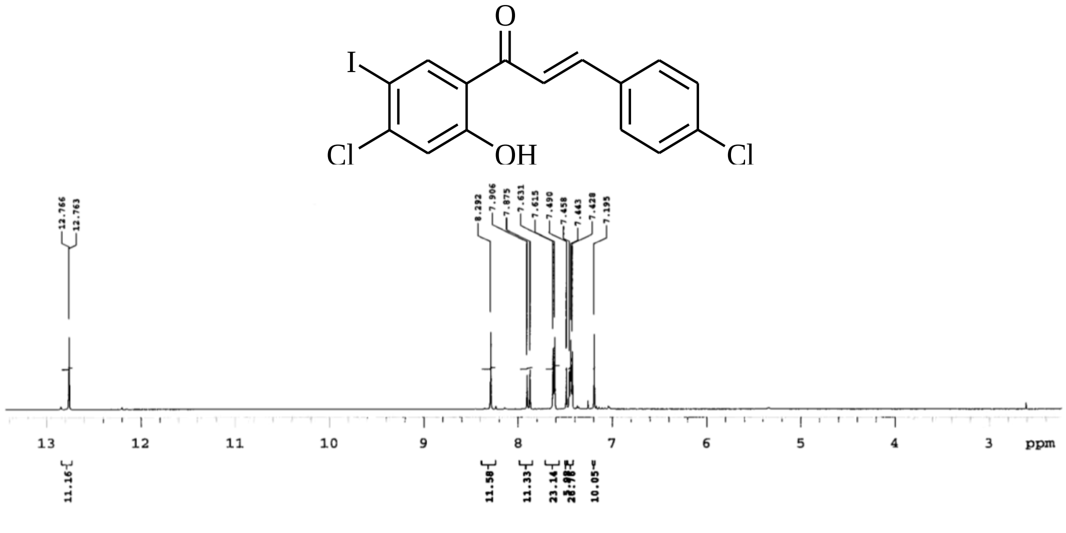

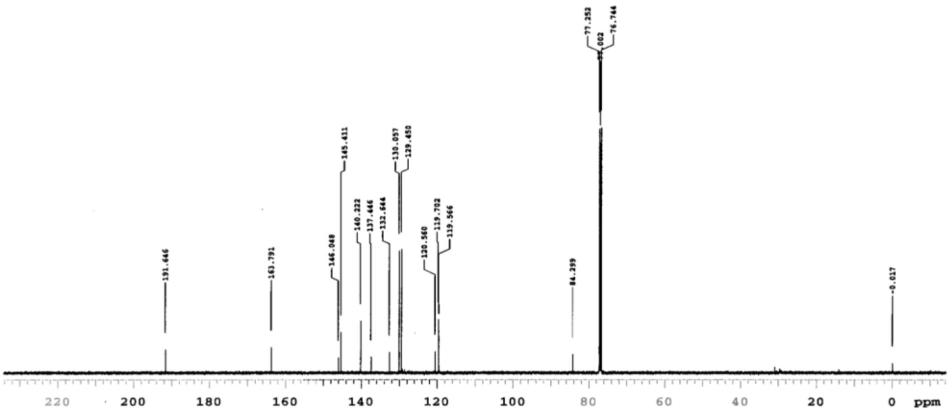

Figure S1.11: ^{1}H- and ^{13}C-NMR spectra of **2g** in DMSO-*d*6 at 500 and 125 MHz, respectively.

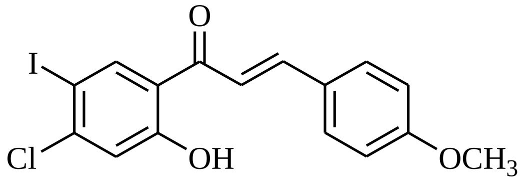

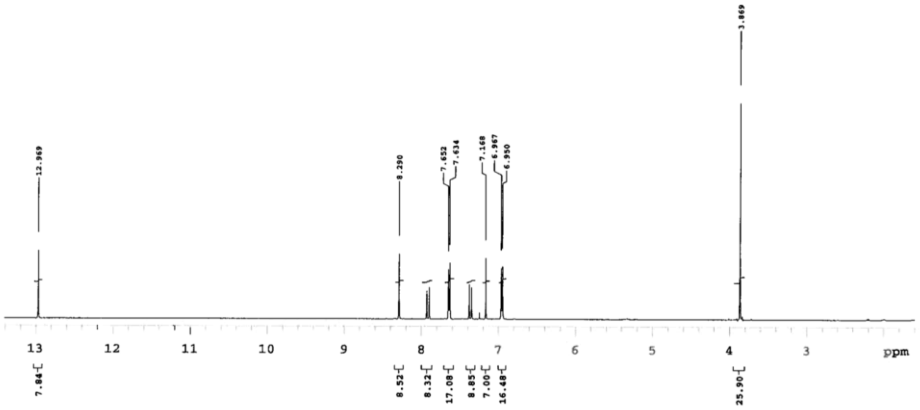

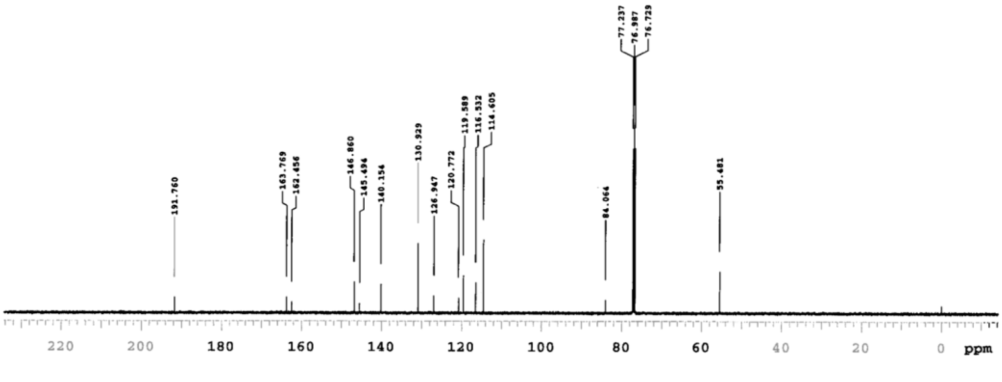

Figure S1.12: ^{1}H- and ^{13}C-NMR spectra of **2h** in DMSO-*d*6 at 500 and 125 MHz, respectively.

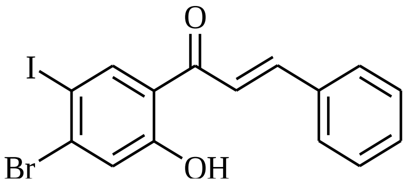

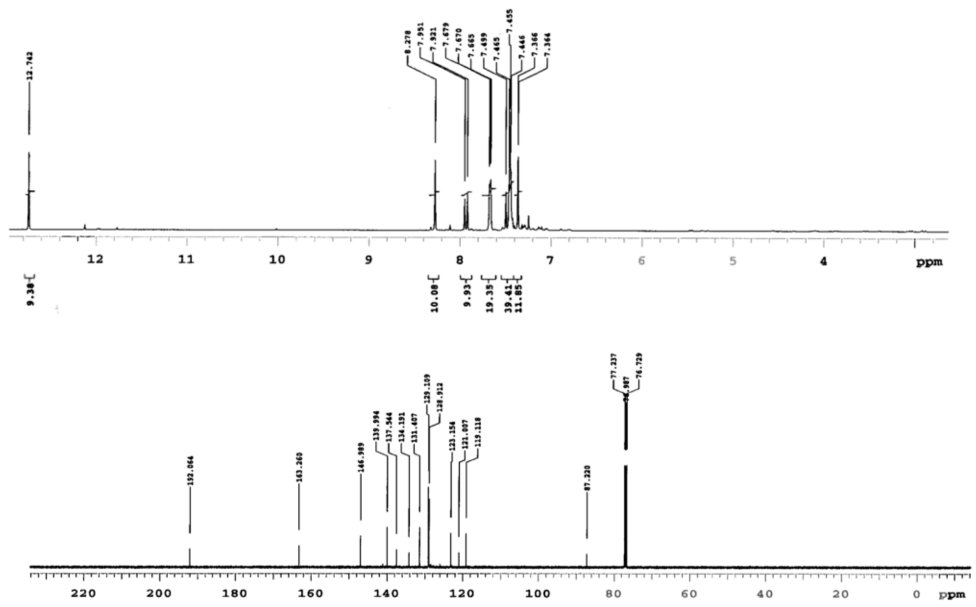

Figure S1.13: ^{1}H- and ^{13}C-NMR spectra of **2i** in DMSO-d_6 at 500 and 125 MHz, respectively.

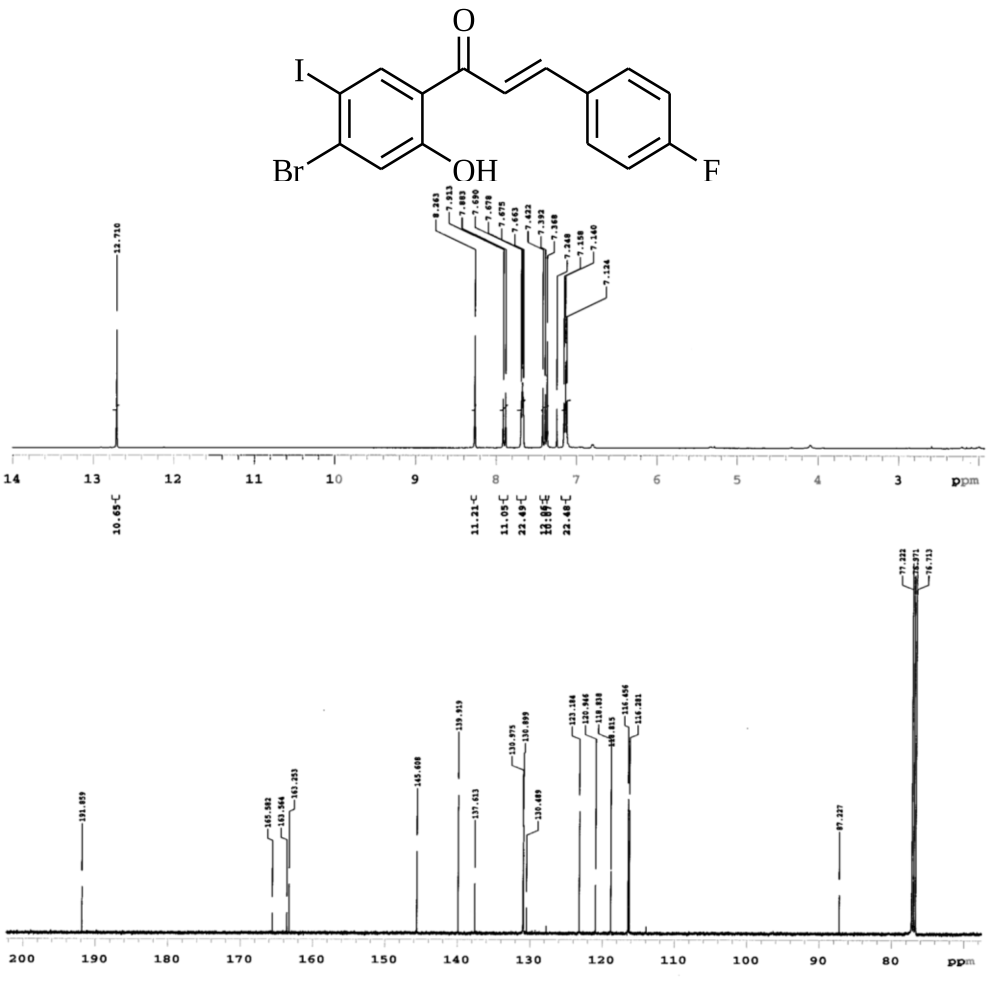

Figure S1.14: ^{1}H- and ^{13}C-NMR spectra of **2j** in DMSO-d_6 at 500 and 125 MHz, respectively.

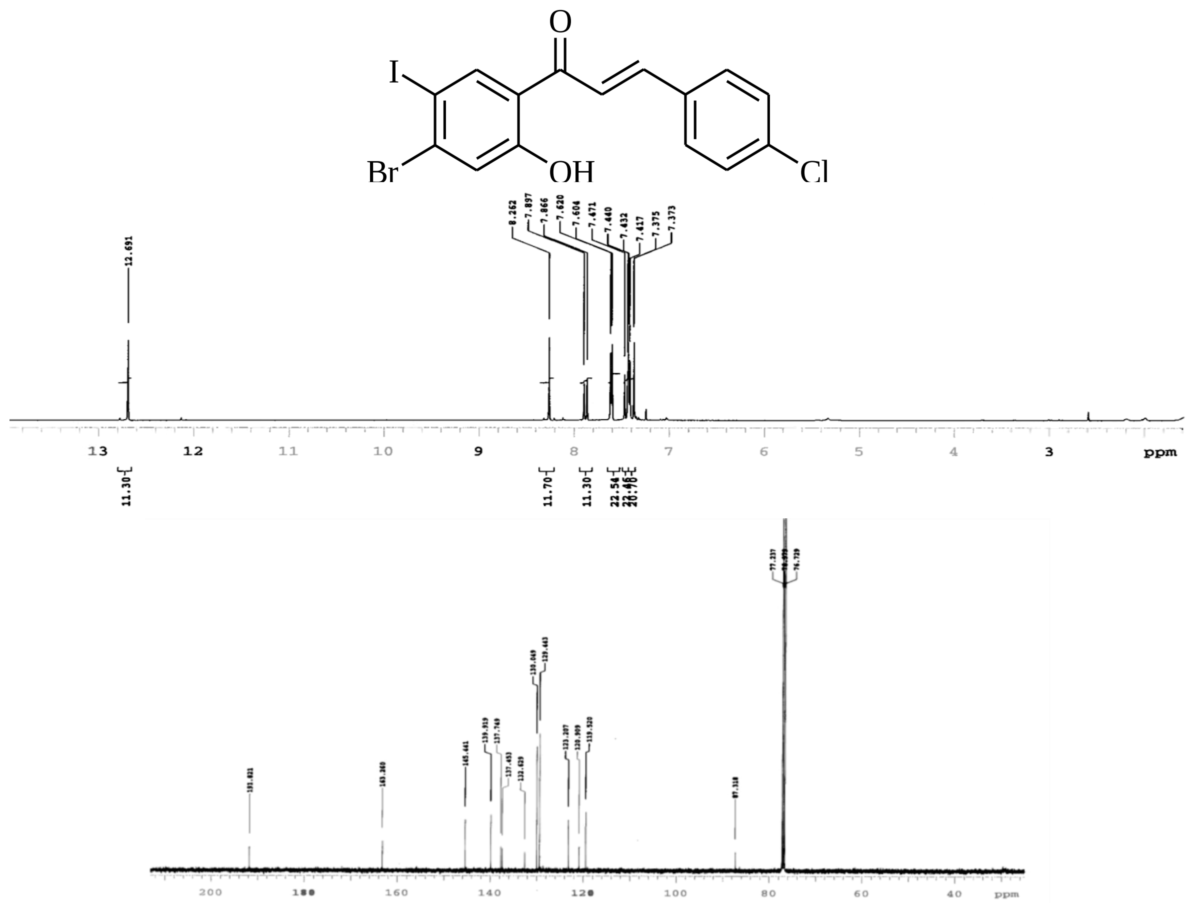

Figure S1.15: ^{1}H- and ^{13}C-NMR spectra of **2k** in DMSO-d_6 at 500 and 125 MHz, respectively.

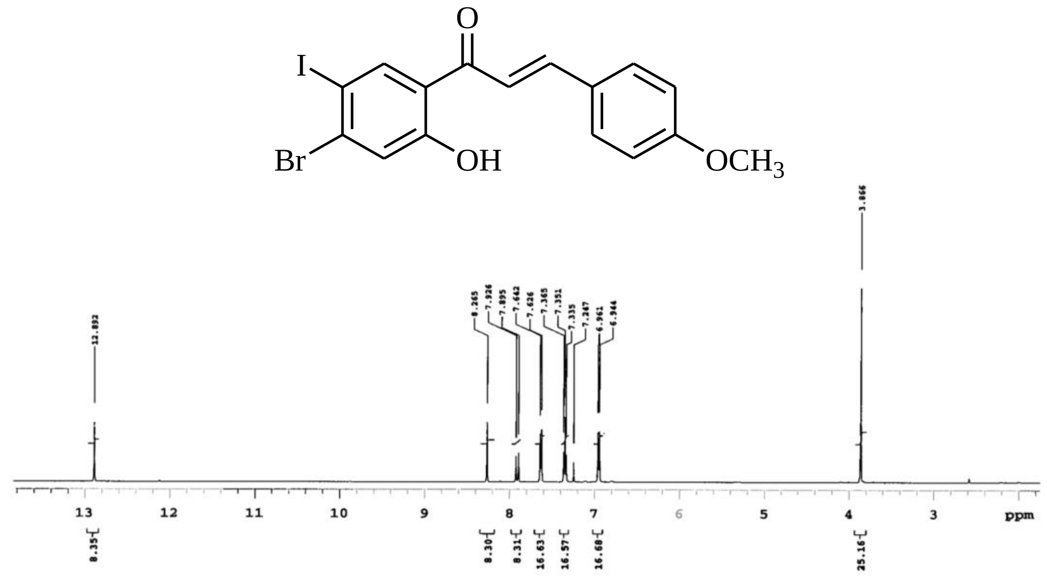

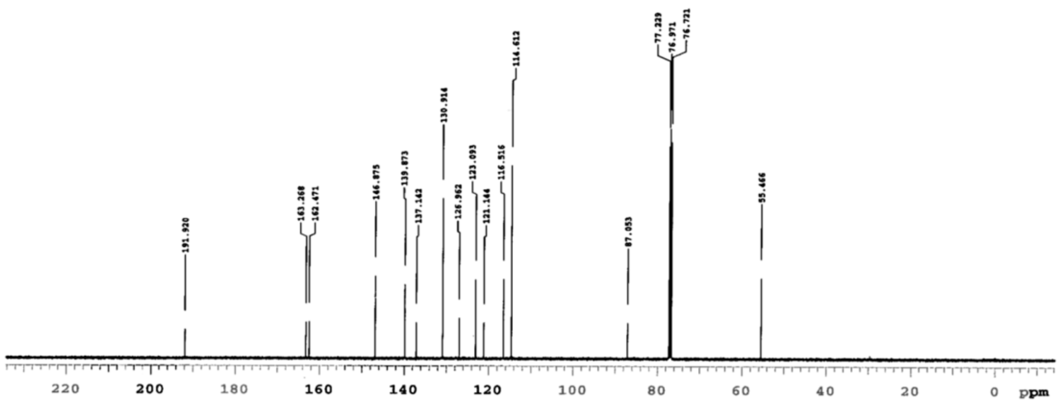

Figure S1.16: ^{1}H- and ^{13}C-NMR spectra of **2l** in DMSO-*d*6 at 500 and 125 MHz, respectively.

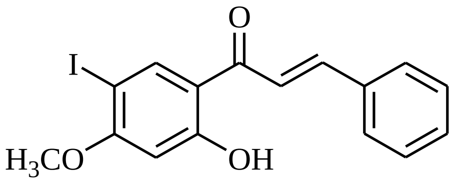

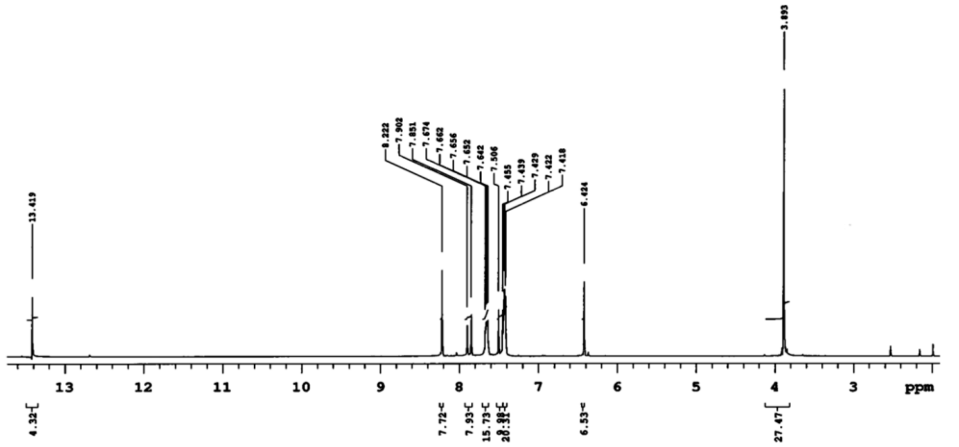

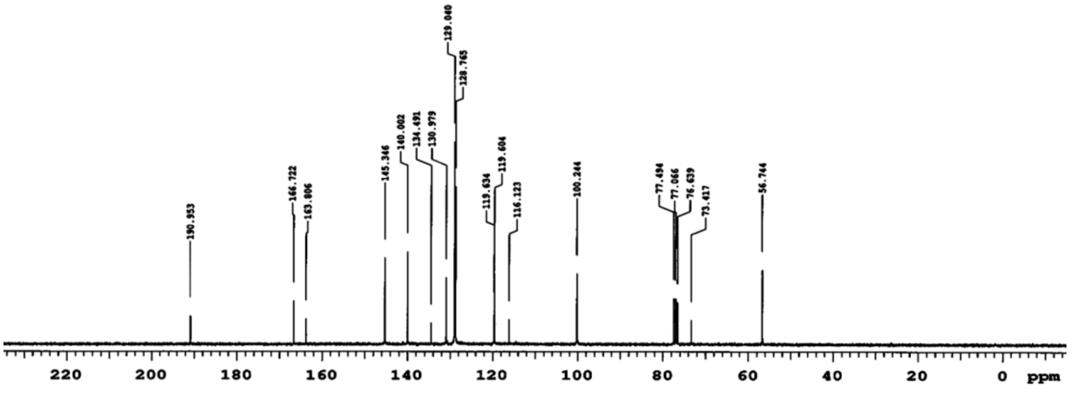

Figure S1.17: ^{1}H- and ^{13}C-NMR spectra of **2m** in DMSO-*d*6 at 500 and 125 MHz, respectively.

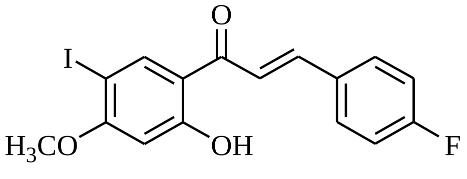

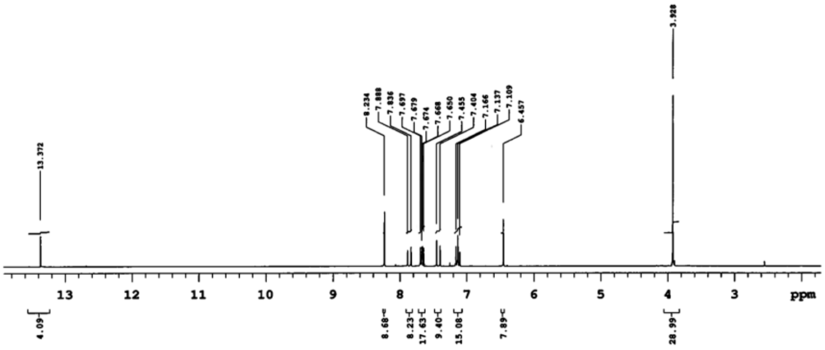

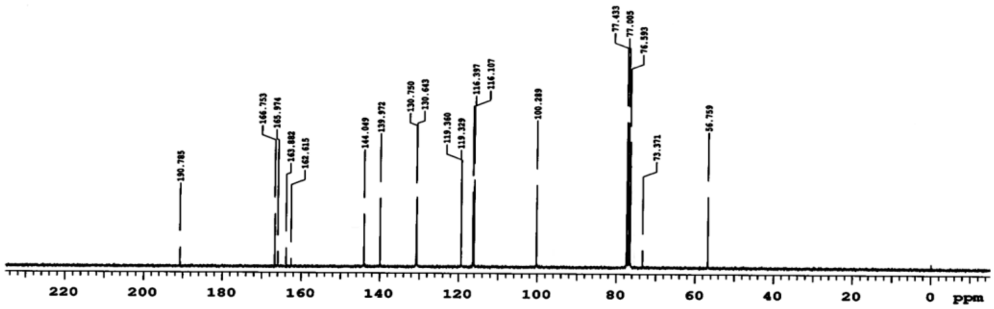

Figure S1.18: ^{1}H- and ^{13}C-NMR spectra of **2n** in DMSO-d_6 at 500 and 125 MHz, respectively.

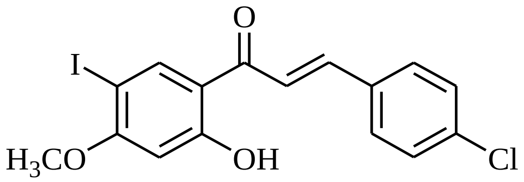

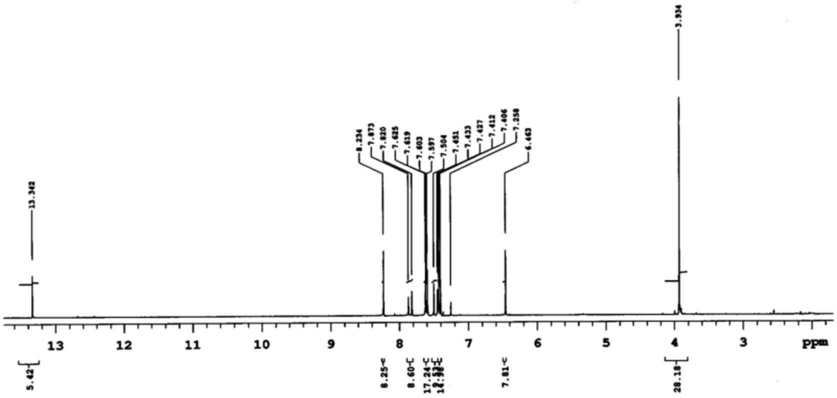

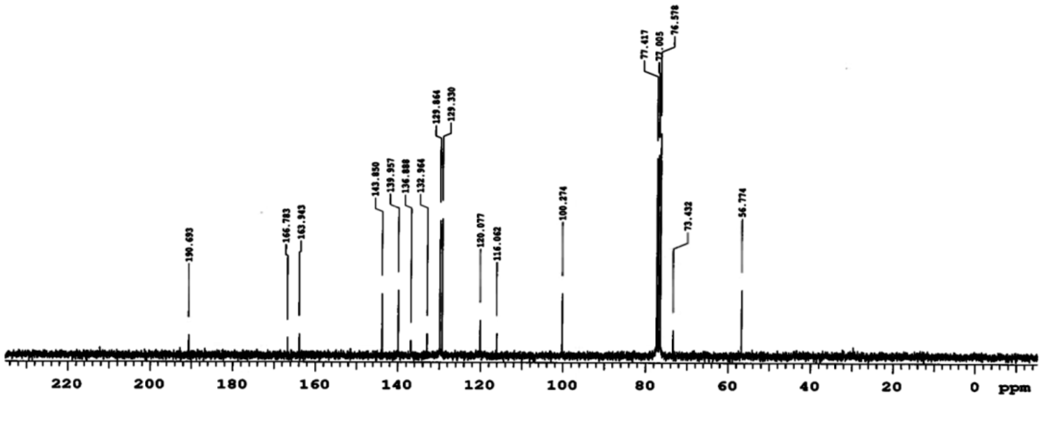

Figure S1.19: ^{1}H- and ^{13}C-NMR spectra of **2o** in DMSO-*d*6 at 500 and 125 MHz, respectively.

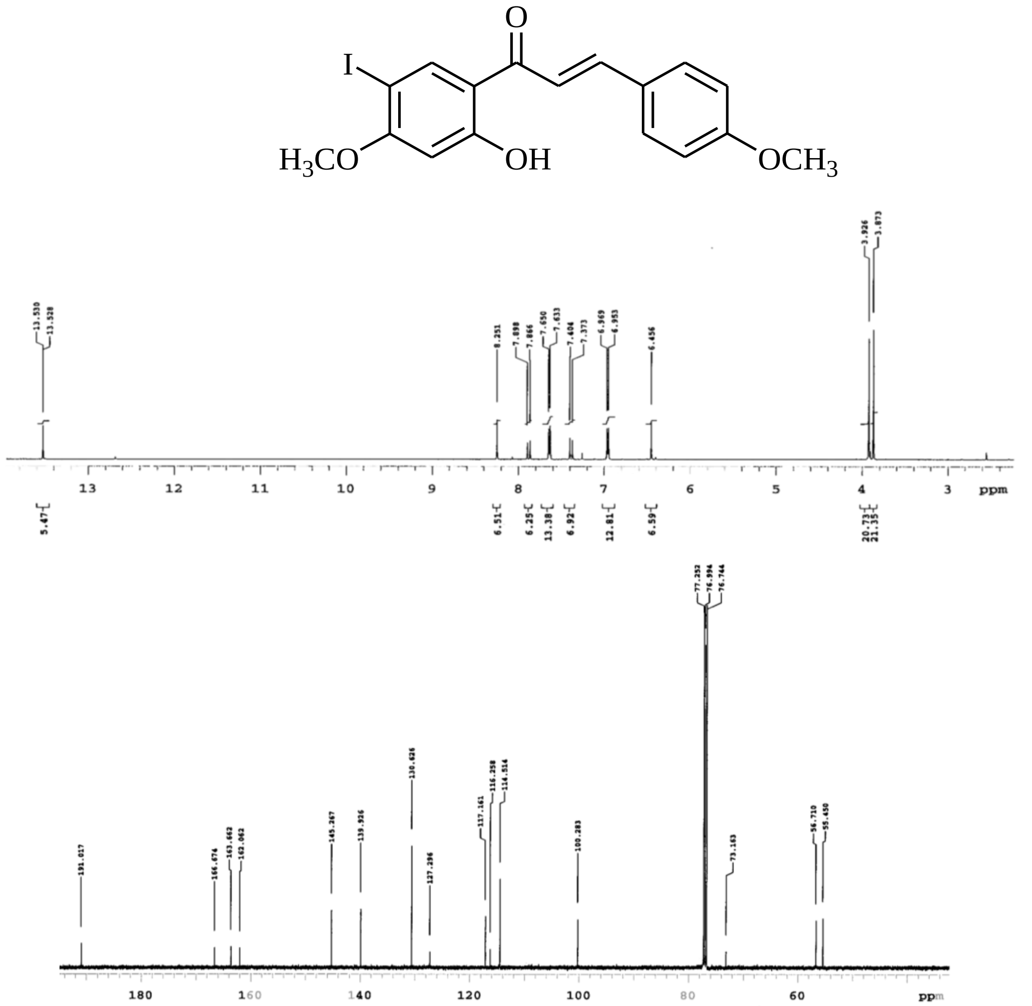

Figure S1.20: ^{1}H- and ^{13}C-NMR spectra of **2p** in DMSO-*d*6 at 500 and 125 MHz, respectively.

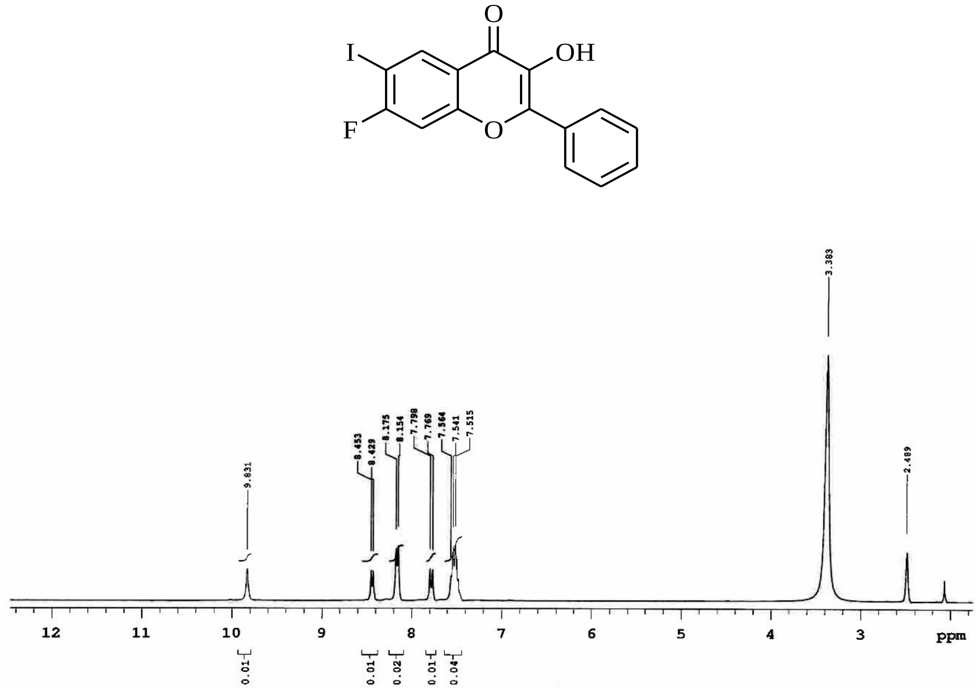

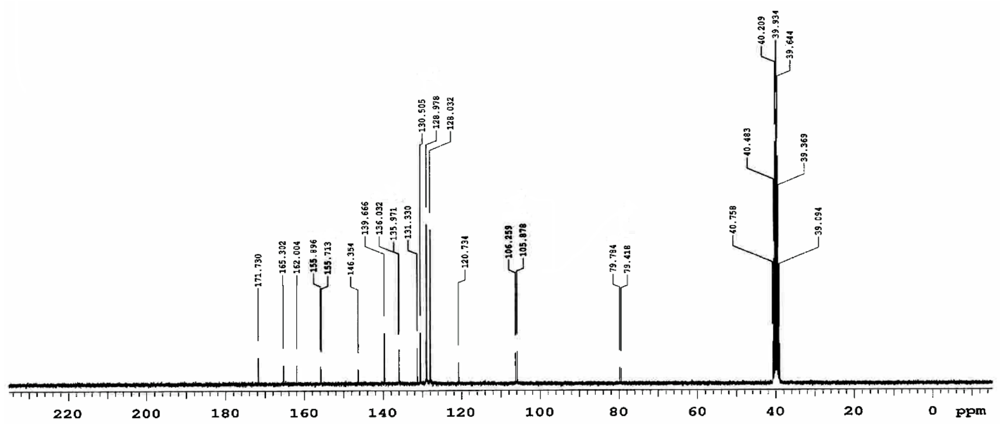

Figure S1.21: ^{1}H- and ^{13}C-NMR spectra of **3a** in DMSO-*d*6 at 500 and 125 MHz, respectively.

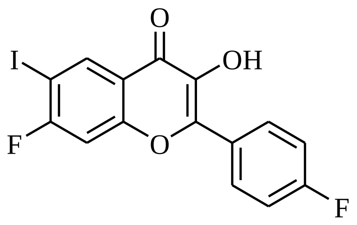

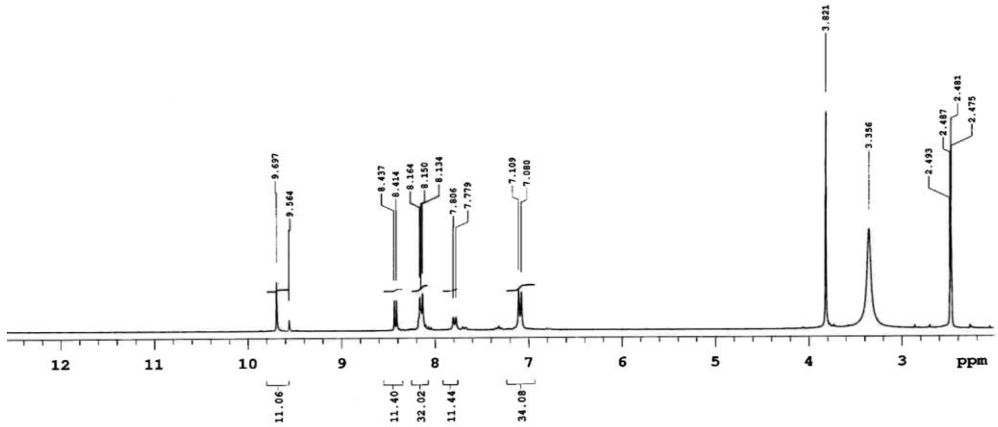

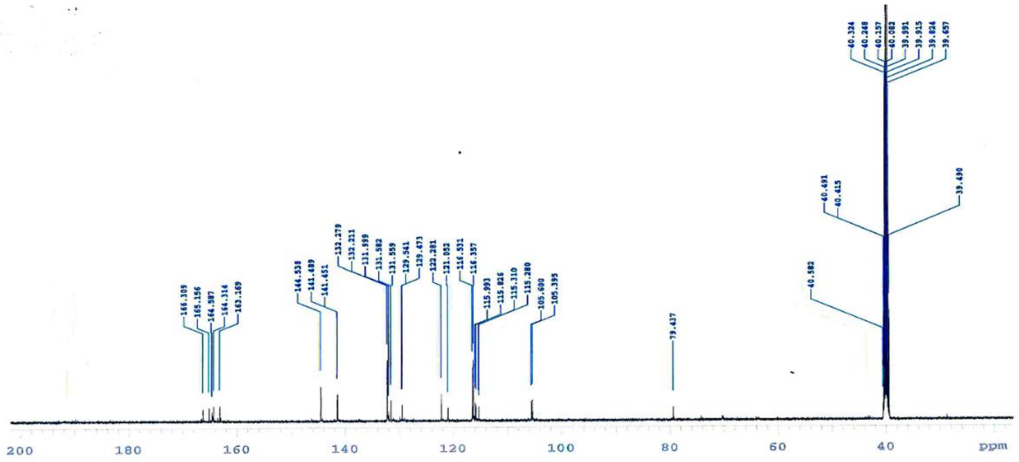

Figure S1.22: ^{1}H- and ^{13}C-NMR spectra of **3b** in DMSO-*d*6 at 500 and 125 MHz, respectively.

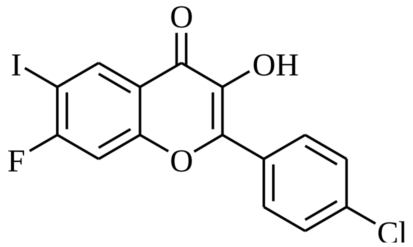

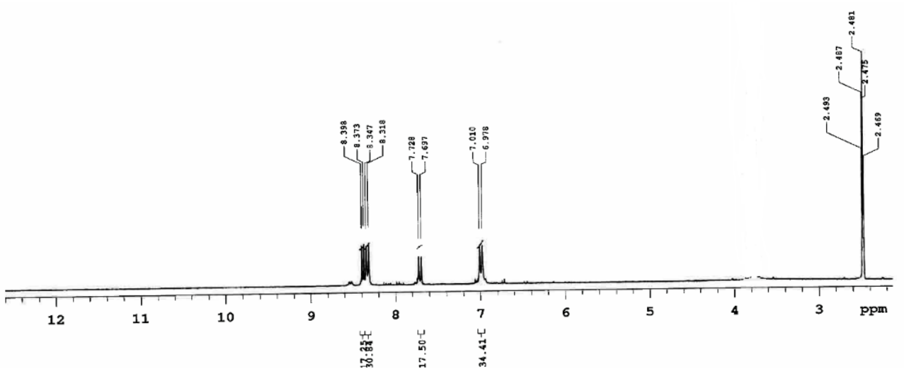

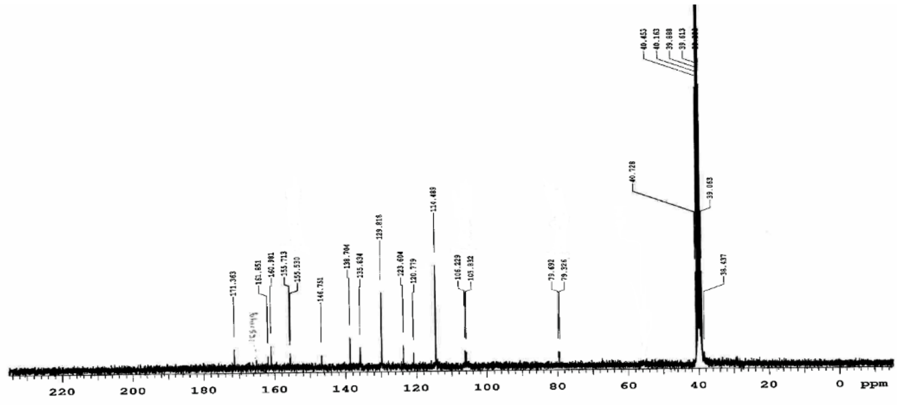

Figure S1.23: ^{1}H- and ^{13}C-NMR spectra of **3c** in DMSO-d_6 at 500 and 125 MHz, respectively.

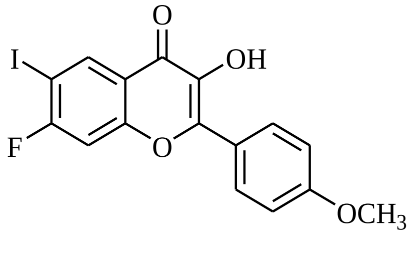

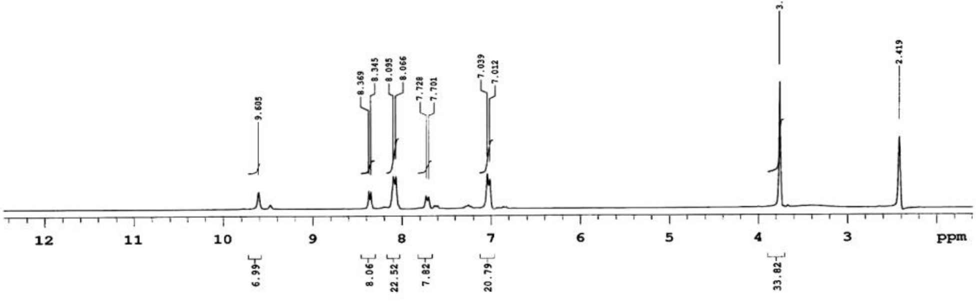

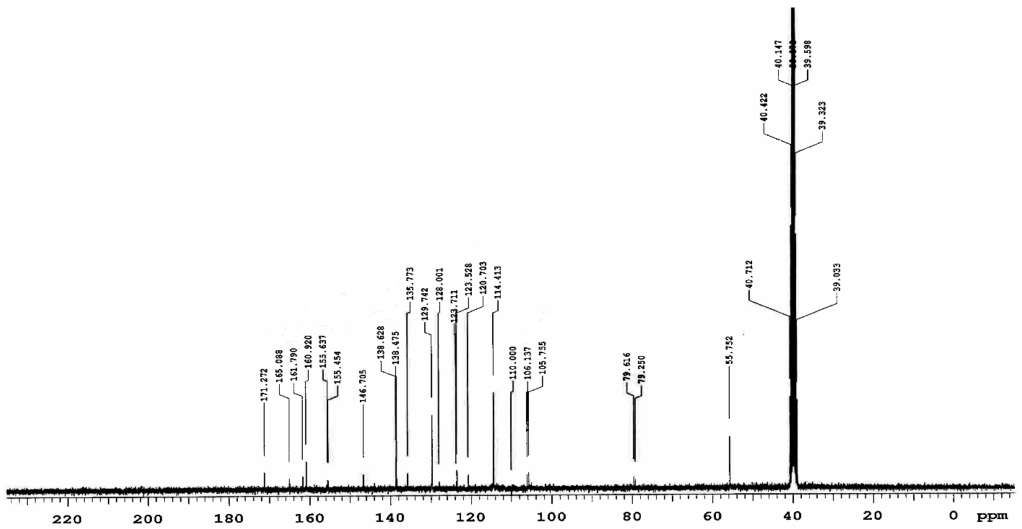

Figure S1.24: ^{1}H- and ^{13}C-NMR spectra of **3d** in DMSO-*d*6 at 500 and 125 MHz, respectively.

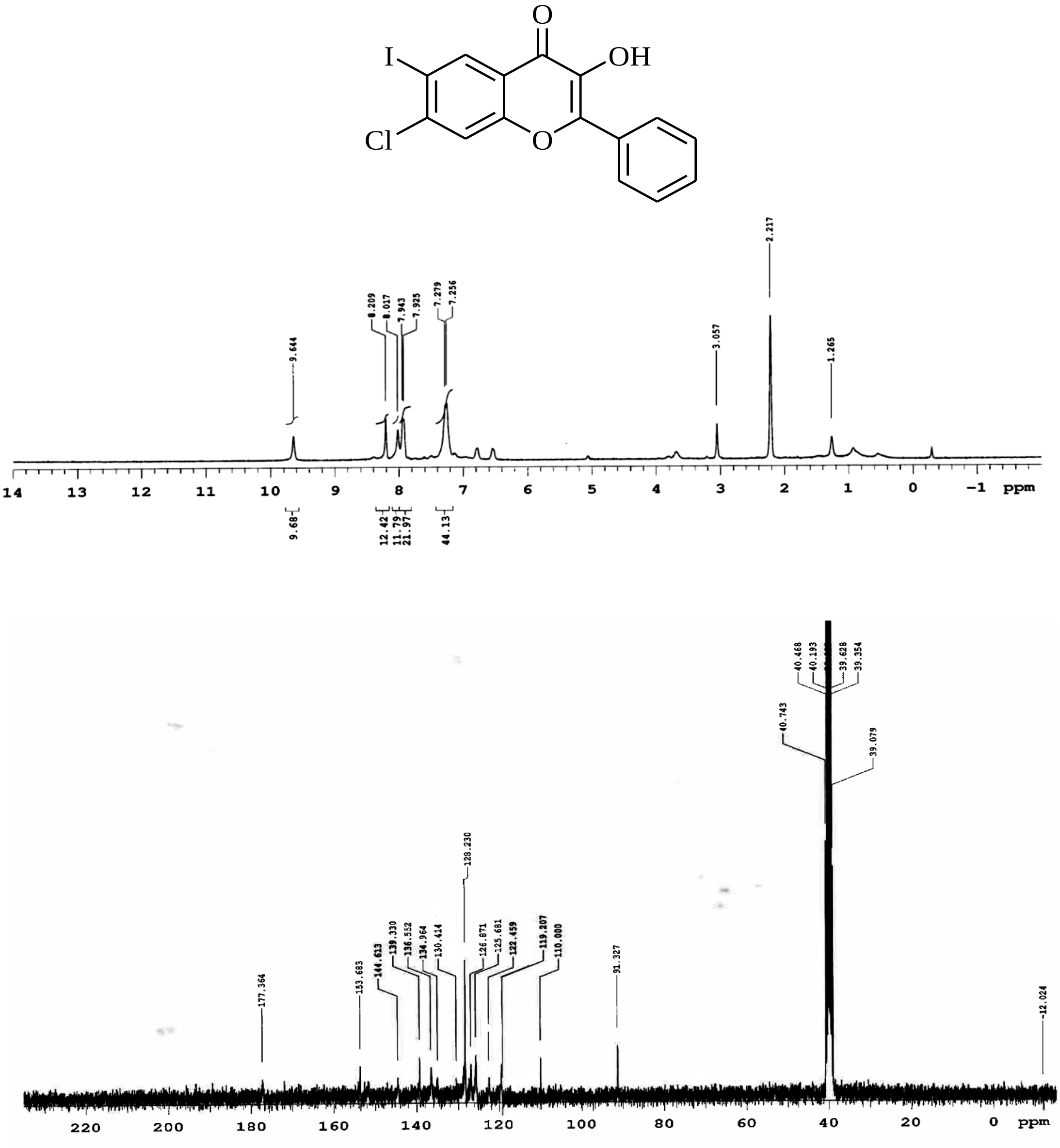

Figure S1.25: ^{1}H- and ^{13}C-NMR spectra of **3e** in DMSO-*d*6 at 500 and 125 MHz, respectively.

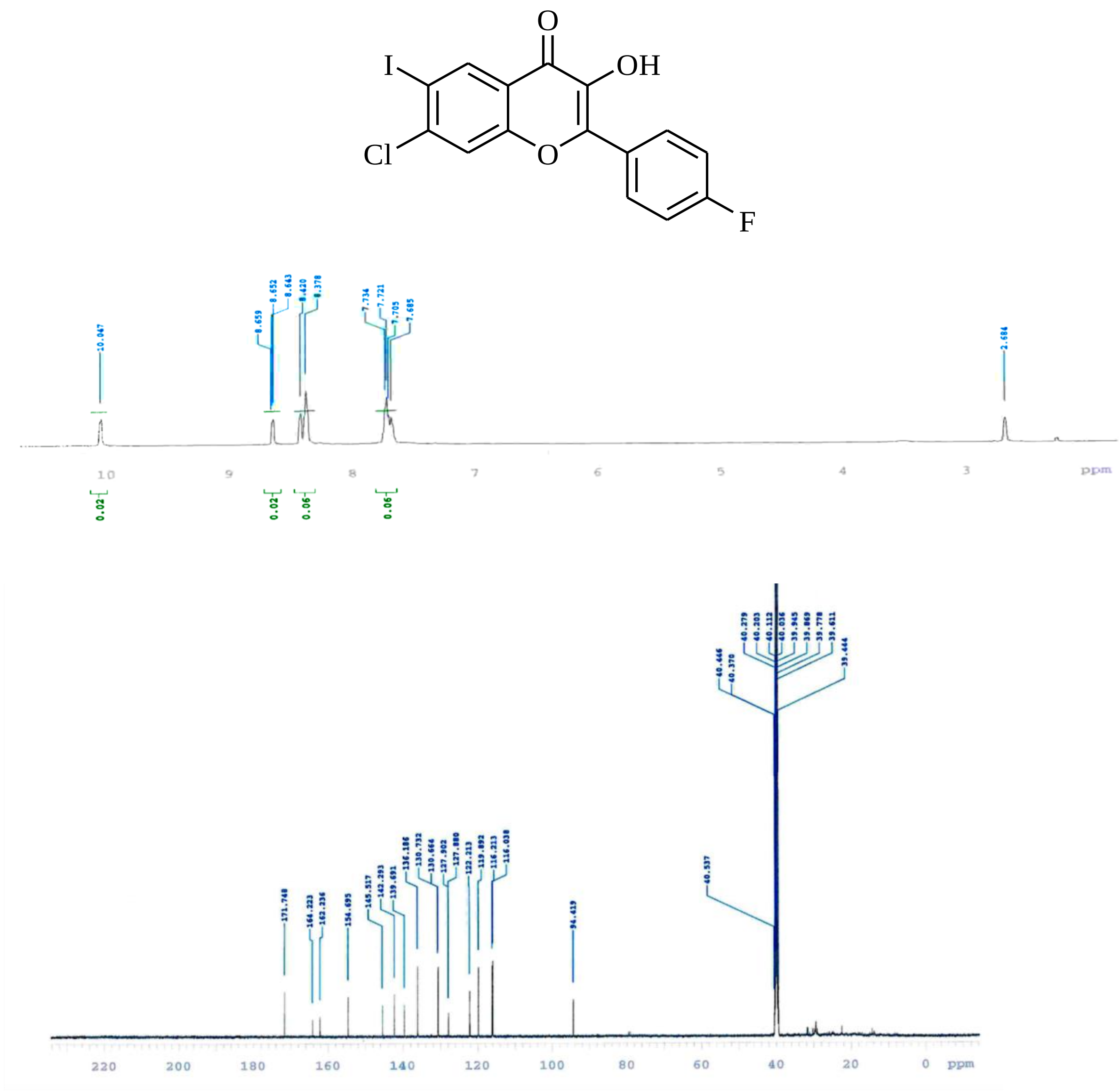

Figure S1.26: ^{1}H- and ^{13}C-NMR spectra of **3f** in DMSO-d_6 at 500 and 125 MHz, respectively.

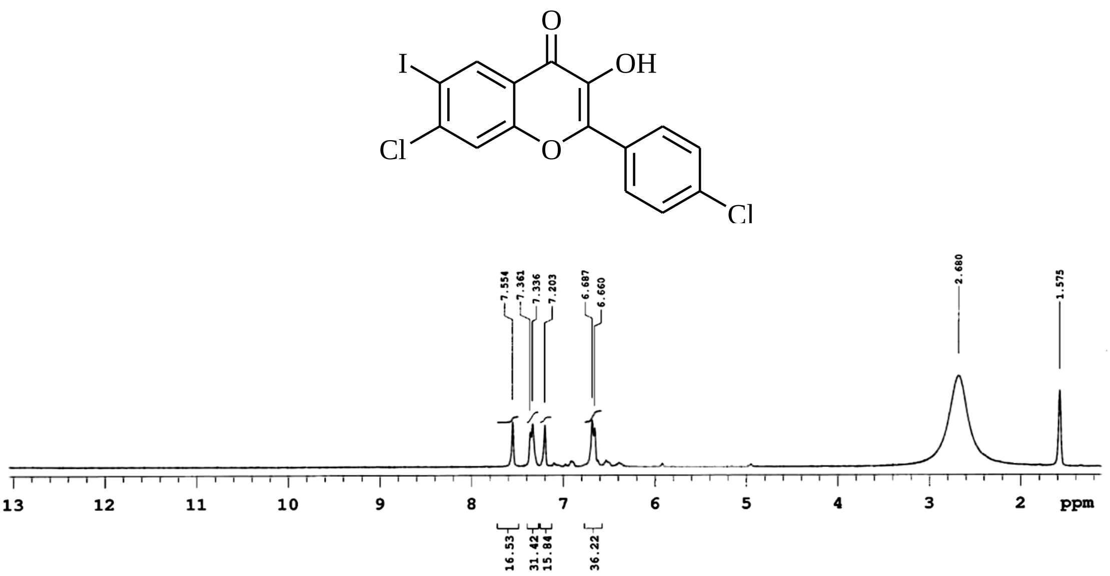

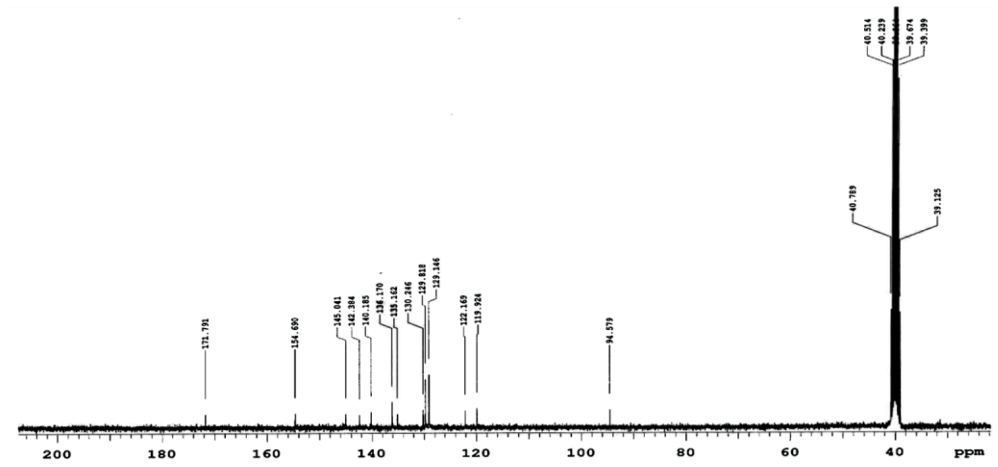

Figure S1.27: ^{1}H- and ^{13}C-NMR spectra of **3g** in DMSO-*d*6 at 500 and 125 MHz, respectively.

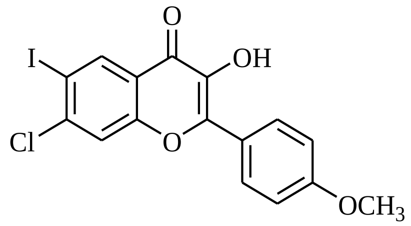

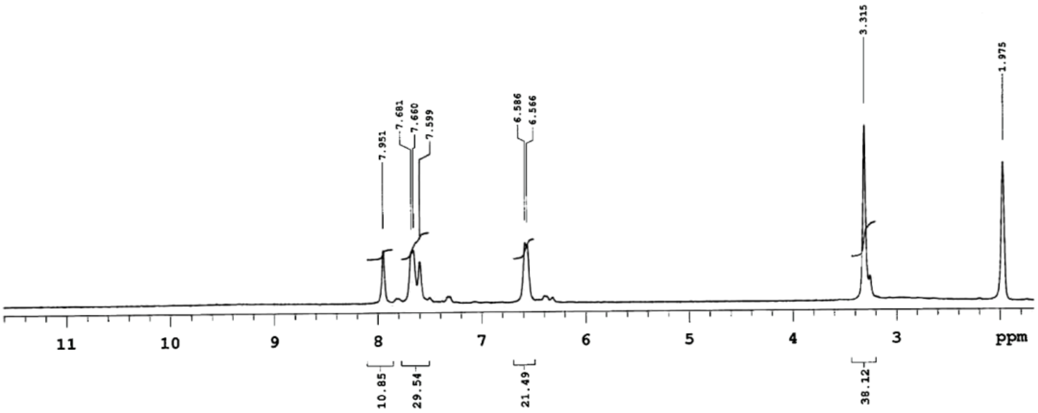

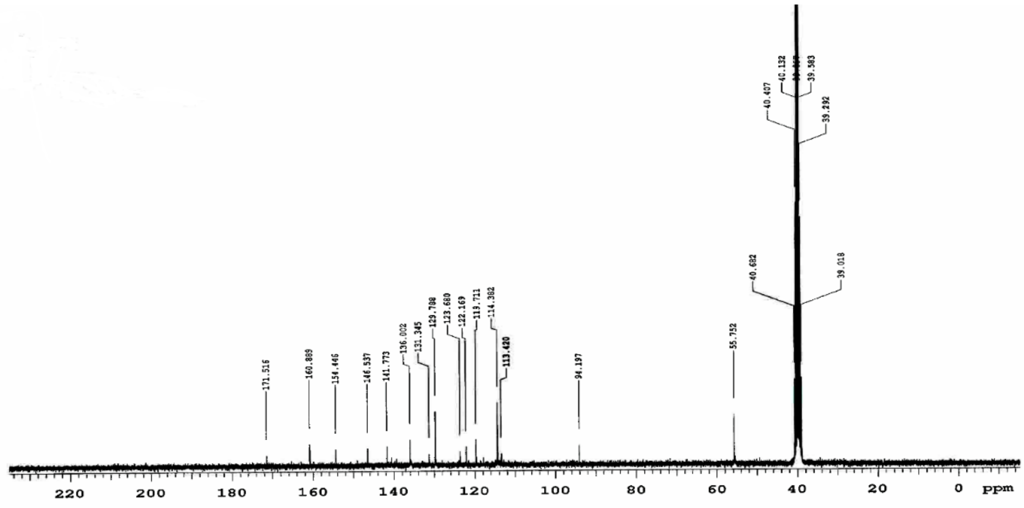

Figure S1.28: ^{1}H- and ^{13}C-NMR spectra of **3h** in DMSO-*d*6 at 500 and 125 MHz, respectively.

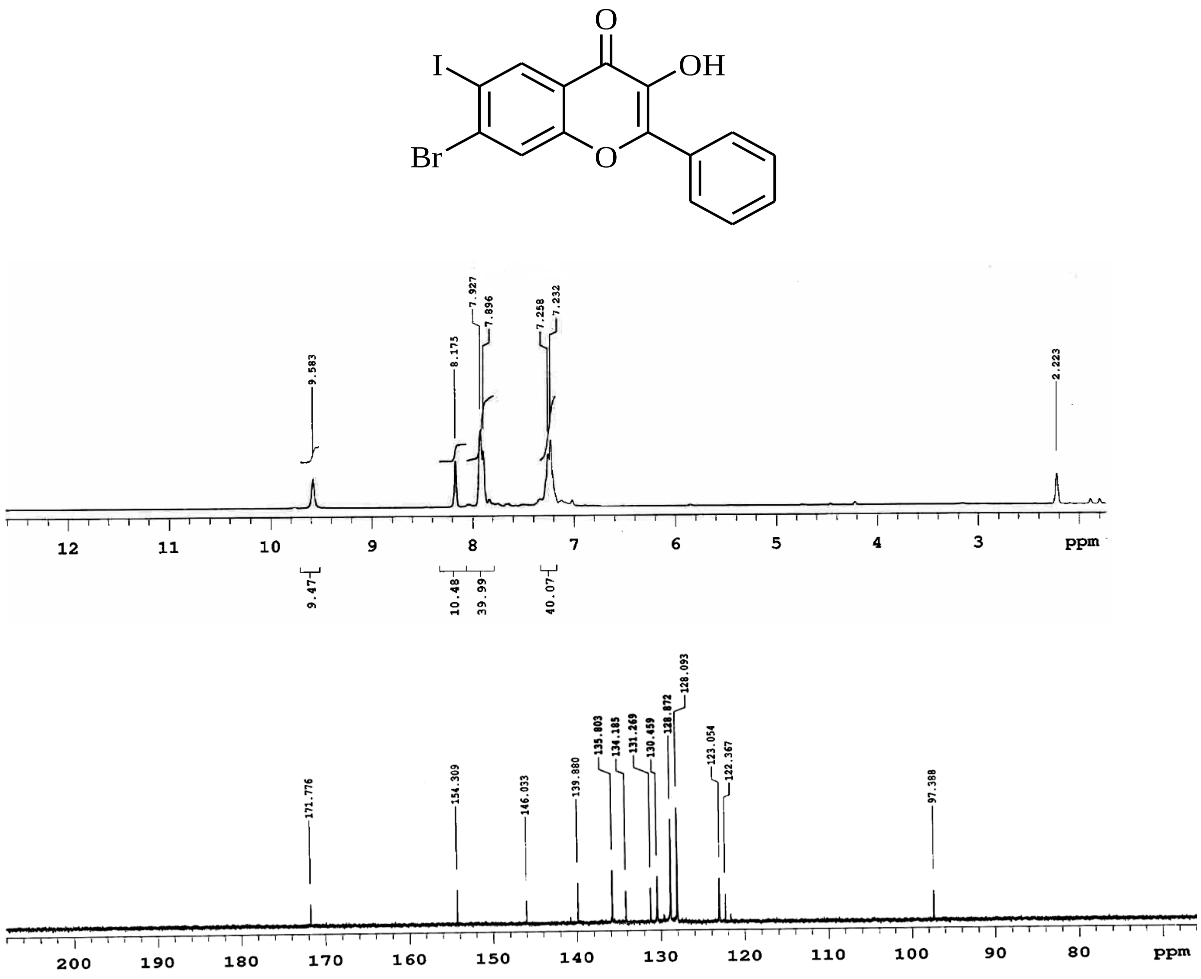

Figure S1.29: ^{1}H- and ^{13}C-NMR spectra of **3i** in DMSO-d_6 at 500 and 125 MHz, respectively.

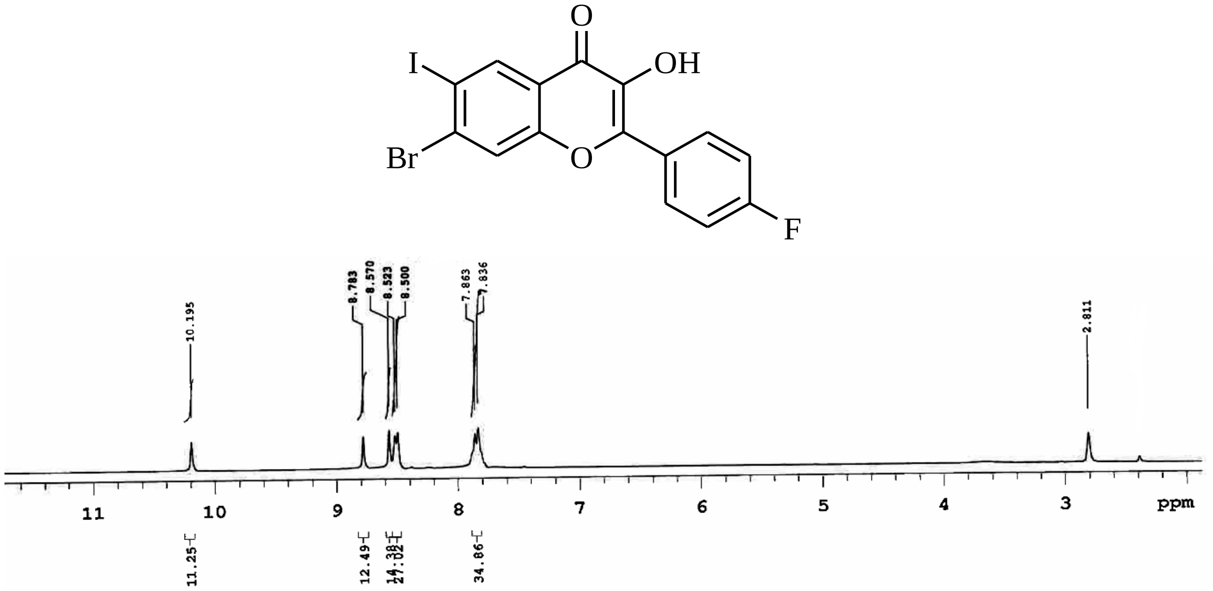

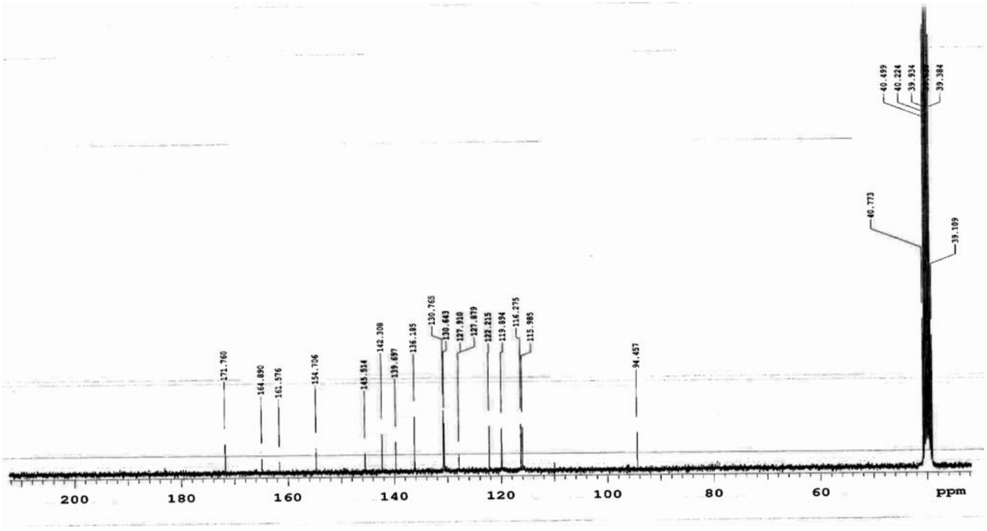

Figure S1.30: ^{1}H- and ^{13}C-NMR spectra of **3j** in DMSO-*d*6 at 500 and 125 MHz, respectively.

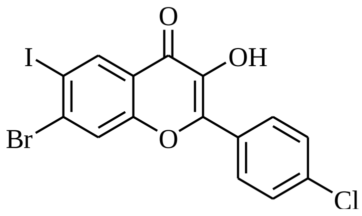

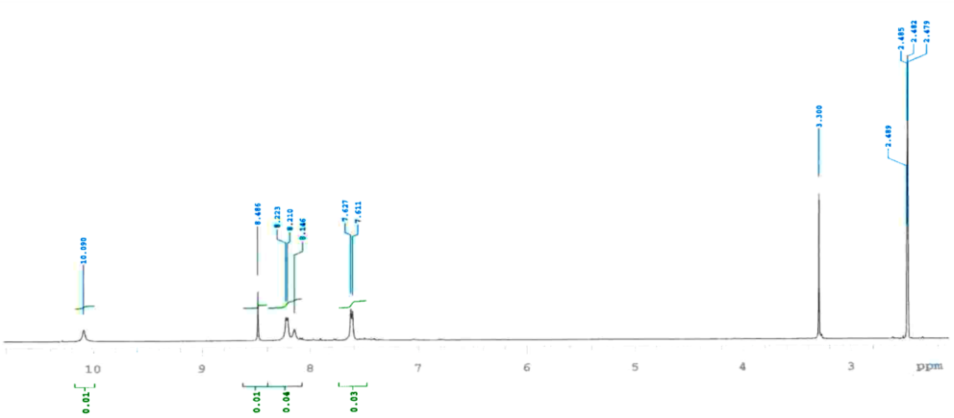

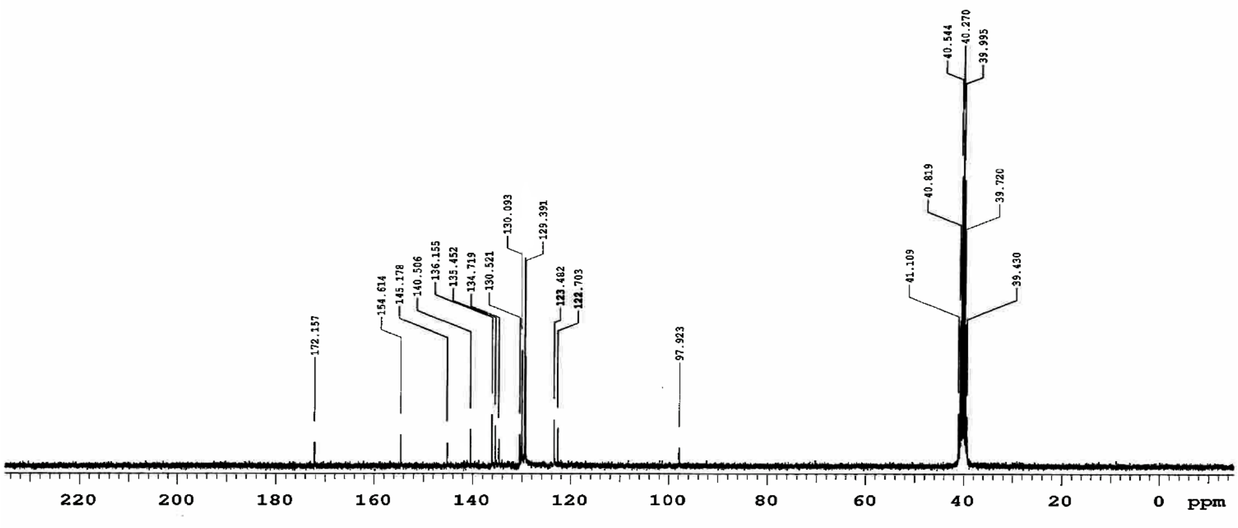

Figure S1.31: ^{1}H- and ^{13}C-NMR spectra of **3k** in DMSO-*d*6 at 500 and 125 MHz, respectively.

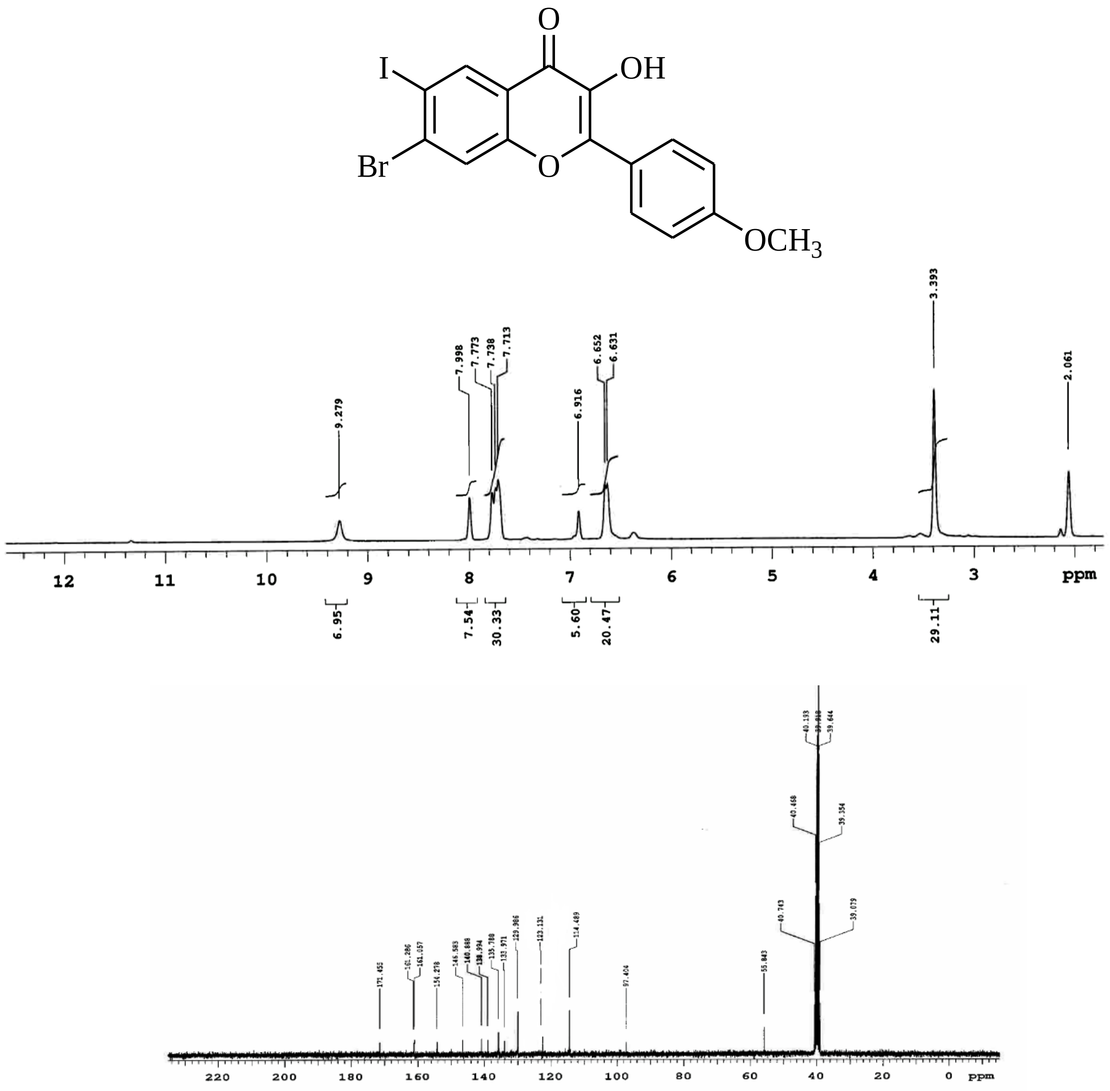

Figure S1.32: ^{1}H- and ^{13}C-NMR spectra of **3l** in DMSO-*d*6 at 500 and 125 MHz, respectively.

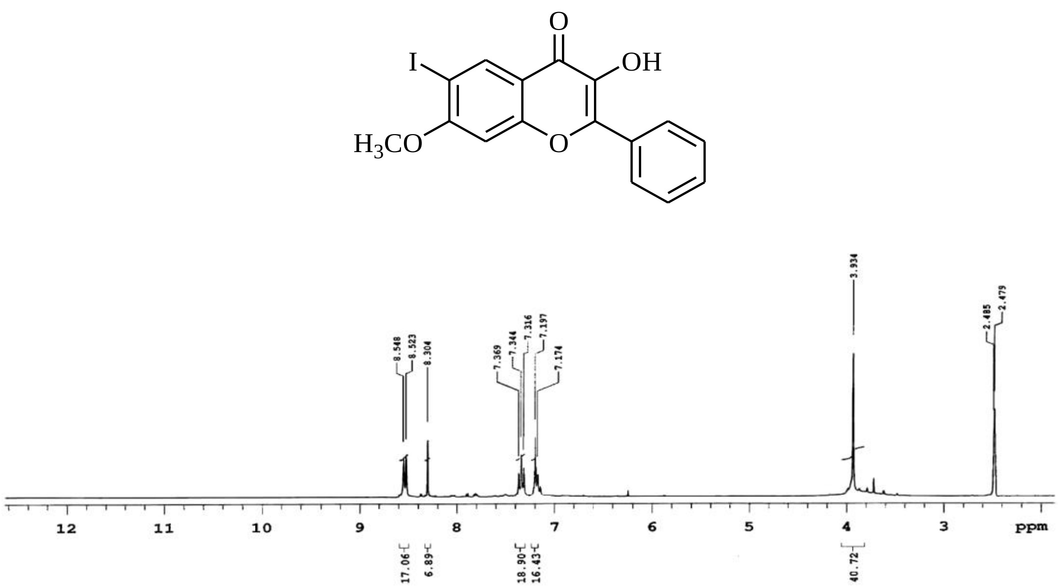

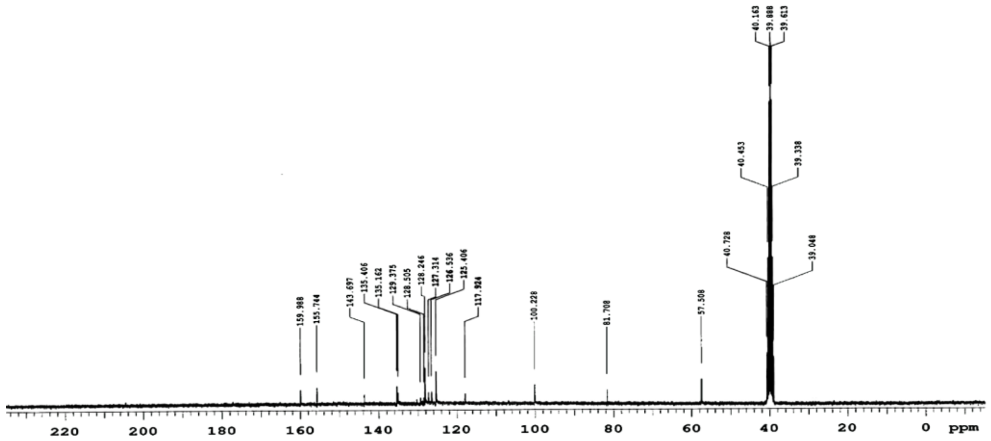

Figure S1.33: ^{1}H- and ^{13}C-NMR spectra of **3m** in DMSO-d_6 at 500 and 125 MHz, respectively.

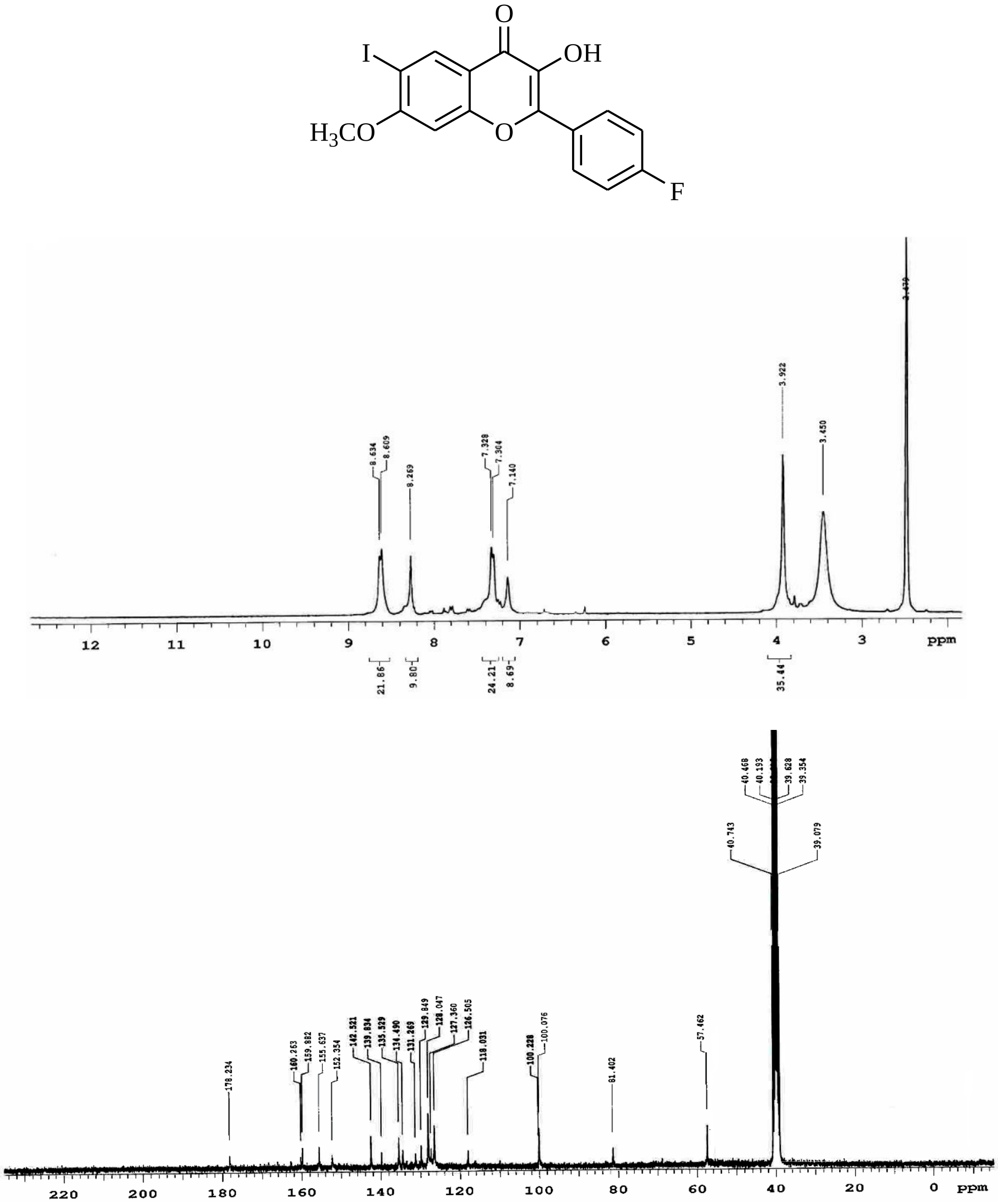

Figure S1.34: ^{1}H- and ^{13}C-NMR spectra of **3n** in DMSO-d_6 at 500 and 125 MHz, respectively.

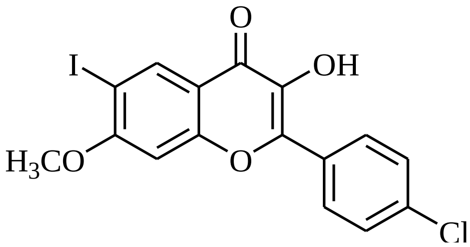

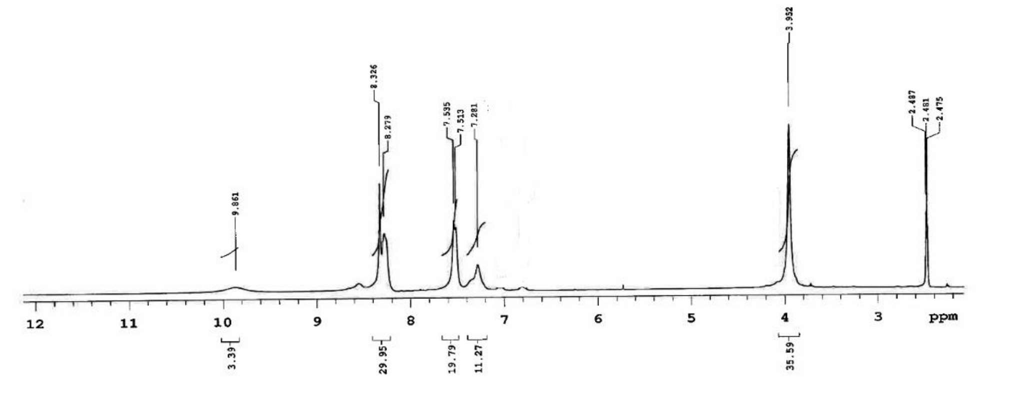

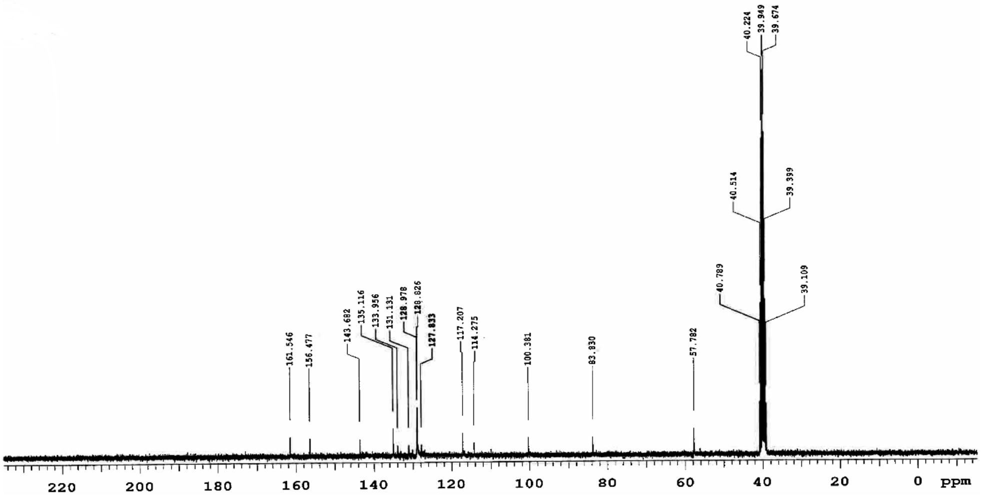

Figure S1.35: ^{1}H- and ^{13}C-NMR spectra of **3o** in DMSO-*d*6 at 500 and 125 MHz, respectively.

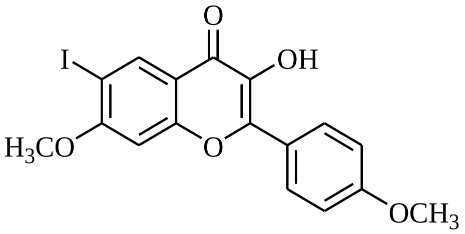

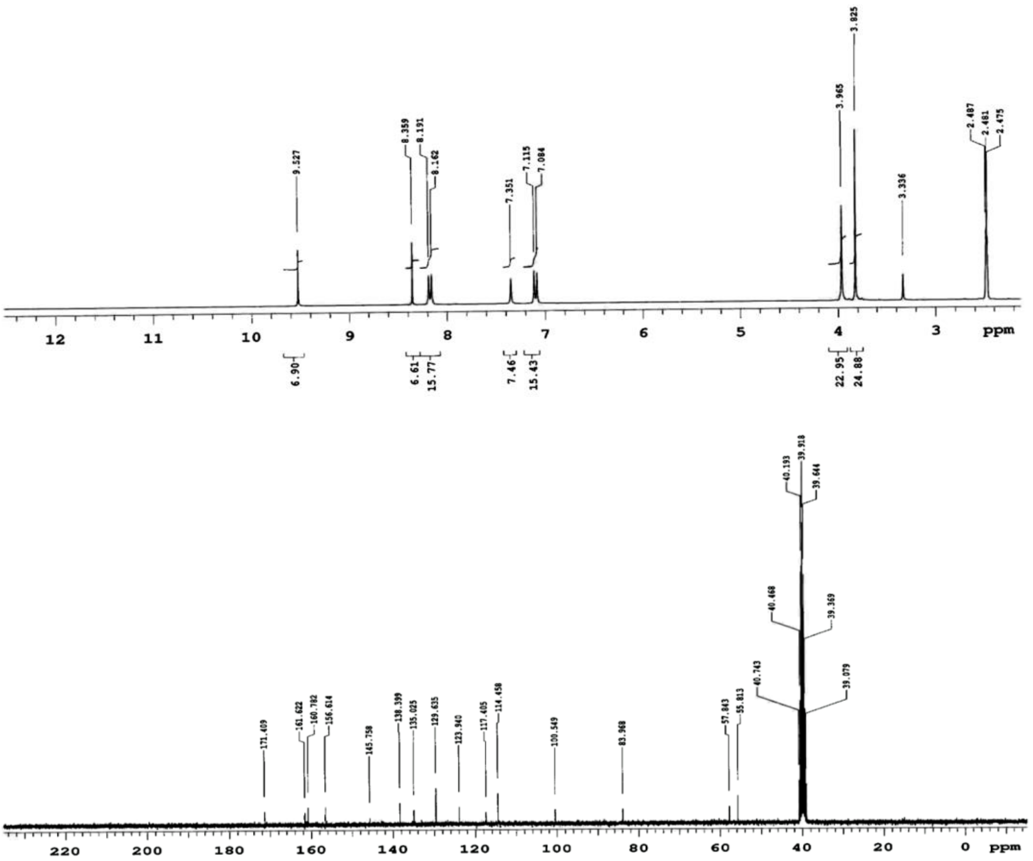

Figure S1.36: ^{1}H- and ^{13}C-NMR spectra of **3p** in DMSO-d_6 at 500 and 125 MHz, respectively.

Table S1. IC_{50} values for BACE-1 inhibitory effect of **2h**, **2j**, **2n**, **2p**, **3b**, **3c**, **3l**, **3p** & quercetin.

Compound	BACE-1
**2h**	13.82 ± 0.03
**2j**	4.703 ± 0.06
**2n**	25.07 ± 0.1
**2p**	70.79 ± 0.2
**3b**	32.18 ± 0.15
**3c**	19.69 ± 0.05
**3l**	15.74 ± 0.12
**3p**	22.44 ± 0.07
**Quercetin**	12.66 ± 0.02

Figure S2. Docking poses of compounds **2a–p** and **3a–p** against the active site of AChE.

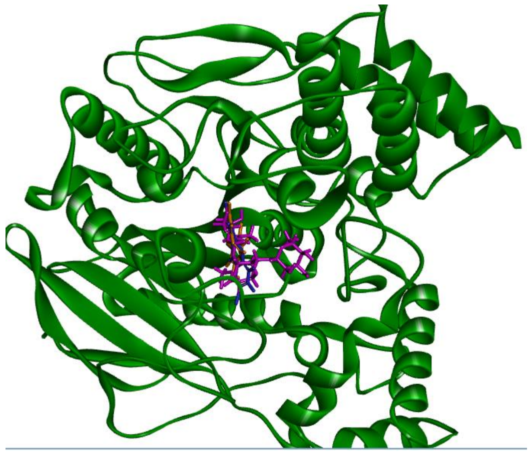

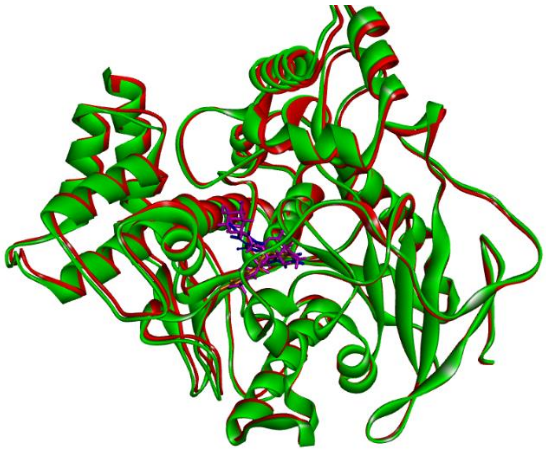

Figure S2.1. **1f**, **3b** and donepezil docked into 1e66 (green) and 1GQR (red) to show their positions of docking, Donepezil in purple, **1f** in orange and **3b** in blue. Protein is displayed in green.

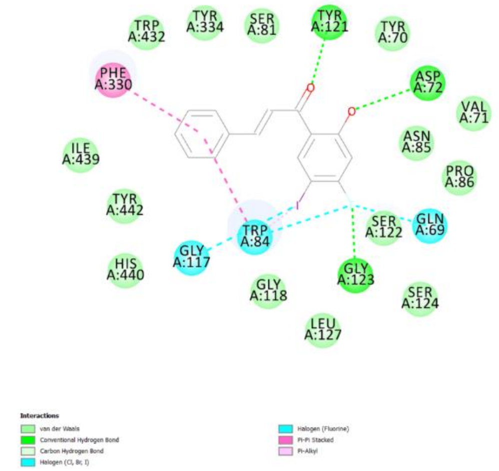

Figure S2.2. Docking pose of **2a** into the active site of AChE (PDB 1E66).

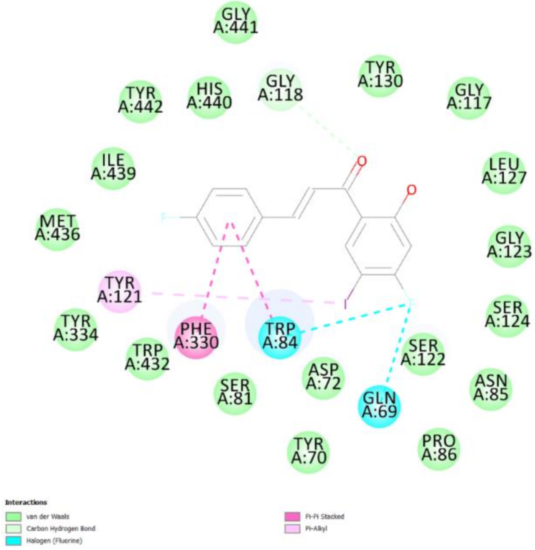

Figure S2.3. Docking pose of **2b** into the active site of AChE (PDB 1E66).

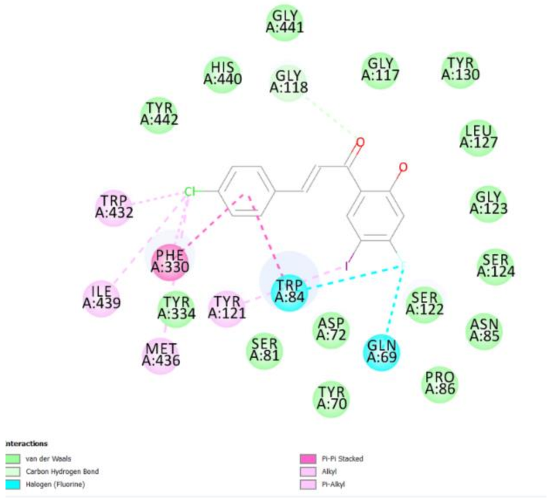

Figure S2.4. Docking pose of **2c** into the active site of AChE (PDB 1E66).

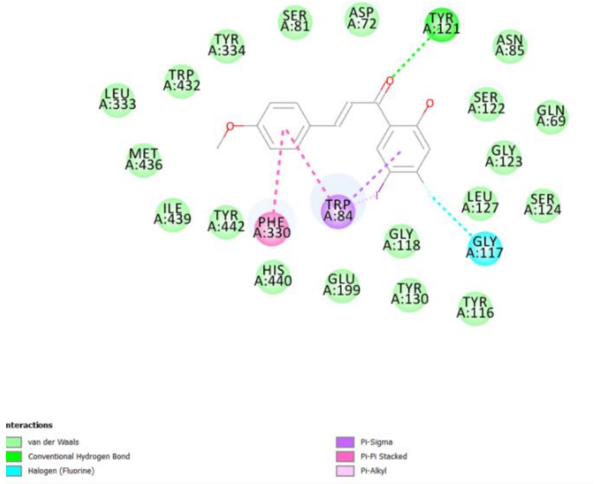

Figure S2.5. Docking pose of **2d** into the active site of AChE (PDB 1E66).

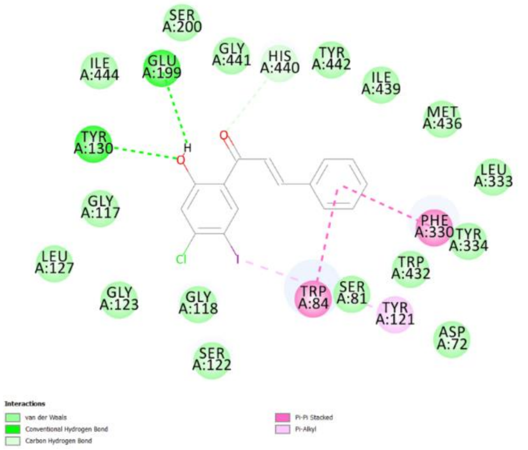

Figure S2.6. Docking pose of **2e** into the active site of AChE (PDB 1E66).

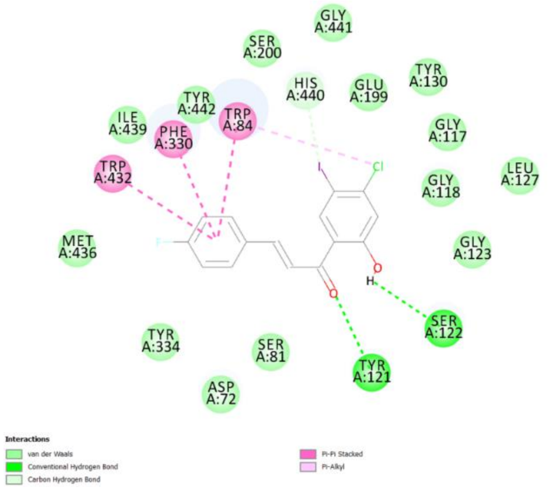

Figure S2.7. Docking pose of **2f** into the active site of AChE (PDB 1E66).

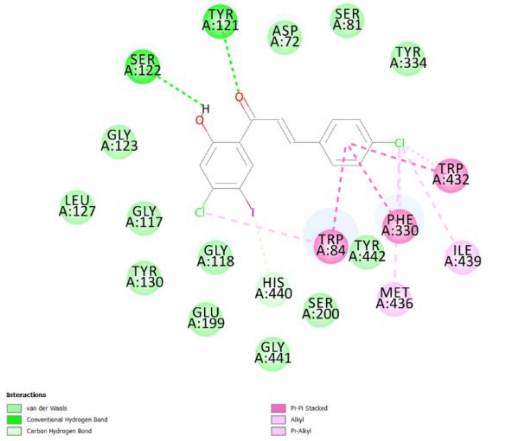

Figure S2.8. Docking pose of **2g** into the active site of AChE (PDB 1E66).

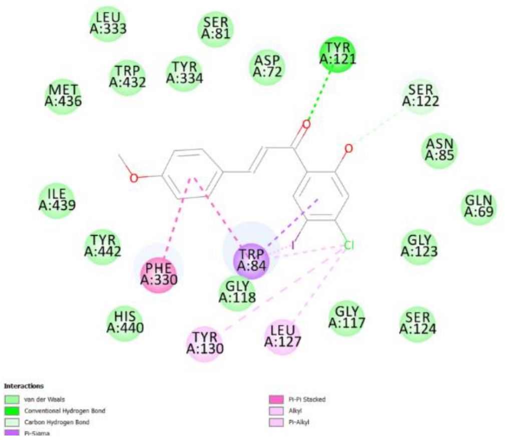

Figure S2.9. Docking pose of **2h** into the active site of AChE (PDB 1E66).

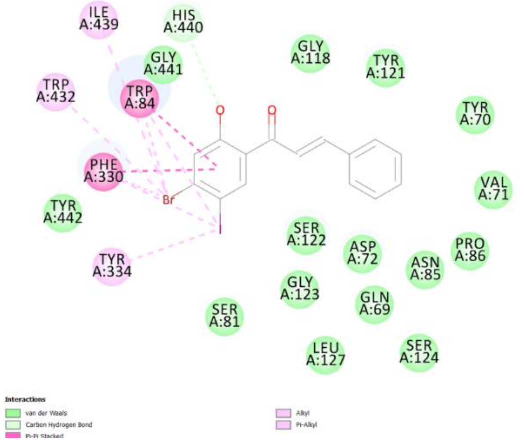

Figure S2.10: Docking pose of **2i** into the active site of AChE (PDB 1E66).

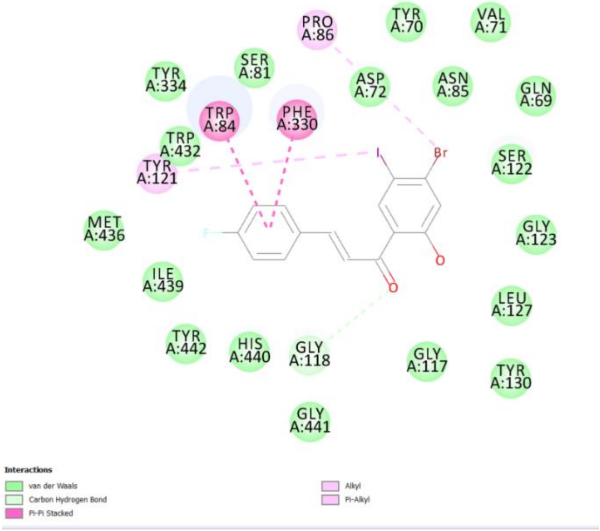

Figure S2.11: Docking pose of **2j** into the active site of AChE (PDB 1E66).

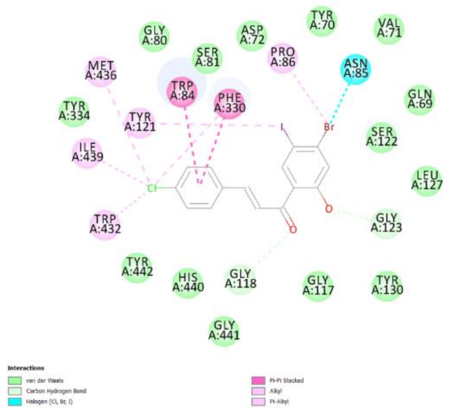

Figure S2.12: Docking pose of **2k** into the active site of AChE (PDB 1E66).

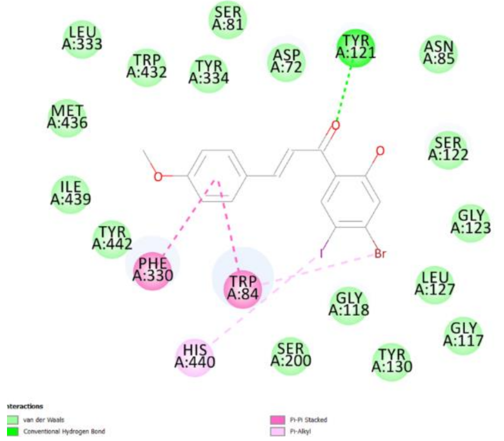

Figure S2.13: Docking pose of **2l** into the active site of AChE (PDB 1E66).

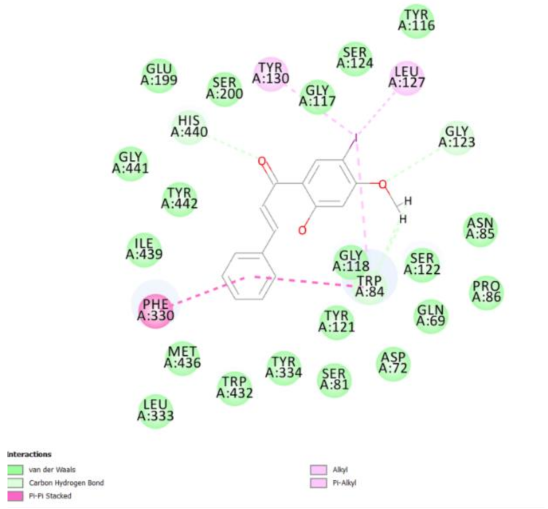

Figure S2.14: Docking pose of **2m** into the active site of AChE (PDB 1E66).

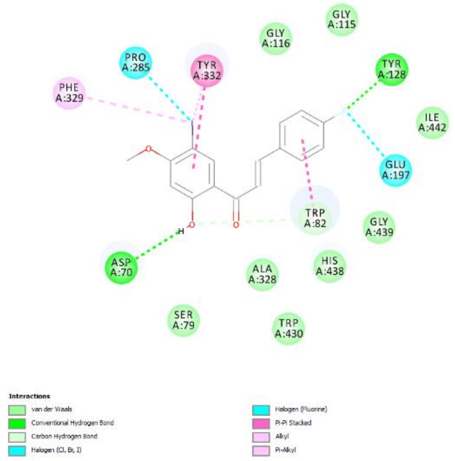

Figure S2.15: Docking pose of **2n** into the active site of AChE (PDB 1E66).

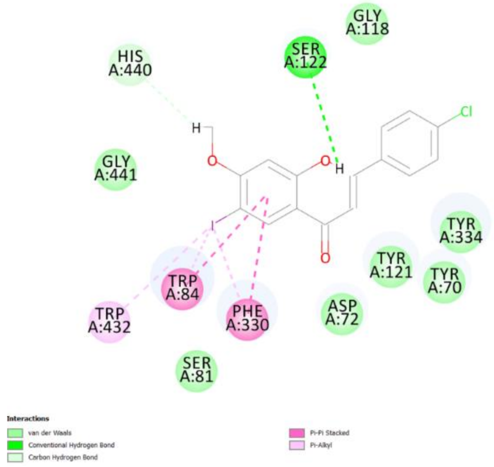

Figure S2.16: Docking pose of **2o** into the active site of AChE (PDB 1E66).

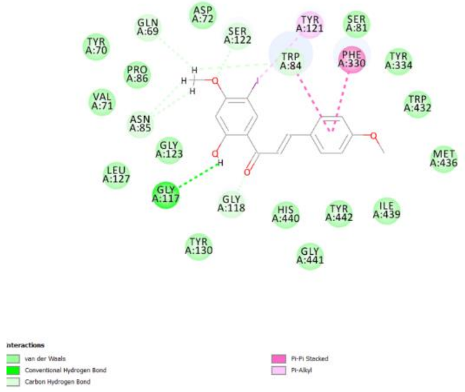

Figure S2.17: Docking pose of **2p** into the active site of AChE (PDB 1E66).

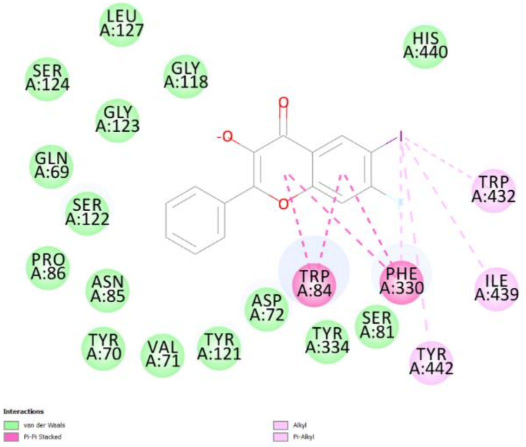

Figure S2.18: Docking pose of **3a** into the active site of AChE (PDB 1E66).

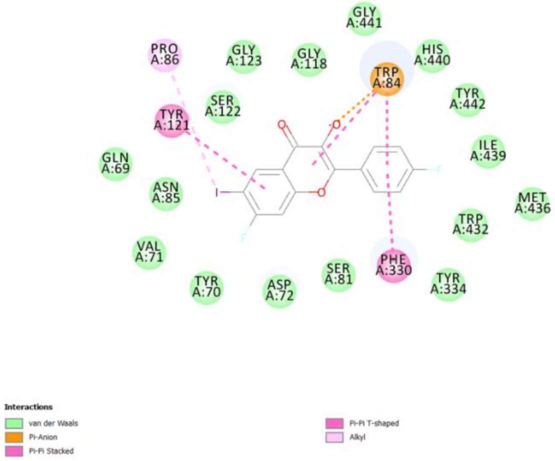

Figure S2.19: Docking pose of **3b** into the active site of AChE (PDB 1E66).

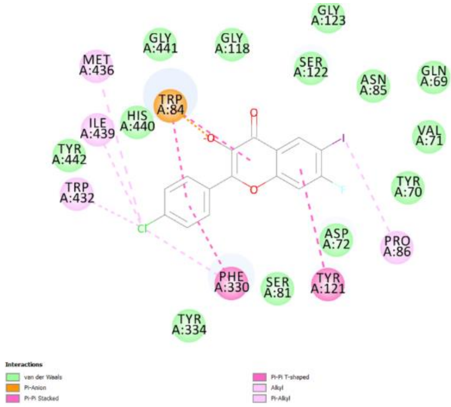

Figure S2.20: Docking pose of **3c** into the active site of AChE (PDB 1E66).

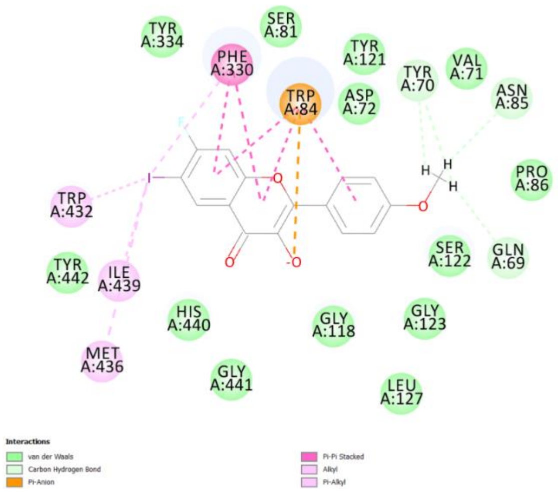

Figure S2.21: Docking pose of **3d** into the active site of AChE (PDB 1E66).

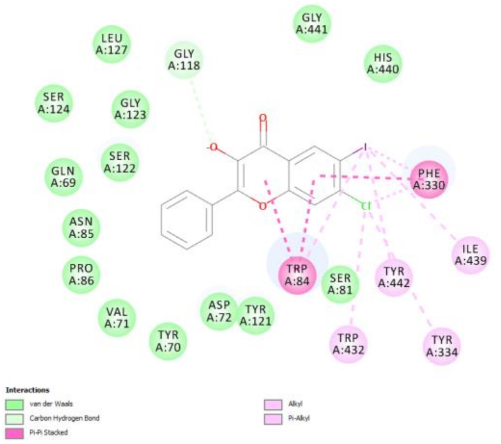

Figure S2.22: Docking pose of **3e** into the active site of AChE (PDB 1E66).

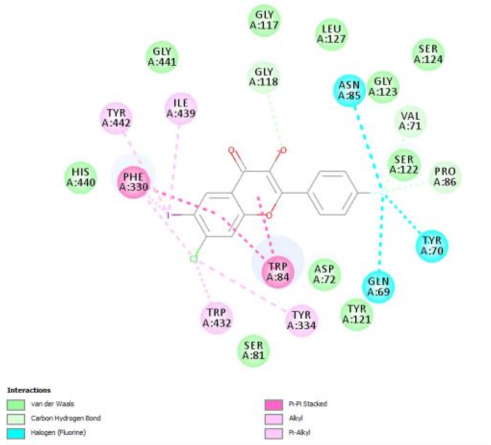

Figure S2.23: Docking pose of **3f** into the active site of AChE (PDB 1E66).

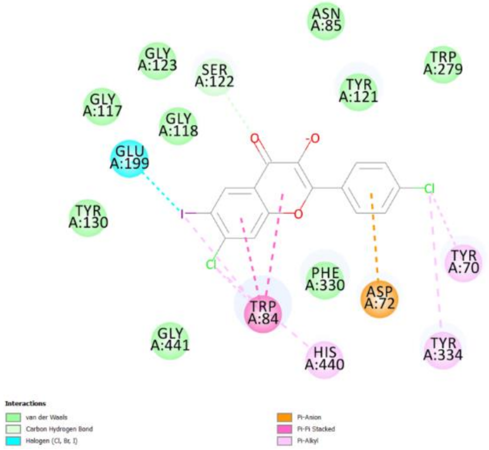

Figure S2.24: Docking pose of **3g** into the active site of AChE (PDB 1E66).

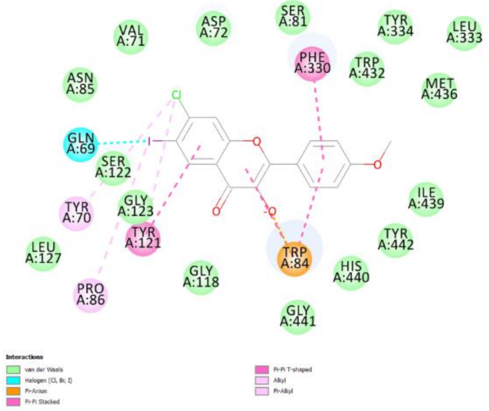

Figure S2.25: Docking pose of **3h** into the active site of AChE (PDB 1E66).

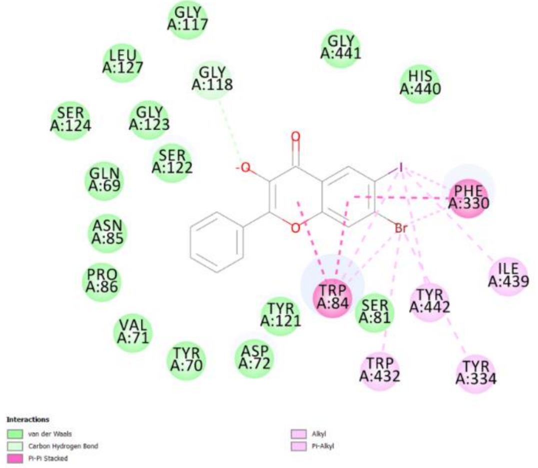

Figure S2.26: Docking pose of **3i** into the active site of AChE (PDB 1E66).

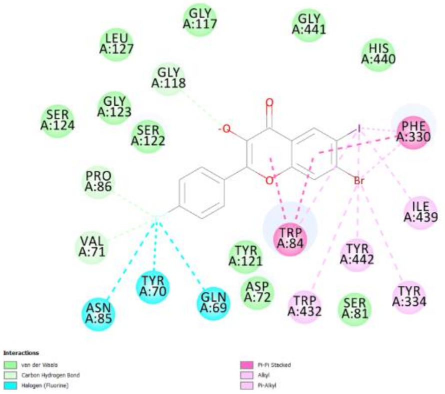

Figure S2.27: Docking pose of **3j** into the active site of AChE (PDB 1E66).

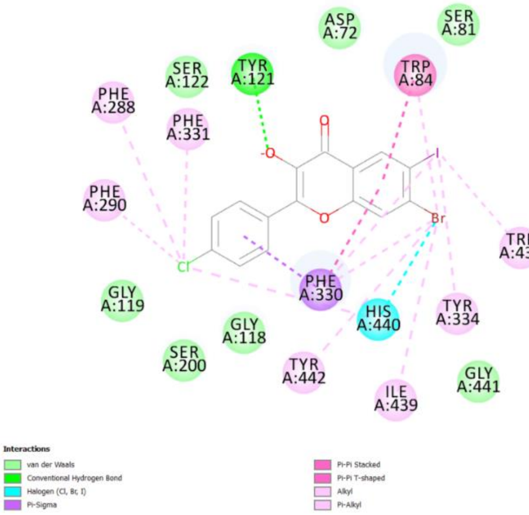

Figure S2.28: Docking pose of **3k** into the active site of AChE (PDB 1E66).

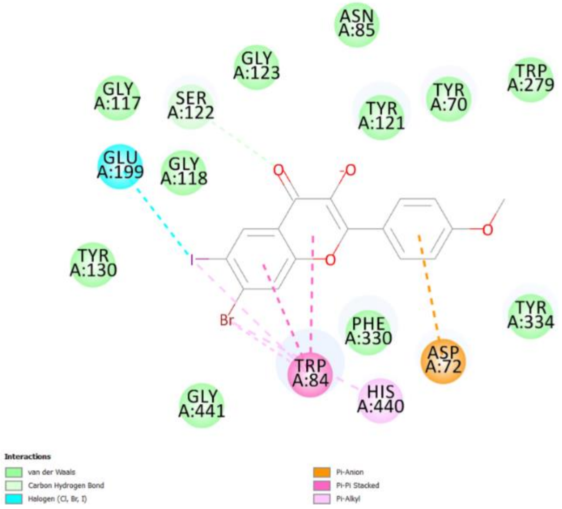

Figure S2.29: Docking pose of **3l** into the active site of AChE (PDB 1E66).

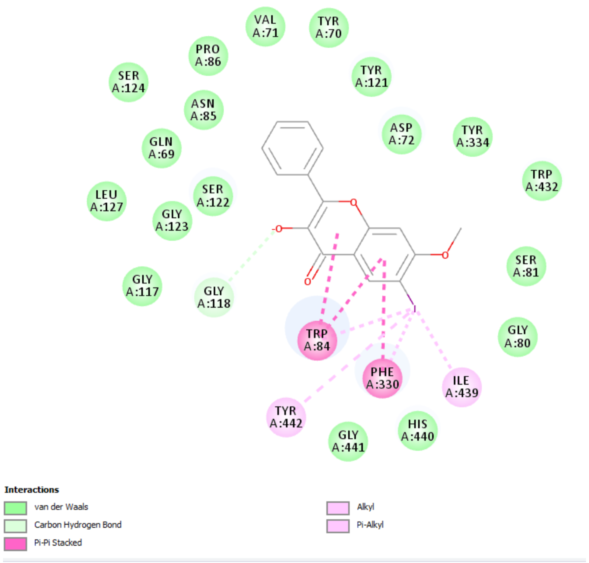

Figure S2.30: Docking pose of **3m** into the active site of AChE (PDB 1E66).

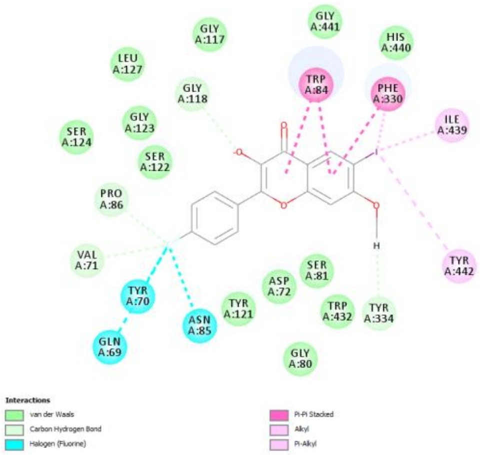

Figure S2.31: Docking pose of **3n** into the active site of AChE (PDB 1E66).

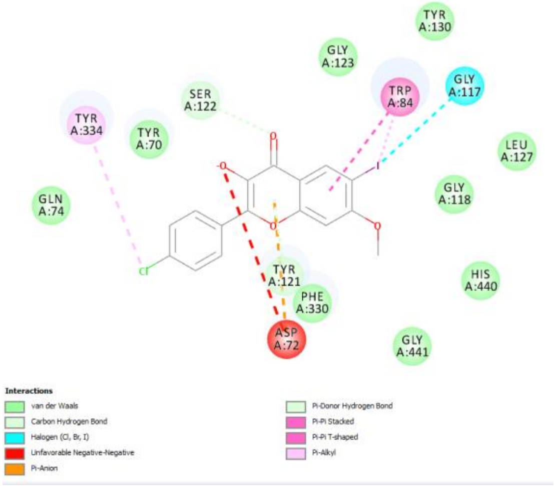

Figure S2.32: Docking pose of **3o** into the active site of AChE (PDB 1E66).

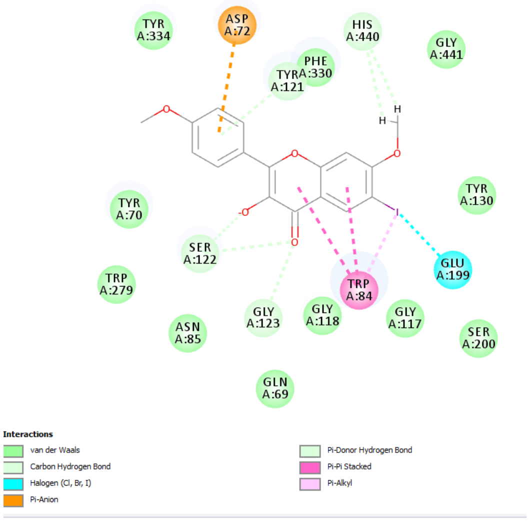

Figure S2.33: Docking pose of **3p** into the active site of AChE (PDB 1E66).

Figures S3. Docking poses of compounds **2a**–**p** and **3a**–**p** against the active site of BChE.

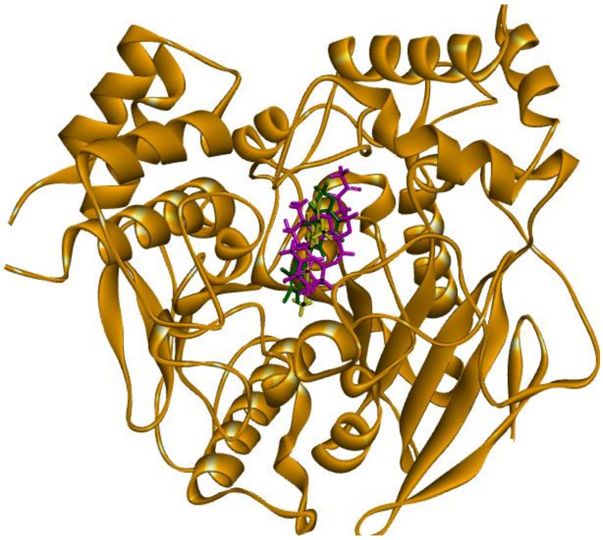

Figure S2.1. 2h, **3p** and donepezil docked into 1P0I to show their positions of docking.

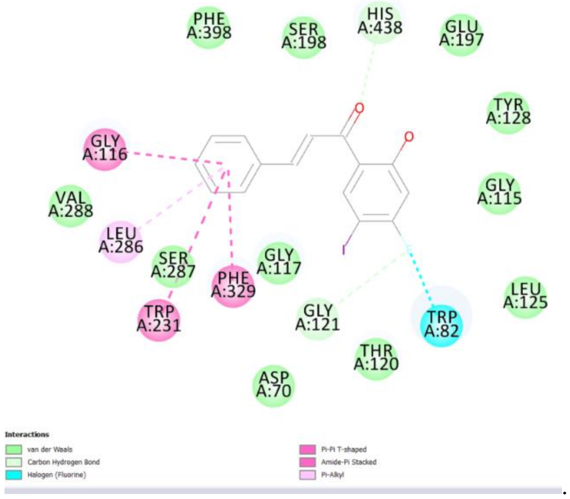

Figure S3.2. Docking pose of **2a** into the active site of BChE (PDB 1P0I).

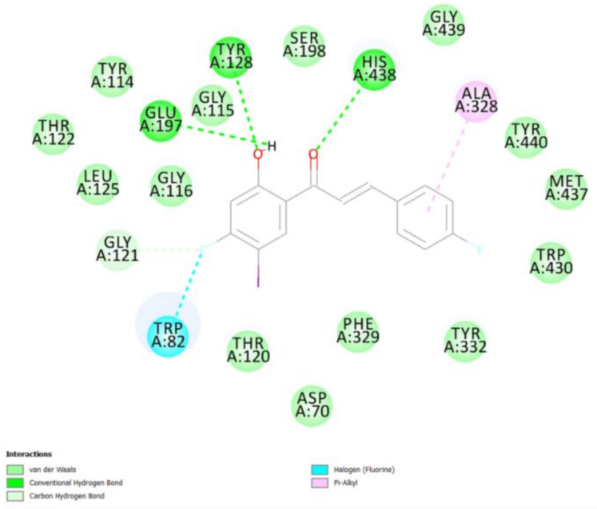

Figure S3.3. Docking pose of **2b** into the active site of BChE (PDB 1P0I).

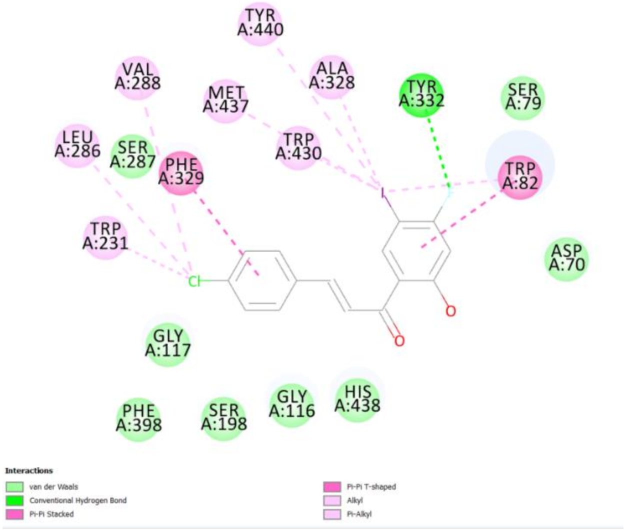

Figure S3.4. Docking pose of **2c** into the active site of BChE (PDB 1P0I).

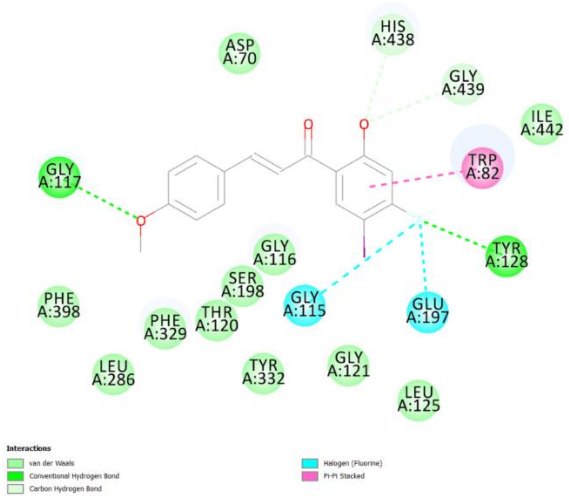

Figure S3.5. Docking pose of **2d** into the active site of BChE (PDB 1P0I).

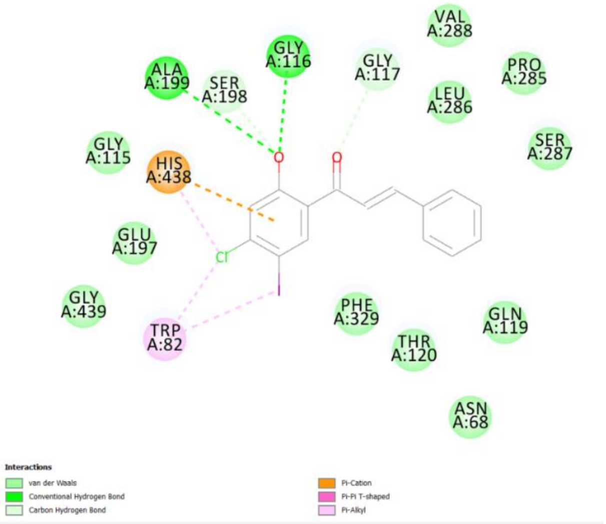

Figure S3.6. Docking pose of **2e** into the active site of BChE (PDB 1P0I).

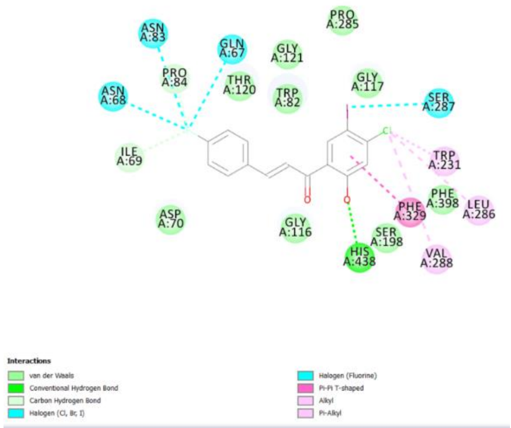

Figure S3.7. Docking pose of **2f** into the active site of BChE (PDB 1P0I).

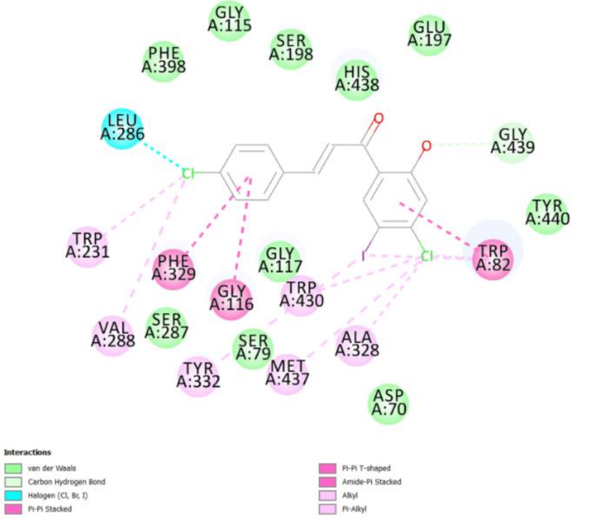

Figure S3.8. Docking pose of **2g** into the active site of BChE (PDB 1P0I).

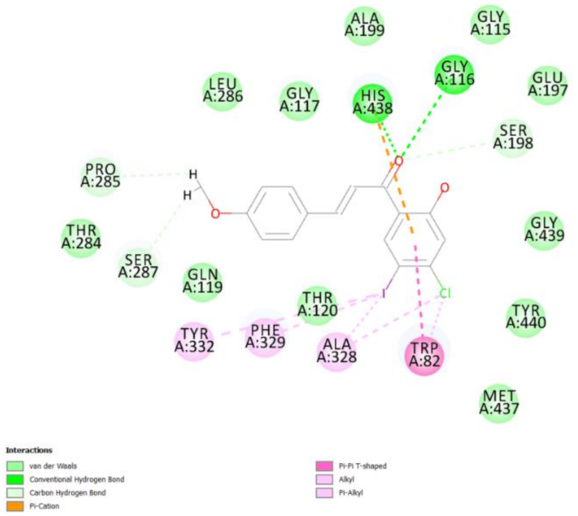

Figure S3.9. Docking pose of **2h** into the active site of BChE (PDB 1P0I).

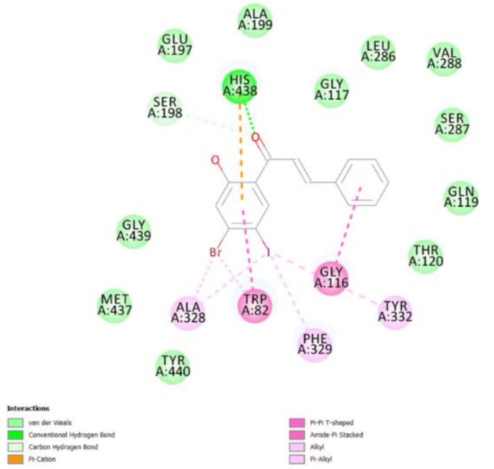

Figure S3.10: Docking pose of **2i** into the active site of BChE (PDB 1P0I).1P0I.

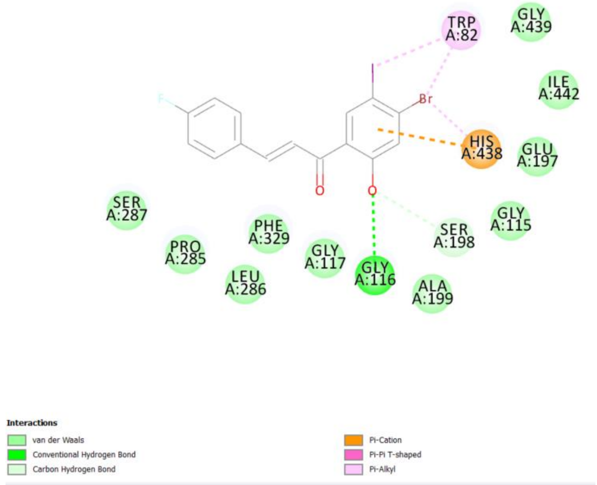

Figure S3.11: Docking pose of **2j** into the active site of BChE (PDB 1P0I).

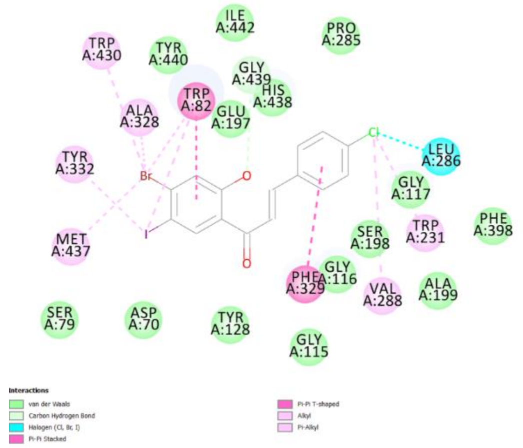

Figure S3.12: Docking pose of **2k** into the active site of BChE (PDB 1P0I).

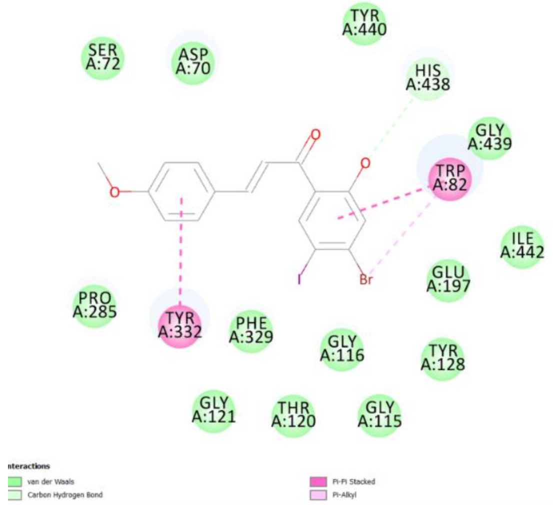

Figure S3.13: Docking pose of **2l** into the active site of BChE (PDB 1P0I).

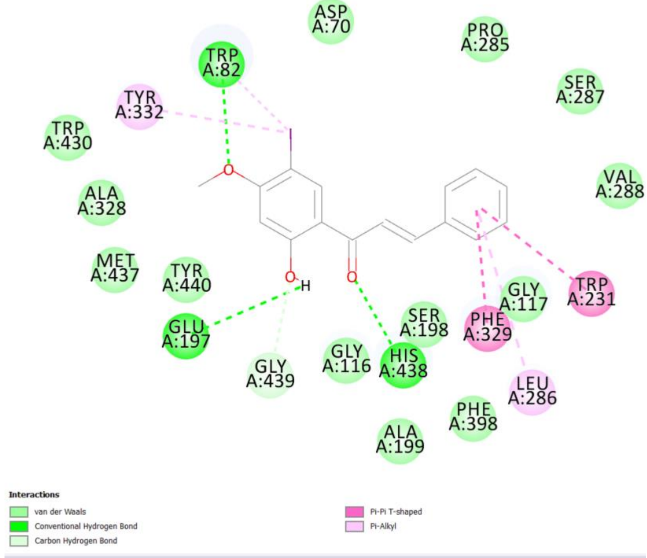

Figure S3.14: Docking pose of **2m** into the active site of BChE (PDB 1P0I).

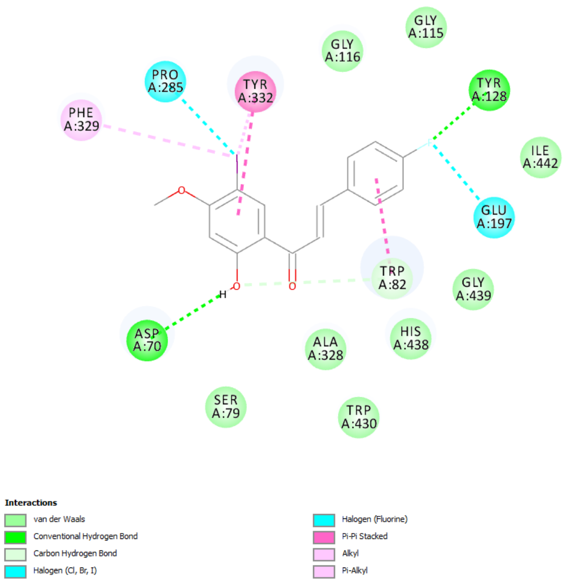

Figure S3.15: Docking pose of **2n** into the active site of BChE (PDB 1P0I).

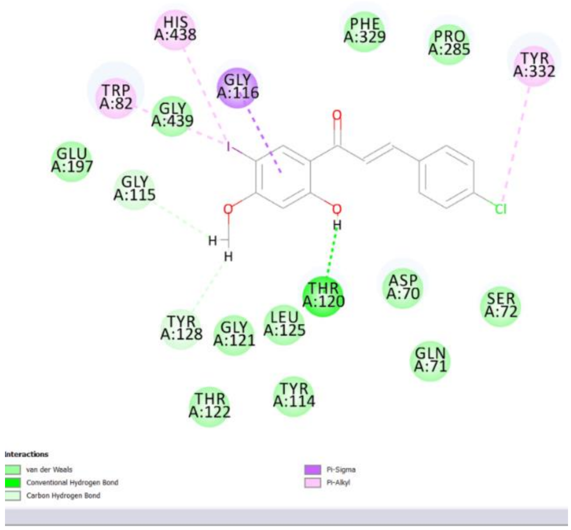

Figure S3.16: Docking pose of **2o** into the active site of BChE (PDB 1P0I).

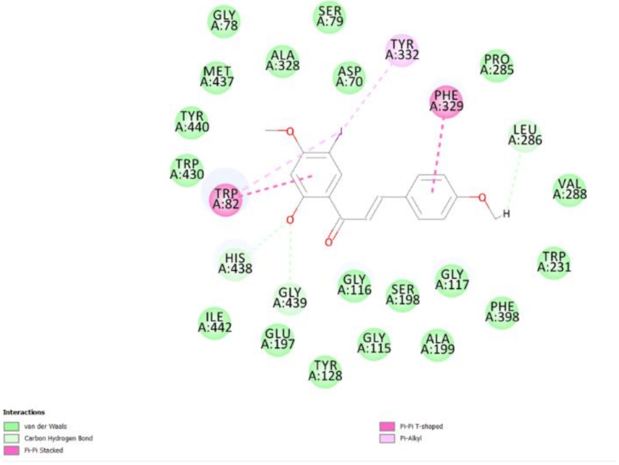

Figure S3.17: Docking pose of **2p** into the active site of BChE (PDB 1P0I).

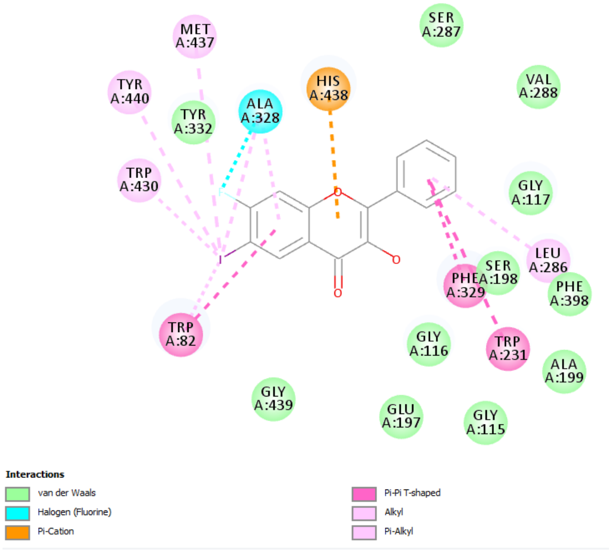

Figure S3.18: Docking pose of **3a** into the active site of BChE (PDB 1P0I).

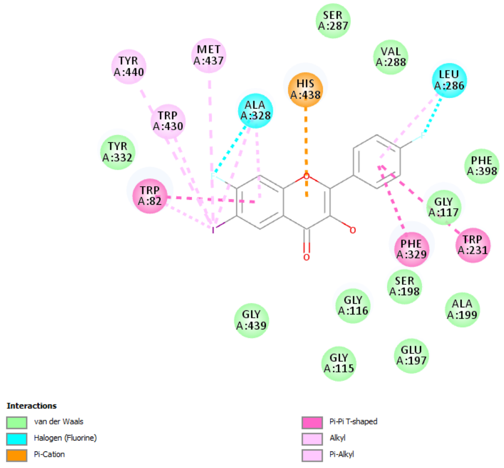

Figure S3.19: Docking pose of **3b** into the active site of BChE (PDB 1P0I).

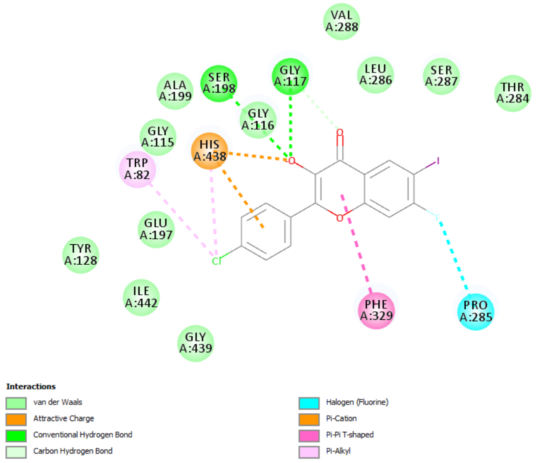

Figure S3.20: Docking pose of **3c** into the active site of BChE (PDB 1P0I).

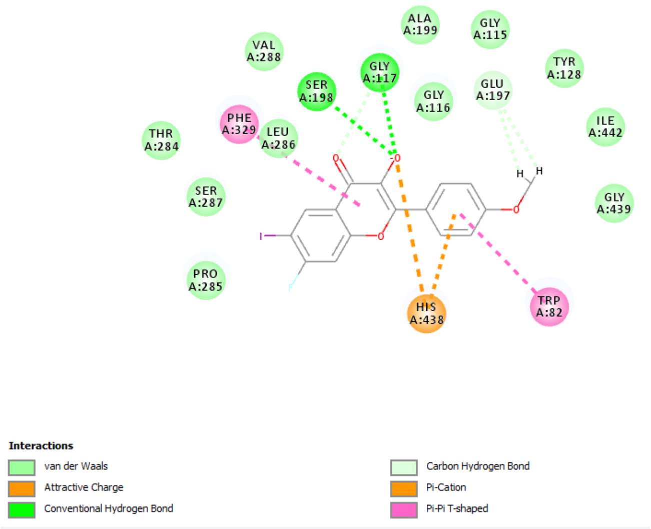

Figure S3.21: Docking pose of **3d** into the active site of BChE (PDB 1P0I).

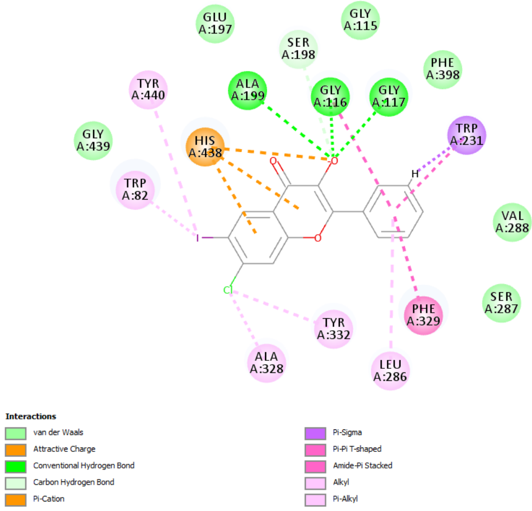

Figure S3.22: Docking pose of **3e** into the active site of BChE (PDB 1P0I).

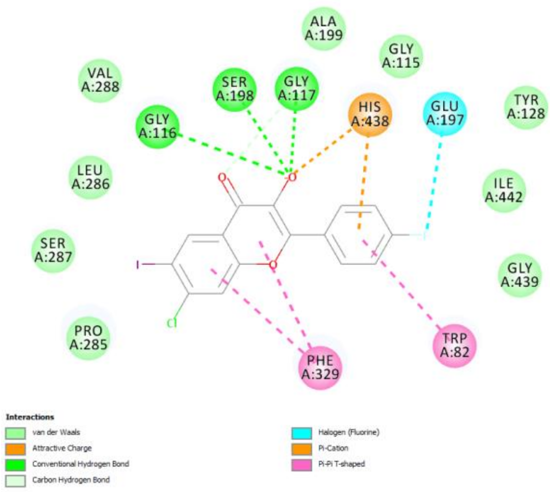

Figure S3.23: Docking pose of **3f** into the active site of BChE (PDB 1P0I).

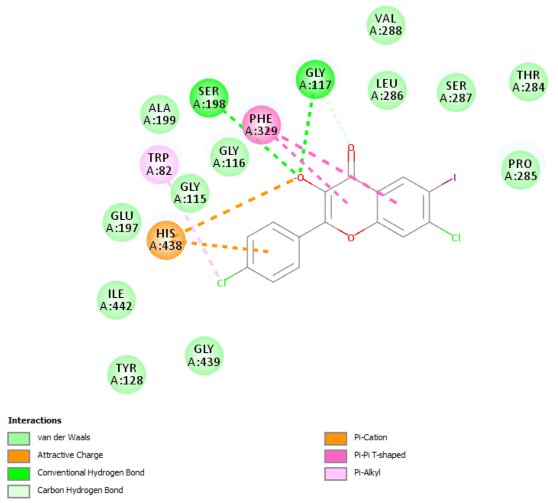

Figure S3.24: Docking pose of **3g** into the active site of BChE (PDB 1P0I).

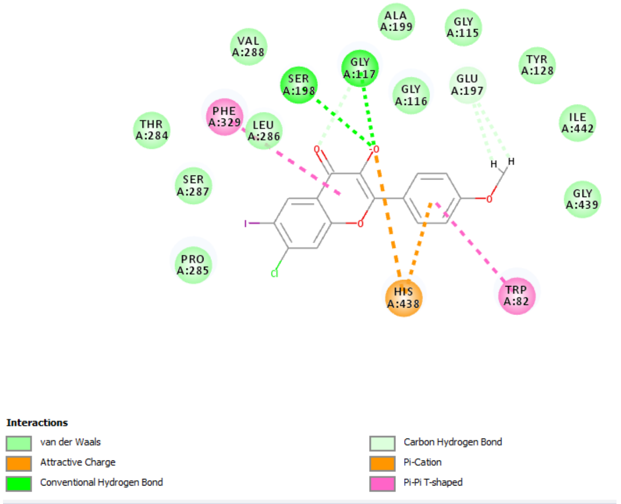

Figure S3.25: Docking pose of **3h** into the active site of BChE (PDB 1P0I).

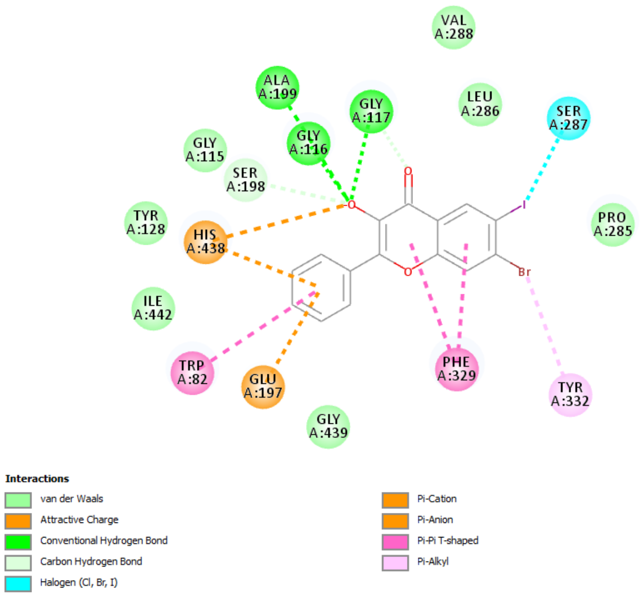

Figure S3.26: Docking pose of **3i** into the active site of BChE (PDB 1P0I).

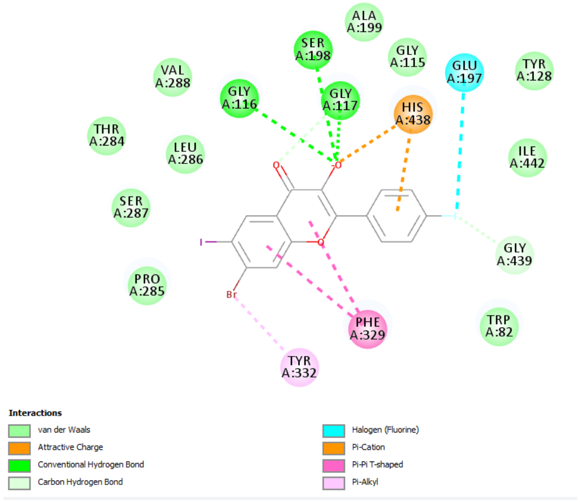

Figure S3.27: Docking pose of **3j** into the active site of BChE (PDB 1P0I).

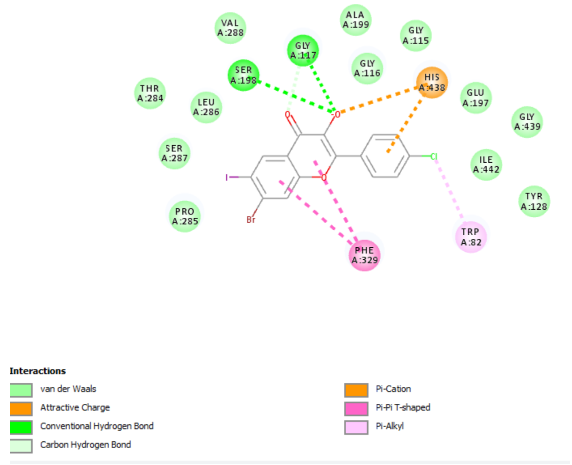

Figure S3.28: Docking pose of **3k** into the active site of BChE (PDB 1P0I).

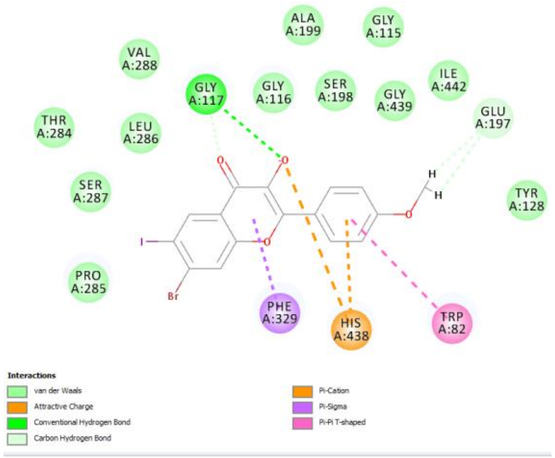

Figure S3.29: Docking pose of **3l** into the active site of BChE (PDB 1P0I).

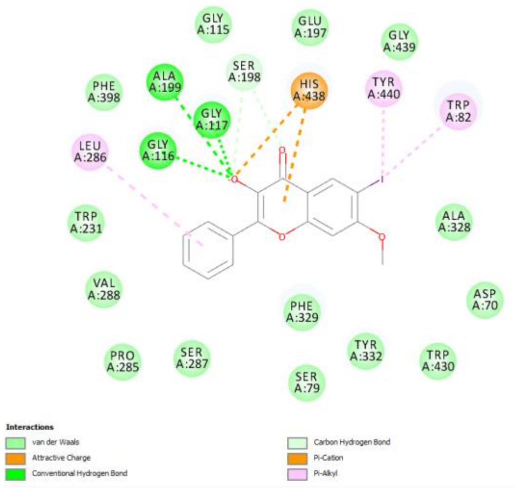

Figure S3.30: Docking pose of **3m** into the active site of BChE (PDB 1P0I).

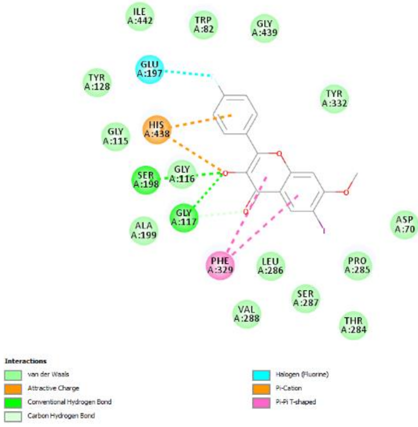

Figure S3.31: Docking pose of **3n** into the active site of BChE (PDB 1P0I).

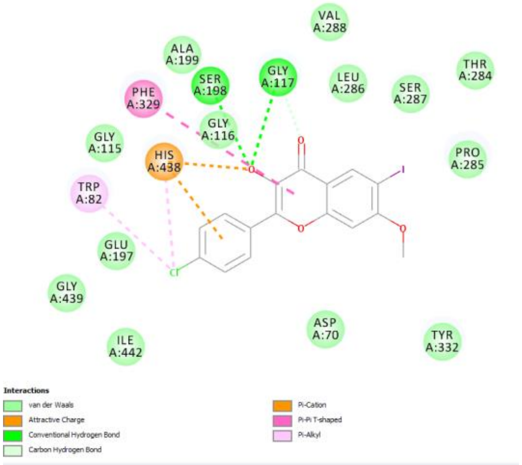

Figure S3.32: Docking pose of **3o** into the active site of BChE (PDB 1P0I).

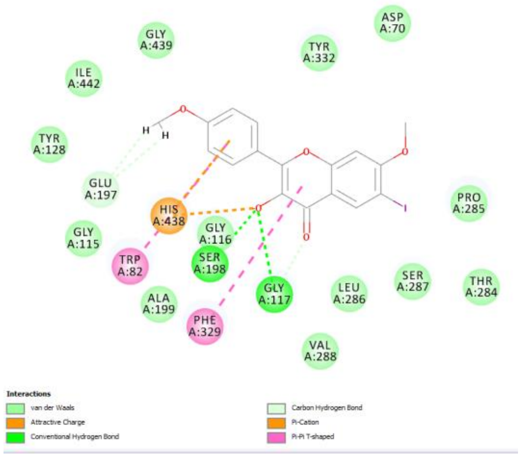

Figure S3.33: Docking pose of **3p** into the active site of BChE (PDB 1P0I).

Figure S4. Docking poses of **2n**, **2p**, **3b** and **3p** into BACE-1.

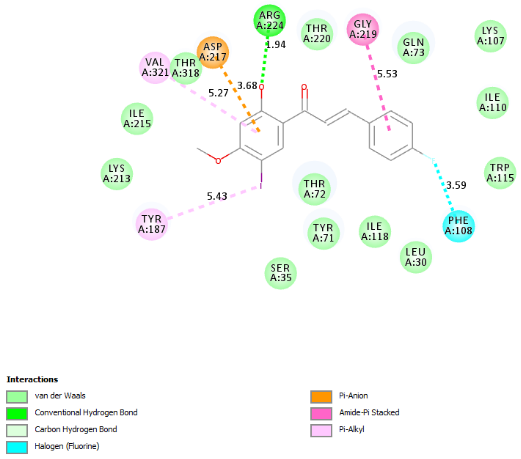

Figure S4.1. Docking pose of **2n** into BACE-1 (PDB code: 4D8C).

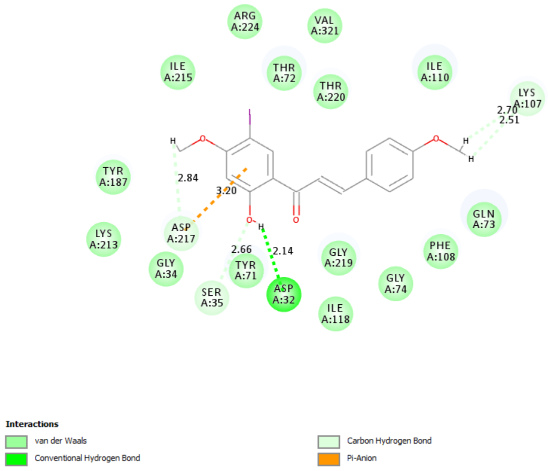

Figure S4.2. Docking pose of **2p** into BACE-1 (PDB code: 4D8C).

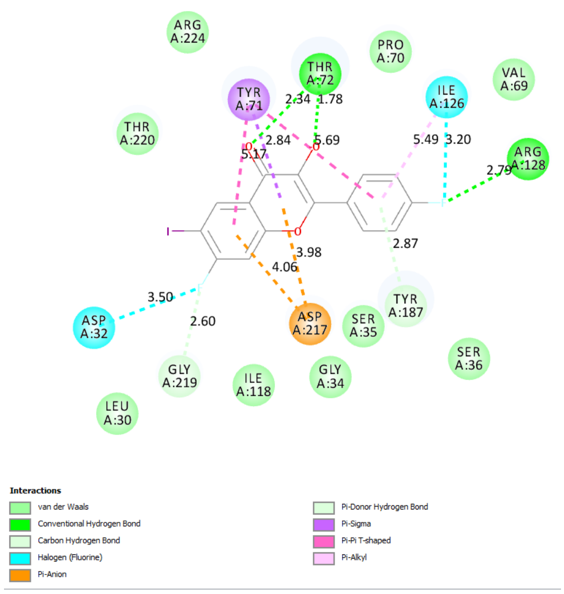

Figure S4.3. Docking pose of **3b** into BACE-1 (PDB code: 4D8C)

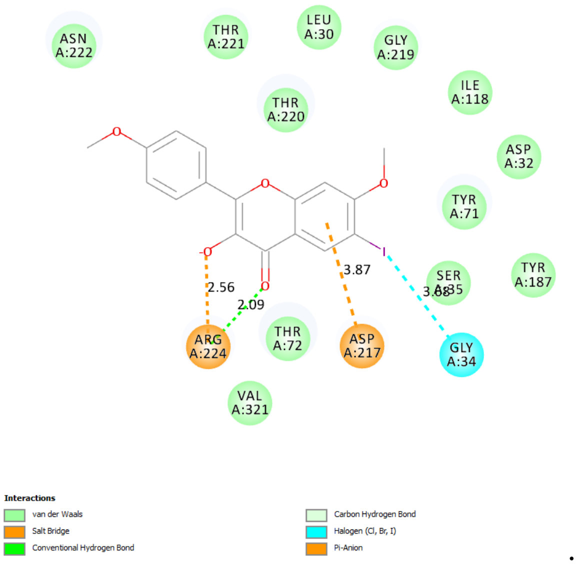

Figure S4.4. Docking pose of **3p** into BACE-1 (PDB code: 4D8C).
